# Investigating the role of source and source trust in prebunks and debunks of misinformation in online experiments across four EU countries

**DOI:** 10.1038/s41598-024-71599-6

**Published:** 2024-09-05

**Authors:** Hendrik Bruns, François J. Dessart, Michał Krawczyk, Stephan Lewandowsky, Myrto Pantazi, Gordon Pennycook, Philipp Schmid, Laura Smillie

**Affiliations:** 1 European Commission, Joint Research Centre (JRC), Rue du Champ de Mars 21, 1050 Brussels, Belgium; 2 European Commission, Joint Research Centre (JRC), Seville, Spain; 3 PRODEMINFO, University of Potsdam, Potsdam, Germany; 4 School of Psychological Science, University of Bristol, Bristol, UK; 5 Faculty of Social and Behavioural Sciences, University of Amsterdam, Amsterdam, The Netherlands; 6 Department of Psychology, Cornell University, Ithaca, USA; 7 Centre for Language Studies, Radboud University, Nijmegen, The Netherlands

**Keywords:** Psychology, Human behaviour

## Abstract

Misinformation surrounding crises poses a significant challenge for public institutions. Understanding the relative effectiveness of different types of interventions to counter misinformation, and which segments of the population are most and least receptive to them, is crucial. We conducted a preregistered online experiment involving 5228 participants from Germany, Greece, Ireland, and Poland. Participants were exposed to misinformation on climate change or COVID-19. In addition, they were pre-emptively exposed to a prebunk, warning them of commonly used misleading strategies, before encountering the misinformation, or were exposed to a debunking intervention afterwards. The source of the intervention (i.e. the European Commission) was either revealed or not. The findings show that both interventions change four variables reflecting vulnerability to misinformation in the expected direction in almost all cases, with debunks being slightly more effective than prebunks. Revealing the source of the interventions did not significantly impact their overall effectiveness. One case of undesirable effect heterogeneity was observed: debunks with revealed sources were less effective in decreasing the credibility of misinformation for people with low levels of trust in the European Union (as elicited in a post-experimental questionnaire). While our results mostly suggest that the European Commission, and possibly other public institutions, can confidently debunk and prebunk misinformation regardless of the trust level of the recipients, further evidence on this is needed.

## Introduction

Misinformation is prevalent today, especially in relation to crises such as the climate crisis and the COVID-19 pandemic. Climate change misinformation includes doubts about human involvement in global warming, the denial of global warming’s existence and the rejection of the scientific consensus^1^. Similarly, the COVID-19 pandemic was accompanied by misinformation from the start, including narratives that questioned its existence, downplayed its severity, promoted unproven remedies and cast doubt on the efficacy of vaccination^2^.

In addition to the threats posed by crises themselves, misinformation around crises threatens societies and makes it more difficult for public institutions to address these crises. Believing in COVID-19 misinformation can discourage protective behaviour^3,4^, including vaccination^5^, with potentially life-threatening consequences^6^. Exposure to climate change misinformation decreases prosocial behaviour and acceptance of scientific facts^7^. Addressing and managing misinformation has therefore become a crucial component of an effective crisis response, particularly when that misinformation jeopardises public discourse, institutional integrity and public health^8^.

Public institutions have access to science-based interventions to combat misinformation, including debunks and prebunks^9^. Debunks involve exposing false information and refuting it with credible sources after exposure to misinformation^10–12^. Prebunks, on the other hand, proactively warn individuals about misinformation before exposure, refute often-used erroneous arguments and explain strategies commonly used in spreading false information^13–22^.

Both prebunking and debunking interventions have been found to be effective in reducing the threat of misinformation^11,13,14,17,21–26^. This paper addresses four main gaps in the literature, with four corresponding research questions. First, although exceptions exist^27–29^, prebunking and debunking interventions have typically been investigated separately, leading to scarce evidence on their relative effectiveness. In this paper, we compare the relative effectiveness of the two approaches, providing valuable insights to enable public institutions and policymakers select the most efficient interventions in times of crisis.

Second, existing evidence on the impact of the source of these interventions on their effectiveness is still inconclusive. People evidently consider the source when assessing the credibility of information^30,31^ and misinformation^32,33^. They appear to do so also for debunks^33–37^. However, the role of source information in relation to prebunks is unclear. This paper aims to uncover whether or not revealing the source of an intervention against misinformation modifies its effectiveness. We use the European Commission as the source of the intervention in the experiment, due to the major role that this institution played in the fight against COVID-19 misinformation in the European Union (EU)^38^.

Third, people’s trust in the source of misinformation-countering interventions may be fundamental to their success, and yet there is a lack of research looking into this. This study examines whether the effectiveness of misinformation-countering interventions depends on recipients’ levels of trust in the EU (i.e. the source of our interventions). Trust in the EU is a commonly assessed measure, used here as an indicator of trust in the European Commission, which is a common source of public campaigns such as those aimed at combating misinformation. Source credibility may matter more to some people than others, and recent findings suggest that tailored interventions taking perceived credibility into account may be worthwhile^39^.

Fourth, much of the available evidence is based on US samples. For instance, almost 70% of studies featured in a debunking meta-analysis from 2018 examined North American samples^23^. Consequently, there is a burgeoning literature on the effects of corrections cross-nationally^25,40–43^. Joining this literature, this study uses a wide, non-US, multi-country sample to achieve broad generalisability of the findings. More specifically, our experiment involved 5228 participants from four EU Member States–Germany, Greece, Ireland and Poland–which we selected to achieve diversity in terms of average perceived frequency of encountering misinformation and confidence in being able to spot it. In addition, this study examines the efficacy of prebunking and debunking interventions for combating misinformation on two topics (i.e. COVID-19 and climate change) rather than just one, and it includes comprehensive outcome variables, capturing not only self-reported beliefs but also intention to share misinformation online or offline, publicly or with close contacts, and differentiating between endorsing and condemning the content as motives for sharing.

## Results

In this section, we report on the effects of debunks and prebunks from different sources on 5228 respondents’ agreement with, credibility assessment of and intentions to agree and disagree with the following six claims, of which each participant encountered one in the form of an online newspaper article: (1) “It hasn’t warmed since 1998”; (2) “There is no scientific consensus on climate change”; (3) “Climate models are unreliable”; (4) “The Covid-19 vaccine does not work”; (5) “The Covid-19 vaccine has not been properly tested in clinical trials”; (6)“ “The Covid-19 vaccine is dangerous”. More information on the selection of these claims can be found in the “Methods” section.

**Fig. 1:**
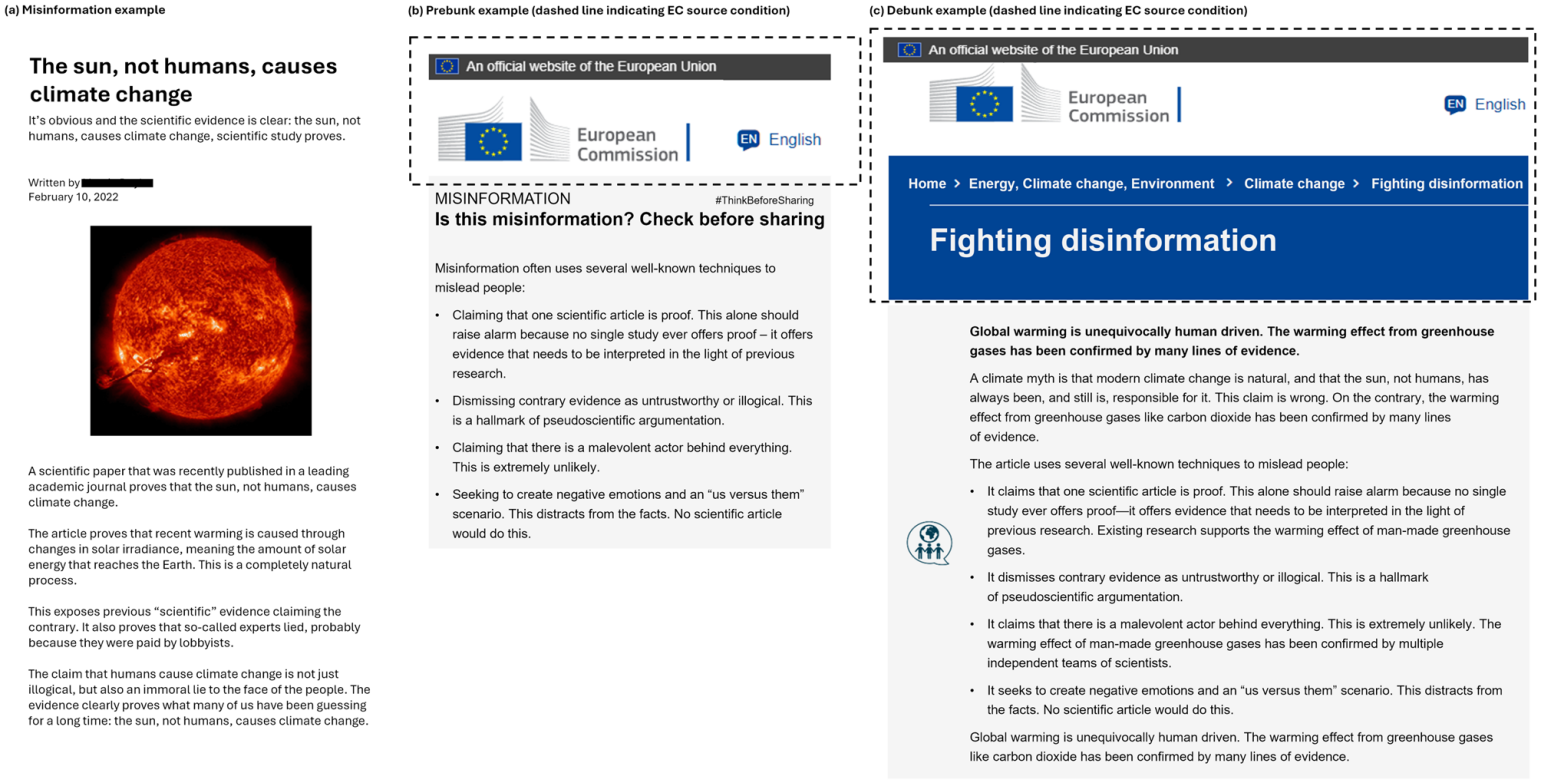
Examples of a (**a**) misinformation article, (**b**) prebunk and (**c**) debunk used in the experiment. The parts of the prebunks and debunks that were present only in the treatments in which the source was the European Commission are surrounded by dashed lines. Copyright for the flag of European Commission and related screenshots from the website of the European Commission are owned by the European Commission, all rights reserved. Note that these websites are solely for the purpose of the experiment and do not exist in this format. Image credit for image of the sun (left): NASA/SDO.

Participants randomly received no intervention, a prebunk prior to their exposure to misinformation or a debunk after their exposure to misinformation. Debunks and prebunks could furthermore come from an unspecified source or from the European Commission. Afterwards, participants reported their levels of agreement with the main claim of the article, assessed its credibility and indicated their intention to share the article agreeing or disagreeing with it. Examples of a misinformation article, prebunk and debunk used in the experiments are shown in (Fig. 1).

We analyse the effects on agreement with the main claim using an ordered logit model, on credibility assessment using an ordinary least squares (OLS) model and on changes in sharing intention to agree and disagree using binary logit models.

### Do debunks and prebunks work?

**Fig. 2:**
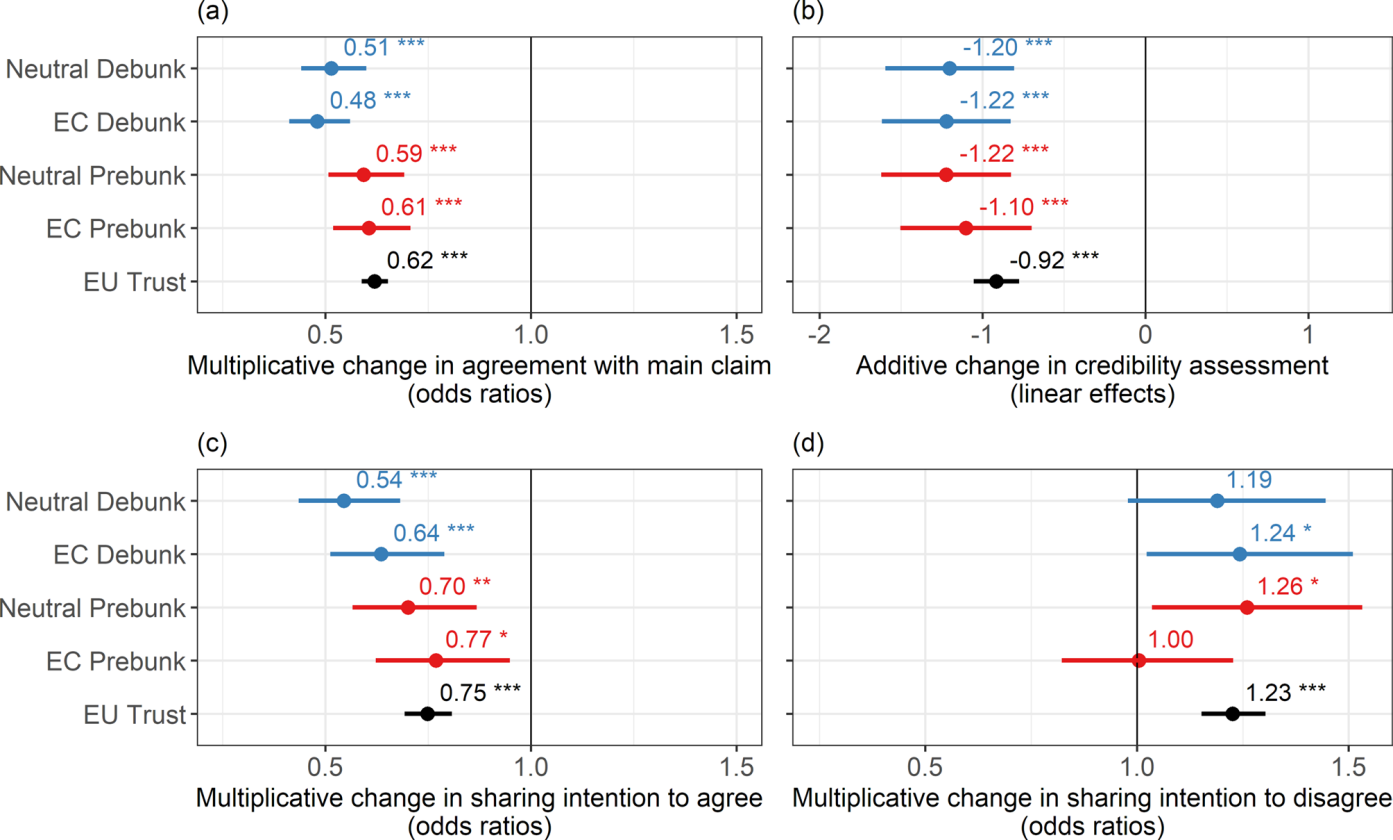
Effects of debunks and prebunks revealing (i.e. *EC* European Commission) or not revealing (i.e. neutral) the source of the intervention on the main outcome variables. The *y*-axis shows the four experimental treatments (with the control as the reference condition) and standardised trust in the EU (which is not a treatment variable). The *x*-axis shows the changes in the four main outcome variables. (**a**) Shows the effects on agreement with the main claim shown in the misleading article from an ordered logistic regression as odds ratios; (**b**) shows the effects on the credibility assessment of the misleading article from a linear OLS regression as linear estimates; (**c**) shows the effects on intention to share the misleading article to express agreement with it (i.e. ‘sharing intention to agree’) from a binary logistic regression as odds ratios; (**d**) shows the effects on intention to share the misleading article to express disagreement with it (i.e. ‘sharing intention to disagree’) from a binary logistic regression as odds ratios. Effects of debunks are shown in blue and those of prebunks in red. Bars represent heteroscedasticity-robust 95% confidence intervals. Significance levels: ***p < 0.001, **p < 0.01, *p < 0.05.

As specified in the preregistration plan, all analyses will be pooled across topics and misinformation articles. Compared with the control condition, where no prebunking or debunking intervention was provided, all four interventions significantly and substantially reduced agreement with the misleading article’s claim (Fig. 2a, with detail in Table S-10 of the supplementary material). These effects are substantial, approximately halving the odds of strongly agreeing with the main (false) claims. There is also a significant association (i.e. main effect) between trust in the EU (mean-centred and standardised) and agreement: participants with higher levels of trust in the EU were less likely to agree with the claim. A one-standard-deviation increase in EU trust had an effect identical to that of the prebunks. Average marginal effects (AMEs) are substantial, with a reduction in the likelihood of (strong) agreement with the false claim of around 4 to 7 percentage points (pp) and an increase in the likelihood of (strong) disagreement of around 3 to 12 pp. All corresponding AMEs are shown in (Table S-7) in the supplementary material.

Debunks and prebunks had significant, negative and substantial effects on the credibility assessment of the misleading article, regardless of the source (Fig. 2b). Again, there is a significant (main) negative association with trust in the EU. All interventions successfully reduced credibility assessment by more than 1 point on the credibility scale (which ranges from 0 to 16). In addition, a one-standard-deviation increase in the level of trust in the EU was associated with a decrease of almost 1 point on the credibility rating scale, slightly below the intervention effects.

The interventions made participants significantly less likely to intend to share the misleading article to express their agreement, as shown in (Fig. 2c). Neutral debunks reduced the odds of intention to share to show agreement by almost half compared with the control (no intervention) treatment (corresponding to an AME of −9.15 pp), followed by European Commission debunks with an odds ratio of 0.64 (−7.14 pp). The effects of the two prebunks were also significant (neutral prebunk: AME = −5.76 pp; EC prebunk: AME = −4.37 pp). Importantly, the neutral debunk was significantly more effective than the neutral prebunk (OR = 0.78, CI_95_ = (0.61–0.99), *p* = 0.038, AME = −3.39 pp), but the EC debunk was not more effective than the EC prebunk (OR = 0.83, CI_95_ = (0.66–1.04), *p* = 0.102, AME = −2.77 pp). Similarly to the previous outcome variables, participants with high levels of trust in the EU displayed lower levels of intention to agree with the misleading article with an effect size similar to that of the EC prebunk.

For participants’ willingness to share or discuss the misleading article to express disagreement (Fig. 2d), the effects are less pronounced than for the previous outcomes. Two interventions increased the likelihood of such an intention, but the effects are weaker than for other outcome variables. In particular, the EC debunk and the neutral prebunk slightly increased the likelihood of participants wanting to share the misleading article to express disagreement (respectively by 4.22 and 4.5 pp). However, the neutral debunk and the EC prebunk did not have a significant impact, although the effect of the neutral debunk becomes significant when controlling for all covariates specified in the preregistration (OR = 1.39, CI_95_ = (1.08–1.79), *p* = 0.01, AME = 6.28 pp). As expected, a higher level of trust in the EU was associated with a higher likelihood of sharing the misleading article to disagree with it, with similar intensity to the two effective interventions.

The main effects presented above remain robust when accounting for all specified covariates in the preregistration (see Table S-22 in the supplementary material). Robustness checks involved performing main analyses with additional controls for subject characteristics including country of origin, responses to comprehension check questions, correct recall of the intervention source, and the misinformation topic (more detail is provided in the ‘Methods’ section).

Although not specified in the preregistration, we performed exploratory analyses to understand potential cross-country differences in the observed effects (see Figure S-10 in the supplementary material for the country-specific effects and Table S-4 and Table S-5 for random-effect models). These exploratory analyses revealed that (1) effects on agreement with the main claim hold almost throughout all four countries; (2) effects on credibility assessment appear in Germany and Poland, to a lesser extent in Ireland, and not in Greece; (3) effects on intention to agree are most significant in Poland, with some effects occurring in Ireland and Greece, but no significant effects in Germany; and (4) effects on intention to disagree were significant in Poland only.

### Do debunks or prebunks work better?

**Fig. 3:**
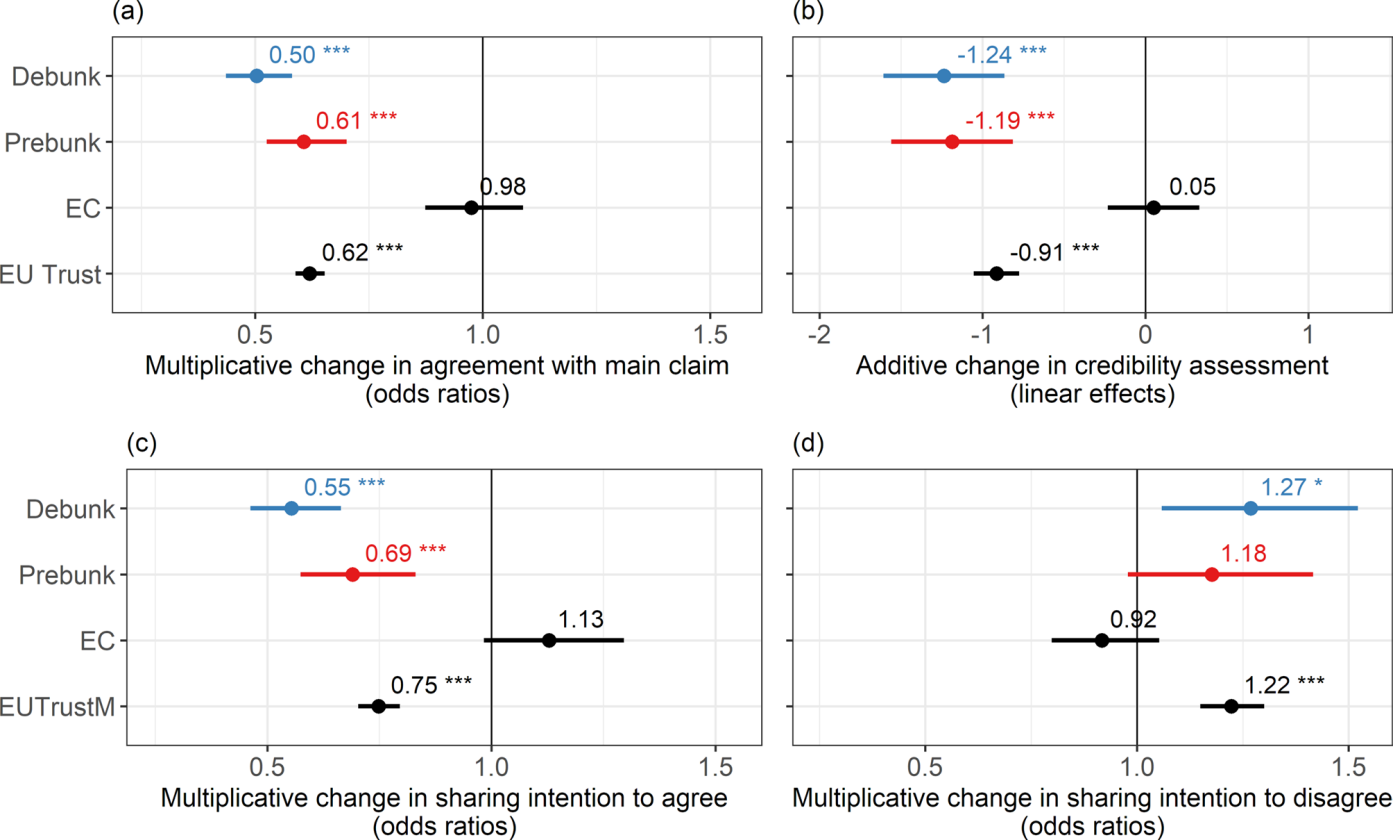
Effects of debunks, prebunks and revealing the European Commission (i.e. EC) as the intervention source on the main outcome variables. The *y*-axis shows the interventions (with the control as the reference condition), the Commission as the source of the intervention (with neutral, i.e. no source as the reference condition) and standardised trust in the EU (which is not a treatment variable). The *x*-axis shows the changes in the four main outcome variables. (**a**) Shows the effects on agreement with the main claim shown in the misleading article from an ordered logistic regression as odds ratios; (**b**) shows the effects on the credibility assessment of the misleading article from a linear OLS regression as linear estimates; (**c**) shows the effects on intention to share the misleading article to express agreement with it (i.e. ‘sharing intention to agree’) from a binary logistic regression as odds ratios; (**d**) shows the effects on intention to share the misleading article to express disagreement with it (i.e. ‘sharing intention to disagree’) from a binary logistic regression as odds ratios. Effects of debunks are shown in blue and those of prebunks in red. Bars represent heteroscedasticity-robust 95% confidence intervals. Significance levels: ***p < 0.001, **p < 0.01, *p < 0.05.

Controlling for the source and the level of trust in the EU, we observe two significant differences between debunks and prebunks (see Fig. 3). First, debunks are more effective than prebunks in reducing agreement with the main claim (OR = 0.83, CI_95_ = (0.74–0.92), *p* = 0.001). Agreement and strong agreement with the claims are reduced by 2 and 1 pp, respectively, with increasing likelihood of (strong) disagreement by 3.6 and 0.7 pp, respectively. Second, debunks are more effective in decreasing the likelihood of sharing to express agreement with the false claim (OR = 0.803, CI_95_ = (0.68–0.95), *p* = 0.008, AME = – 3.09 pp). However, there are no significant differences regarding the other two outcome variables, namely credibility assessment (E = 0.05, CI_95_ = (−0.23–0.33), *p* = 0.725) and intention to share to express disagreement with the misleading article (OR = 0.92, CI_95_ = (0.80–1.05), *p* = 0.218, AME = −1.5 pp), where the two interventions appear to perform equally well. Overall, these results suggest that debunks have a (very) small advantage over prebunks in addressing misinformation, to the extent that both are comparable (see the ‘Methods’ section for a discussion of their comparability). All corresponding AMEs are shown in Table S-8 in the supplementary material.

### Does revealing the source change the effectiveness of debunks and prebunks?

Figure 2 already compared the effects of both European Commission and neutral debunks and prebunks. To further explore this, Fig. 3 presents the effects of EC-source compared with the neutral source (i.e. no source), controlling for the type of intervention and the level of trust in the EU. Detailed results can be found in Table S-11 in the supplementary material. As can be seen, the estimates for EC source are non-significant across all outcome variables, indicating that, overall, EC-source does not significantly alter the effectiveness of the interventions in influencing the main outcome variables.

Exploratory analyses of country-specific effects reveal consistent null effects of revealing the European Commission as the source of either intervention. The only exception is a negative effect in Greece on the sharing intention to disagree with the article (see Figure S-11 in the supplementary material for these country-specific effects).

### Does the effect of revealing the source vary based on people’s levels of trust in the source?

We also tested the preregistered interaction effects between the source of the debunk and prebunk (i.e. the European Commission) and participants’ reported level of trust in the EU. The detailed values can be found in Table S-12 and Table S-13 in the supplementary material. The regression analyses incorporate an interaction term between the treatment variable and the mean-centred EU trust level. Therefore, the predictors for the treatment variable represent its effect for people with average levels of trust in the EU, while the EU trust level variable indicates the association between trust in the EU and the outcome variable for individuals in the reference treatment group (i.e. receiving neutral debunks or prebunks). Note that the EU trust level was measured after the main task and thus may have been influenced by the treatment assignment. We elaborate on the methodological choice of eliciting the trust level after the treatment assignment in the discussion section. Yet, we stress already here the importance of carefully interpreting these findings, as this method can bias treatment estimates^44^.

**Fig. 4:**
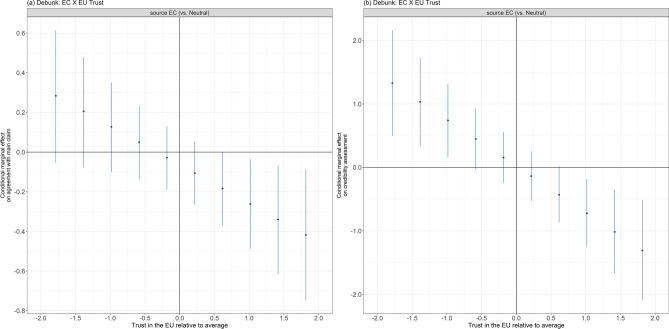
Conditional marginal effects for models with a significant interaction between the level of trust in the EU and revealing the source of the intervention (i.e. *EC* European Commission v neutral–no source). (a) Shows the change in agreement with the main claim resulting from providing the Commission as the source conditional on the level of trust in the EU (which is not a treatment variable) based on an ordered logistic regression; (b) shows the change in credibility assessment resulting from providing the Commission as the source conditional on the level of trust in the EU based on an ordered logistic regression. The EU trust level is de-meaned and standardised such that 0 on the *x*-axis corresponds to the average level of trust in the EU and 1 unit on the *x*-axis corresponds to one standard deviation. Bars represent heteroscedasticity-robust 95% confidence intervals.

Figure 4 illustrates the conditional marginal effects for the only cases with significant interaction effects between source information and EU trust on belief in the claim and credibility assessment of the misleading article, in both cases for debunks (see Figure S-12 in the supplementary material for the complete information). Specifically, as the level of trust in the EU increases, European Commission debunks are more effective in reducing agreement with the main claim compared with neutral debunks. This effect is prominent among respondents with high levels of EU trust. However, no such interaction effect is observed for prebunks. The interaction effect for debunks diminishes and becomes insignificant when all preregistered covariates are included (OR = 0.87, CI_95_ = (0.71–1.07), *p* = 0.192). Conditional effects are not robust for controlling the false discovery rate, which is recommended when conducting multiple hypothesis tests at different levels of the conditioning variable (in our case, the level of trust in the EU)^45^.

A more pronounced interaction effect is observed on perceived credibility. As the level of trust in the EU increases, the Commission debunk is more effective than the neutral debunk in reducing the perceived credibility of the misleading article. This effect is evident in panel (b), where the Commission source decreases the perceived credibility of the misleading article for the debunking intervention among individuals with high levels of trust in the EU, but is counterproductive among individuals with low levels of trust in the EU. This significant interaction effect remains robust when all covariates are included (b = −0.51, CI_95_ = (−0.94–−0.08), *p* = 0.019). Conditional effects remain significant when adjusting confidence intervals to control the false discovery rate.

No significant interactions were found for participants’ intentions to share the misleading article, whether to express agreement or disagreement, for either debunks or prebunks. These results remain robust when controlling for the specified control variables outlined in the preregistration and detailed in the “Methods” section. Detailed estimates including control variables can be found in Table S-23 and Table S-24 in the supplementary material.

Investigating country-specific effects (shown in Figure S-13–Figure S-16 in the supplementary material) reveals that (1) the negative interaction between the Commission source and the EU trust level for debunks on agreement with the main claim (panel (a) in Fig. 4) does not occur in any of the individual countries but is rather a result of the narrower confidence intervals after pooling the data; (2) the negative interaction between the Commission source and the EU trust level for debunks on credibility assessment is only significant in Greece, with coefficients in other countries in the same direction but much smaller; (3) there are some additional minor country-specific interaction effects, such as for the effect of debunks on intention to disagree in Germany, of debunks on credibility assessment in Greece and of prebunks on agreement with the main claim in Ireland. With respect to conditional effects, we furthermore note slight country-specific differences in comparison with the pooled data.

### Can perceptions of debunks and prebunks explain the effects?

**Fig. 5:**
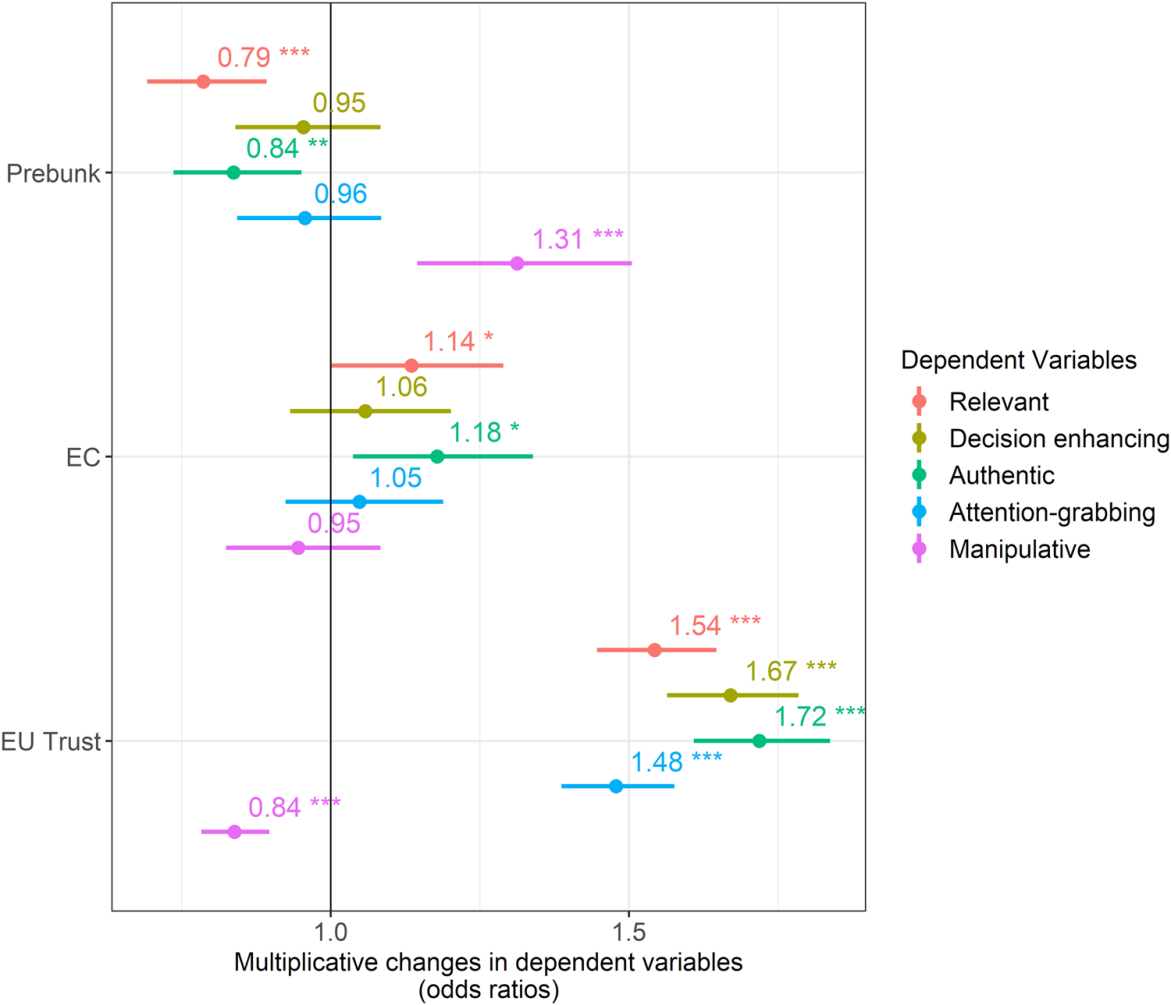
Effects of the intervention type, the intervention source and the level of trust in the EU on the perceptions of these interventions. Shows the estimates for the effects of the intervention (prebunk v debunk), the source (EC v neutral) and standardised trust in the EU (which is not a treatment variable) on the perceptions of the interventions with regard to being relevant, decision-enhancing, authentic, attention-grabbing and manipulative. Outcome variables are 1 if participants (strongly) agreed and 0 otherwise. Participants from the control condition are not included, as they saw no intervention that they could have rated. The EU trust level is standardised such that a 1-unit change corresponds to one standard deviation. The models are binary logistic regressions reporting the odds ratios and heteroscedasticity-robust 95% confidence intervals. Significance levels: *** *p* < 0.001, ** *p* < 0.01, * *p* < 0.05.

Several mechanisms have been put forward to explain why tailored interventions can be more or less effective than those that are not tailored^46^. To gain insights into these explanations, participants in our experiment evaluated the interventions on dimensions such as perceived relevance, usefulness for improving decision-making, authenticity, attention-grabbing nature and perceived manipulativeness. Ratings were recorded on 5-point Likert scales and subsequently dichotomised, with agreement and strong agreement coded as 1 and all other responses coded as 0. Binary logistic regression models were then used to estimate the impact of intervention type (prebunk v debunk), the provided source (EC v neutral) and level of trust in the EU as independent variables. Figure 5 presents the findings. The corresponding AMEs are shown in (Table S-9) in the supplementary material.

Our findings reveal three key points. First, prebunks are rated as less relevant, less authentic and more manipulative compared with debunks. Second, revealing that debunks and prebunks come from the European Commission makes participants tend to see them as (slightly) more relevant and authentic. Lastly, individuals with higher levels of trust in the EU are considerably more likely to perceive both interventions as relevant, decision-enhancing, authentic and attention-grabbing, while being less likely to view them as manipulative.

Further (exploratory) analyses incorporating interactions, similar to those discussed earlier, uncovered two notable cases of significant interaction effects between the European Commission source and the level of trust in the EU regarding perceptions of prebunks (see Figure S-17 in the supplementary material). In the first case, as the level of trust in the EU increases, presenting the Commission source becomes increasingly effective in enhancing people’s perception of the message’s usefulness for making informed decisions. In the second case, as the level of trust in the EU increases, the Commission source becomes less likely to be perceived as manipulative.

While these findings regarding perceptions of interventions do not entirely account for the effects observed in the main outcome variables, they do offer three insights. First, the lower effectiveness of prebunks compared with debunks for inducing expected behavioural changes may be attributed to their perceived lack of relevance and authenticity, coupled with a higher perception of manipulative intent. Importantly, these effects persist even when controlling for the source of each intervention. Second, although Commission-branded interventions are judged as less manipulative and more relevant, decision-enhancing, authentic and attention-grabbing, this does not translate into their higher effectiveness, as explored before. Third, the increased effectiveness of Commission debunks, in terms of reducing belief in and credibility assessment of false or misleading articles among individuals with high levels of trust in the EU, can be partially explained by the finding that as the EU trust level increases, the inclusion of the Commission source enhances the perception of the message as useful for making informed decisions and reduces its perceived manipulative nature.

## Discussion

First, our results demonstrate that debunking and prebunking interventions effectively address common misinformation claims related to COVID-19 and climate change in an EU context, notably in Germany, Greece, Ireland, and Poland, largely confirming previous knowledge primarily obtained from the United States. These interventions persistently influence three out of the four tested outcome variables in the expected direction. Notably, only the European Commission debunk and the neutral prebunk significantly increase levels of intention to share a misleading article to express disagreement.

Second, our findings indicate that debunks are slightly more effective than prebunks in combating misinformation. The two types of intervention do not differ in terms of reducing the perceived credibility of the misleading article claim and increasing people’s intention to share the article with others to express their disagreement. However, debunks do reduce beliefs in false claims and likelihood of intending to share the misleading articles to endorse them, more so than prebunks. This difference may be attributed to the fact that the employed debunks explicitly address the claims made in the misleading articles while also highlighting commonly used strategies, whereas prebunks solely focus on the latter. Although prebunks therefore have broader applicability, the fact that they do not specifically address the false claim that people are to encounter or provide a factual substitute may explain their lower effectiveness. Note that the design of our experiment does not allow a fully unbiased comparison between prebunks and debunks, nor does it capture the long-term benefits of prebunking against misinformation using the same misleading strategies^20,27^.

Perceptions of the debunking and prebunking interventions shed light on their effects on the main outcome variables, providing potential explanations. While a causal mediation analysis is not feasible in the current experimental design, the data suggest that prebunks are perceived as more manipulative (with 31.56 v 25.87% of respondents (strongly) agreeing that the intervention wanted to manipulate them) but less relevant and authentic than debunks, which may account for their reduced effectiveness. These findings indicate that the additional information in debunks, specifically addressing the content of the false claim, serves an important purpose.

Third, the findings show that, on average, revealing the source of the intervention (i.e. the European Commission in our experiment) has virtually no impact on the effectiveness of this intervention. This finding is both reassuring and disappointing for public institutions, policymakers and practitioners–reassuring, as it means that stamping an intervention with its government sponsor does not hurt the intervention overall, but disappointing, as one might hope that revealing that a government body is behind an intervention would increase its intended effect. We find that interventions from the EU are perceived as more relevant and authentic. Therefore, debunking and prebunking interventions remain robust and can be utilised by the EU as a mass-communication tool to counteract misinformation. That these findings could generalise to institutions like the World Health Organization, the Organisation for Economic Co-operation and Development or the United Nations seems plausible but needs to be verified empirically. Our results suggest that revealing the source of the intervention has no impact on its effectiveness. Whether this would also be the case for other sources is unsure.

Lastly, our analyses provide a first step towards disentangling the effects based on people’s trust in the source of the intervention. As a main effect, we find that trust in the EU is negatively correlated with belief in misleading articles, credibility assessment and intention to share the misleading article to agree with it, and positively with intention to share it to disagree. This aligns with existing evidence demonstrating a negative association between institutional trust and susceptibility to conspiracy theories and misinformation^47–50^. In terms of interaction effects, results show that, as the level of trust in the EU increases, European Commission debunks are more effective than neutral debunks for two out of the four outcome variables (i.e. agreement with the false claim and the perceived credibility of the false claim). Conversely, neutral debunking surpasses Commission debunking among individuals with low levels of trust in the EU only for the outcome variable of credibility assessment. The observed interaction effects cannot be fully explained by the available perception data. Although we do not find significant interactions between EU trust level and source for prebunks’ effectiveness, we do observe differences in how the intervention is perceived based on individuals’ levels of trust in the EU. Specifically, a higher level of trust in the EU is associated with lower perceptions of the intervention as manipulative and a higher perceived message usefulness, whereas a lower level of trust in the EU is linked to a lower perceived usefulness of EU interventions. We discuss important qualifications to these findings below.

We also note that some of the presented effects vary by country, although these analyses have not been preregistered and are thus exploratory, and we can only hypothesise about the causes of these differences. The slight country deviations do not seem to reflect any pattern explainable by dominant country characteristics, such as overall high/low levels of trust in the EU. Further investigation of potential explanations and demonstration of cross-country differences would help to better understand the generalisability of intervention effects and interactions with source information.

Based on our results, we recommend investing in trust-building measures alongside the design of interventions such as debunking and prebunking. As suggested by our results, trust alone can be a preventive factor against misinformation. Moreover, it is beneficial to identify population segments with high levels of mistrust and support interaction within those populations through direct peer-to-peer communication from trusted sources. Healthcare professionals, for instance, are typically perceived as trustworthy providers of health information^51–53^. Therefore, initiatives to enhance healthcare professionals’ skills in debunking vaccination misinformation during patient–professional interactions could complement the approaches employed in this study^54^.

Knowledge about institutional trust in different segments of the population could be further used by institutions to target and tailor prebunks and debunks. For example, selected groups could be addressed with more rigour and with explicitly designed prebunks and debunks—as opposed to a ‘one-size-fits-all’ or ‘shotgun’ approach where interventions address everyone in the same way. More specifically, in light of evidence that people with low levels of trust in public institutions are less receptive to some interventions against misinformation, these interventions could either be focused on more receptive segments of the population or be modified to make them more effective for those who are less receptive (or both). These processes correspond to what is known as targeting or tailoring, namely used in persuasion psychology^55,56^, and more specifically in health communication^46,57–61^, communication to reduce climate scepticism^62^, and recently also nudging^63,64^ and debunking^65^.

Targeting and tailoring interventions can enhance their effectiveness by matching specific features with recipient characteristics^46^. Tailored interventions recognise that individuals have different reasons for perceiving, liking, disliking or reacting to interventions, leading them to prioritise different dimensions of interventions^46^. These interventions can be more relevant, fitting, familiar, fluent, self-efficacy enhancing, authentic or attention-grabbing. However, tailored messages may also face challenges such as privacy concerns, perceived manipulation, unfair judgements, stereotyping or repetitiveness^46,66^. The Facebook–Cambridge analytica scandal serves as a cautionary example of misusing personal information for targeted campaigns. In 2018, a whistle-blower revealed that Cambridge analytica used personal information collected without the authorisation of the data subjects to profile and target them with personalised political advertisement^67^. Importantly, people were targeted based on their personality profiles, which were inferred from their likes—a practice which has been shown to work^68–70^. These targeted campaigns were said to have the objective to influence political preferences and thus elections—in particular the 2016 US presidential campaign and the Brexit referendum. Given the public’s negative perception of this event, the use of similar techniques for public policy requires critical assessment, meticulous planning and transparent implementation, particularly when used to respond to mis- and disinformation.

There are some caveats to our experiment that should be discussed to properly interpret our findings. First, the order in which we measured sharing intentions and beliefs about accuracy may have influenced participants’ decision-making^71^. Asking about beliefs beforehand could have prompted participants to consider accuracy, potentially reducing the likelihood of sharing misinformation. However, this could not possibly bias the treatment effects as the order was consistent across all groups. Second, despite our large representative sample, the external validity of our experiment is limited, as the interventions occurred immediately after exposure to misinformation, without any intermittent stimuli, potentially inflating effect sizes. It is unclear to what extent our findings generalise to more realistic situations, where the encounters with misinformation and corresponding prebunks and debunks are further apart in time, or to different designs of debunks and prebunks. Third, the slight advantage of debunks over prebunks that we observed for some outcome variables could be due to the debunks and prebunks being implemented at different points in time, with respect to encountering the misleading article, or due to the difference in content. We cannot unambiguously attribute this behavioural effect to one or the other. However, our findings align with previous findings attesting higher effectiveness to debunks compared with prebunks^27,28^, while contradicting findings of prebunks being more effective^29^. Fourth, participants’ self-reported levels of trust in the EU may be influenced by their assigned treatment, as they were elicited at the end of the experiment. Exposure to a European Commission debunk or prebunk could lead participants to evaluate the EU more favourably later on, potentially due to an experimenter demand effect. Indeed, our analysis shows slightly higher levels of trust among participants in the Commission source group, but the difference is not significant (*b* = 0.16, SE = 0.09, *p* = 0.09). In addition, participants in the neutral source (i.e. no revealed source of the intervention) conditions report significantly higher levels of trust in the EU compared with the control (i.e. no intervention) condition (*b* = 0.2, SE = 0.09, *p* = 0.04). This does not suggest the presence of an experimenter demand effect. Regressions indicate that individuals in the debunking condition report higher levels of trust in the EU than those in the control condition (*b* = 0.26, SE = 0.09, *p* = 0.005), whereas there is no significant difference for those in the prebunking condition. The moderation results may thus suffer from post-treatment bias and so must be approached with great caution^44^. However, we emphasise that the alternative approach—to elicit potential moderators before treatment—has its problems too, as such priming may lead to biased measurement of average treatment and interaction effects^72^. While priming bias is negligible in certain cases^73^ (average treatment moderation effects are not changed with an additional measurement of the moderator directly preceding treatment), this might not be the case for the moderators and treatments featured in our experiment, as measuring political identity (a type of measure similar to levels of trust in the EU) has been shown to induce priming bias in previous studies^74^. In any case, further investigations into the moderating role of trust in the EU with either a priori measurement or external manipulation are desirable to substantiate our findings and test their robustness. Fifth, we include two comprehension check questions before measuring dependent variables. This makes it possible that the comprehension checks impact responses to the main questions. Importantly, false responses to comprehension checks can lead participants to be exposed twice to the misinformation and/or the intervention. In the latter case, this could lead to inflated treatment effect estimates. We observe that being exposed to the intervention twice predicts the dependent variables in a way that does not correspond to the desired effect of the interventions, if it predicts them at all. We interpret this evidence as ininconsistent with inflated treatment effect estimates, yet insufficient to exclude this possibility. Lastly, as we investigate the effect of prebunks and debunks and cannot credibly debunk true statements, we opted to only include misinformation in our set of stimuli, prohibiting us from measuring truth discernment^75^. Thus, we acknowledge the concern that debunking may increase general scepticism (as has been shown for game-based inoculation^76,77^) and that decreases in belief may not be specific to the claim that is being assessed. While we attempt to capture the possibility of a ‘chilling effect’ of the interventions by also measuring the effect on intention to share misinformation to disagree with it, it does not eliminate this concern.

In conclusion, this study highlights the effectiveness of debunking and prebunking interventions in combating misinformation about COVID-19 vaccination and climate change in EU Member States. Institutions with the necessary resources, like the European Commission, should prioritise investing in these interventions, potentially targeted or tailored, due to the lack of evidence suggesting the prevalence of unintended effects, but pending further scientific evidence on this issue.

## Methods

### Participants

This study was run in October 2022 with a total of N = 5 228 participants who completed the experiment (Germany: *n* = 1 311; Greece: *n* = 1 313; Ireland: n = 1, 296; Poland: *n* = 1 308). All participants who finished the survey were included in the analyses, consistent with the preregistration. Participants took on average 19.59 min (SD = 132.31, median = 10.85) to complete the experiment. Ipsos NV paid participants a flat fee of around 220 panel points, which corresponds to around EUR 2.20. Points can be redeemed in the Ipsos Giftshop, where panellists have access to different products depending on the number of points.

To select the countries involved in this study, we loosely categorised all Member States according to whether a relatively large or small fraction of respondents indicated that they encountered fake news almost every day or once per week and according to the fraction of respondents indicating that they were confident or not confident in their ability to identify fake news, based on data from the Flash Eurobarometer 464 on fake news and disinformation online^78^. In line with the market access of the panel company, we selected countries with variation regarding these two variables and assuring appropriate geographical diversity. The data underlying the country selection are provided in (Table S-25) in the supplementary material.

The sampling process involved quotas based on age, gender and geographical region (NUTS (nomenclature of territorial units for statistics) regions) to ensure a representative sample of each country’s population. Among the respondents, 52.22% identified as female, 46.89% identified as male and the remaining respondents chose neither of those. The age distribution was as follows: 9.7% were between 18 and 24, 15.61% between 25 and 34, 18.1% between 35 and 44, 18.06% between 45 and 54, 25.84% between 55 and 64, and 12.69% above 65 years old. For detailed regional spread and sample characteristics by country, refer to (Table S-17–Table S-21) in the supplementary material.

### Power analysis

A power analysis was conducted using data from a pilot experiment consisting of 875 observations (more information on the pilot experiment is provided below). This calculation assumed a 5% significance threshold and a two-tailed *z*-test from a logistic regression. The required number of observations is 1,300 participants from each country.

### Preregistration and ethical approval

The preregistration is available at https://aspredicted.org/5hk7s.pdf. The experiment was reviewed and cleared by the Ethics Committee of the Faculty of Economic Sciences, University of Warsaw, and by the Joint Research Centre Research Ethics Board, both confirming that all methods were carried out in accordance with relevant guidelines and regulations and approving all experimental protocols. Informed consent was obtained from all subjects according to data protection Regulation (EU) 2018/1725 for EU institutions, bodies and agencies.

### Exclusion criteria

Following the preregistration, only participants who did not complete the full survey were excluded from the dataset. A total of *n* = 5666 observations were removed, which includes participants who were screened out due to quota requirements. Among the exclusions, at least *n* = 2305 participants (21.16%) voluntarily dropped out, while *n* = 3361 individuals did not proceed beyond the screening stage. Although we cannot distinguish between participants screened out by us and those who dropped out voluntarily at the screening stage, our experiment monitoring indicates that the majority were screened out due to quota requirements. This does not suggest a problem in dropouts during the experiment.

### Recruitment and experimental treatments

The experiment was conducted online using LimeSurvey. Participants were recruited and paid a fixed amount by online panel provider Ipsos NV. Participants were randomly assigned to one of five treatment groups. Participants read a prebunking message (prebunk), a debunking message (debunk) or no message (control). Both the prebunk and debunk messages were further subdivided according to the information on the source responsible for their implementation. There was either no information (no source) or information that the European Commission implemented the intervention (EC-source). Thus, the design was a 2 (intervention: prebunk v debunk) × 2 (intervention source: no source v European Commission) + 1 (control) between-subjects design. Furthermore, we introduced between-subjects variation regarding the topic of misinformation and the specific misleading article. The misinformation topics (and related interventions) concerned 2 major topics (climate change and COVID-19), for each of which there were 3 misinformation claims. Both the topic and misinformation claim factors serve as robustness checks rather than treatment factors. Consequently, our main analyses pool across these topics and claims to ensure sufficient power for the main hypothesis tests (however, see the supplementary material for a discussion of differences by content).

### Experimental materials

The supplementary material (‘Experimental materials’) contains the texts used for the interventions and misleading articles, along with examples of how they were presented. The prebunks and debunks were designed to be highly similar, which allowed us to compare the effectiveness of the interventions. More precisely, debunks included all the information from prebunks and additional details specific to the misinformation addressed. Debunks informed those who had encountered specific misinformation after the fact, whereas prebunks were more general and preceded encounters with misinformation. For this reason, whereas debunks included a statement, prebunks centred on a question (‘Is this misinformation?’). It can also be noted that the prebunks, compared with the debunks, placed more focus on sharing. This is ecologically valid given the prevalence of ‘check before sharing’, ‘think before you share’ and similar prebunking campaigns. Still, it is a potential concern that this difference could have an undue effect on the participants: we could expect that prebunks, compared with analogous debunks, would do particularly well in terms of discouraging sharing (compared with other effects). This is not what we observe–prebunks only do as well as debunks in terms of lower credibility assessment, not in terms of reduced willingness to share.

With regard to the misleading articles, each presented one of six claims: three about climate change and three about COVID-19. These six claims were selected from a set of 17 claims: eight on COVID-19 and nine on climate change (more on how these were selected below). Apart from their specific claims and pictures, the misleading articles were identical. To create the articles, a misinformation claim was combined with a catchy headline, picture and teaser text. Common misinformation techniques were employed in the generic text, and the article was edited to resemble a typical online news item, including a blurred date and author information. The articles used common misinformation techniques to enhance their credibility, including appeals to emotions, morality and claims of absolute truth. They also employed strategies to undermine contrary claims, such as questioning the credibility and morality of experts, or alleging the existence of a conspiracy.

The selection of three COVID-19 and three climate change claims involved two pretests (different from the pilot described below). The first pretest, conducted in May 2022 in Germany, Greece and Poland, had 301 participants rate a random set of four (out of eight possible) candidate claims about COVID-19. The second pretest took place in September 2022 in Germany, Greece, Ireland, and Poland, with 416 participants rating a random selection of four (out of nine possible) claims about climate change. The original set of 17 claims was sourced from real claims found online (climate change, Skeptical Science Website under https://skepticalscience.com/argument.php; COVID-19, ESOC COVID-19 Misinformation Dataset under https://esoc.princeton.edu/publications/esoc-covid-19-misinformation-dataset). Participants rated each misleading claim’s credibility, indicated their intention to share it or not and, if they wanted to share it, their reason for doing so (more detail on the outcome variables is provided below). The three claims in each category that were used for articles in the study were selected by ranking all claims from highest to lowest for each outcome variable separately and then counting the number of times each claim had been in the top three for each outcome variable. The six final claims that were selected for presentation in articles in the main experiment were (1) the planet hasn’t warmed since 1998; (2) there is no scientific consensus on climate change; (3) climate models are unreliable; (4) the COVID-19 vaccine does not work; (5) the COVID-19 vaccine has not been properly tested in clinical trials; and (6) the COVID-19 vaccine is dangerous. Examples of the articles used are provided in the supplementary material (‘Experimental materials’). Participants in pretests did not participate in the main experiment.

### Experimental procedure

After reading an introduction to and explanation of the experiment, participants followed a specific sequence based on their assigned intervention treatment. In the prebunk condition, participants received the prebunking message before reading the misleading article. In the debunk condition, participants read the debunking message after reading the misleading article. The control condition involved participants only reading the article (for the specific sequence and elements contained therein, see Table 1).

**Table 1:** Experimental sequence for different treatments.

Treatment	Introduction	Prebunk	Misinformation	Debunk	DVs	Questionnaire	Debriefing
Control	Yes	No	Yes	No	Yes	Yes	Yes
Prebunk	Yes	Yes	Yes	No	Yes	Yes	Yes
Debunk	Yes	No	Yes	Yes	Yes	Yes	Yes

NB: Indicates whether a specific component of the experiment (column) occurs in the respective intervention treatment (rows).

*DV* dependent variable.

After receiving the intervention and reading the misleading article, participants answered three groups of questions in the following order: first, participants stated their belief in the respective misinformation claim on a 5-point Likert scale ranging from ‘strongly disagree’ to ‘strongly agree’. For example, participants who had read the misleading article claiming that “it hasn’t warmed since 1998 indicated if they agreed with this statement or not. Second, participants reported their intention to share the misinformation article or not and, conditional on their response, gave the reasons for their intention. If they did intend to share it, they indicated their intention to (a) share the article online with people who were close to them; (b) share the article online and publicly; (c) talk face to face about the article with people who were close to them; and (d) talk face to face about the article publicly. Participants indicated their agreement on 5-point Likert scales with the options ‘not at all’, ‘a little’, ‘neither a little nor a lot’, ‘much’ and ‘very much’. Respondents who chose anything other than ‘not at all’ at least once were asked about their reason for wanting to share the article. Participants provided their response on a 5-point Likert scale ranging from ‘to express that I totally disagree with it’ to ‘to express that I totally agree with it’. Third, participants indicated their perceptions of the credibility of the article. Specifically, they assessed credibility on four dimensions, using 5-point semantic differential scales^79^. These dimensions assessed credibility with respect to accuracy (‘inaccurate’ to ‘accurate’), believability (‘unbelievable’ to ‘believable’), factuality (‘opinionated’ to ‘factual’) and trustworthiness (‘untrustworthy’ to ‘trustworthy’). For the analyses, these individual dimensions were combined into a credibility assessment variable. These questions were forced choice, with no ‘I don’t know’ or ‘Do not want to say’ options.

After responding to these questions, participants entered a post-experimental questionnaire. Most importantly, they reported their levels of trust in the EU. Specifically, they answered the question ‘How much trust do you have in the European Union?’ on a 10-point scale ranging from ‘I do not trust it at all’ to ‘I trust it completely’. The other questions related to trust in the national government of the respondent, general trust, agreement with EU-specific statements, perceptions of the prebunk or debunk, the perceived source of the prebunk or debunk and further general questions related to perceptions of misinformation. After the questionnaire, all participants were debriefed. See the supplementary material for the questionnaire and debriefing message.

### Comprehension checks

Participants were presented with two comprehension check questions, one after being exposed to the prebunk or debunk and another after reading the misleading article. These questions assessed understanding of the intervention and the misinformation. If participants answered a question incorrectly or left it unanswered, they were instructed to review the corresponding text (prebunk, debunk, misinformation) before proceeding. To check for comprehension of the misinformation article, after reading it, participants in all treatments were asked ‘Does the article claim that the vaccine is safe?’ if the article was on COVID-19 or ‘Does the article claim that climate change is happening?’ if the article was on climate change (correct answer: no). Participants in the prebunk treatment were asked, after reading the prebunk, ‘Does the previous article state that an often-used technique to mislead people is to claim that there is a malevolent actor behind everything?’ (correct answer: yes). Participants in the debunk treatment were asked, after reading the debunk, ‘Does the article claim that it is a myth that [relevant claim from the article]?’ (correct answer: yes). Participants in the control treatment, who saw an unrelated article on financial decision-making, were asked ‘Does the previous article argue that financial decisions can be quite complex?’ (correct answer: yes).

### Pilot experiment

The pilot experiment was conducted in May 2022, with a total of *n* = 875 participants completing it (Germany: *n* = 293; Greece: *n* = 282; Poland: *n* = 300). All observations were included in the analysis. Participants were sampled based on quotas to ensure a sample representative of each country’s public, considering age, gender and geographical region (NUTS regions). The participant breakdown was 51.31% female and 48.69% male, with age distributed as follows: 29.87% were between 18 and 34, 22.75% between 35 and 44, and 46.79% between 45 and 64 years old (0.58% did not provide a response). The pilot aimed to test the initial design, identify potential improvements and generate initial estimates for effect sizes to inform power analyses for the main experiment. It led to changes in the experiment’s sequencing and the inclusion of a control group. The pilot experiment focused on debunks of COVID-19 misinformation.

### Analysis

The four main variables were analysed according to the preregistration as follows: agreement with the claim was analysed using an ordered logit model with the ordered response variable. Credibility assessments were analysed using an ordinary least squares model, summing the four credibility responses as the dependent variable. Behavioural intentions were analysed using two binary logistic models, dichotomising the ordered variable to represent whether respondents expressed an intention to circulate the misleading article and indicated doing so to express (dis-)agreement or total (dis-)agreement, zero otherwise.

For all main hypothesis tests (i.e. the interaction effects), the independent variables included the intervention source, the metric EU trust variable and their interaction. Heteroskedasticity-robust standard errors were used for all model estimations.

### Robustness checks

We conducted robustness checks for key analyses, as preregistered. These checks control for age, gender, level of education, country of residence, political ideology, trust in the national government, general trust, a trust index in the EU, need for cognition, frequency of social media use, perceived frequency of encountering misinformation, perceived importance of sharing true information and confidence in identifying misinformation, as well as responses to the comprehension check questions and a manipulation check regarding the correct identification of the intervention source. Analyses incorporating the misinformation topic are conducted separately in the supplementary material.

## Supplementary Information

Supplementary Information.

## Data Availability

The data supporting the findings of this study are available from the Open Science Framework (https://osf.io/7kytz/?view_only=4ea3191090fd4eb08938ce4979ab296f, doi: 10.17605/OSF.IO/7KYTZ).

## Code availability

The syntax used to analyse the dataset in this study is available from the Open Science Framework (https://osf.io/7kytz/?view_only=4ea3191090fd4eb08938ce4979ab296f, doi: 10.17605/OSF.IO/7KYTZ).

## References

[1] Treen d’I, K. M., Williams, H. T. P. & O’Neill, S. J. Online misinformation about climate change. *WIREs Clim. Ch.* **11**, e665 (2020). 10.1002/wcc.665

[2] Bruns, H., Dessart, F. J. & Pantazi, M. *Covid-19 misinformation: Preparing for future crises*, EUR 31139 EN, Publications Office of the European Union, Luxembourg, ISBN 978-92-76-54519-4, JRC130111. 10.2760/41905 (2022).

[3] Pummerer, L. *et al.* Conspiracy theories and their societal effects during the COVID-19 pandemic. *Soc. Psychol. Personal. Sci.* **13**, 49–59 (2022). 10.1177/19485506211000217

[4] Imhoff, R. & Lamberty, P. A bioweapon or a hoax? The link between distinct conspiracy beliefs about the Coronavirus disease (COVID-19) outbreak and pandemic behavior. *Soc. Psychol. Personal. Sci.* **11**, 1110–1118 (2020). 38602949 10.1177/1948550620934692 PMC7342934

[5] Loomba, S., Figueiredo, A., Piatek, S. J., Graaf, K. & Larson, H. J. Measuring the impact of COVID-19 vaccine misinformation on vaccination intent in the UK and USA. *Nat. Hum. Behav.* **5**, 337–348 (2021). 33547453 10.1038/s41562-021-01056-1

[6] Bursztyn, L., Rao, A., Roth, C. & Yanagizawa-Drott, D. Opinions as facts. *Rev. Econ. Stud.* **90**, 1–33 (2022).

[7] van der Linden, S. The conspiracy-effect: Exposure to conspiracy theories (about global warming) decreases pro-social behavior and science acceptance. *Personal. Individ. Differ.* **87**, 171–173 (2015). 10.1016/j.paid.2015.07.045

[8] Lewandowsky, S., Ecker, U. K. H. & Cook, J. Beyond Misinformation: understanding and coping with the “post-truth” era. *J. Appl. Res. Mem. Cogn.* **6**, 353–369 (2017). 10.1016/j.jarmac.2017.07.008

[9] Ecker, U. K. H. *et al.* The psychological drivers of misinformation belief and its resistance to correction. *Nat. Rev. Psychol.* **1**, 13–29 (2022). 10.1038/s44159-021-00006-y

[10] Lewandowsky, S., Cook, J. & Lombardi, D. Debunking handbook 2020. *Databrary*10.17910/b7.1182 (2020). 10.17910/b7.1182

[11] Chan, M. P. S., Jones, C. R., Hall Jamieson, K. & Albarracín, D. Debunking: A meta-analysis of the psychological efficacy of messages countering misinformation. *Psychol. Sci.* **28**, 1531–1546 (2017). 28895452 10.1177/0956797617714579 PMC5673564

[12] Lewandowsky, S., Ecker, U. K. H., Seifert, C. M., Schwarz, N. & Cook, J. Misinformation and its correction: Continued Influence and successful debiasing. *Psychol. Sci. Pub. Interest* **13**, 106–131 (2012). 26173286 10.1177/1529100612451018

[13] Cook, J., Lewandowsky, S. & Ecker, U. K. H. Neutralizing misinformation through inoculation: Exposing misleading argumentation techniques reduces their influence. *PLoS ONE* **12**, e0175799 (2017). 28475576 10.1371/journal.pone.0175799 PMC5419564

[14] van der Linden, S., Leiserowitz, A., Rosenthal, S. & Maibach, E. Inoculating the public against misinformation about climate change. *Glob. Chall.* **1**, 1600008 (2017). 31565263 10.1002/gch2.201600008 PMC6607159

[15] Lewandowsky, S. & van der Linden, S. Countering misinformation and fake news through inoculation and prebunking. *Eur. Rev. Soc. Psychol.* **32**, 348–384 (2021). 10.1080/10463283.2021.1876983

[16] Traberg, C. S., Roozenbeek, J. & van der Linden, S. Psychological inoculation against misinformation: Current evidence and future directions. *Ann. Am. Acad. Pol. Soc. Sci.* **700**, 136–151 (2022). 10.1177/00027162221087936

[17] van der Linden, S., Maibach, E., Cook, J., Leiserowitz, A. & Lewandowsky, S. Inoculating against misinformation. *Science* **358**, 1141–1142 (2017). 29191898 10.1126/science.aar4533

[18] Basol, M., Roozenbeek, J. & van der Linden, S. Good news about bad news: Gamified inoculation boosts confidence and cognitive immunity against fake news. *J. Cogn.* **3**, 2 (2020). 31934684 10.5334/joc.91 PMC6952868

[19] Basol, M. *et al.* Towards psychological herd immunity: Cross-cultural evidence for two prebunking interventions against COVID-19 misinformation. *Big Data Soc.* **8**, 205395172110138 (2021). 10.1177/20539517211013868

[20] Maertens, R., Roozenbeek, J., Basol, M. & van der Linden, S. Long-term effectiveness of inoculation against misinformation: Three longitudinal experiments. *J. Exp. Psychol. Appl.* **27**, 1–16 (2021). 33017160 10.1037/xap0000315

[21] Roozenbeek, J., van der Linden, S., Goldberg, B., Rathje, S. & Lewandowsky, S. Psychological inoculation improves resilience against misinformation on social media. *Sci. Adv.*10.1126/sciadv.abo6254 (2022). 36001675 10.1126/sciadv.abo6254 PMC9401631

[22] Roozenbeek, J. & van der Linden, S. Fake news game confers psychological resistance against online misinformation. *Palgrave Commun.*10.1057/s41599-019-0279-9 (2019). 10.1057/s41599-019-0279-9

[23] Walter, N. & Murphy, S. T. How to unring the bell: A meta-analytic approach to correction of misinformation. *Commun. Monogr.* **85**, 423–441 (2018). 10.1080/03637751.2018.1467564

[24] Walter, N., Brooks, J. J., Saucier, C. J. & Suresh, S. Evaluating the impact of attempts to correct health misinformation on social media. *Health Commun.* **36**, 1776–1784 (2020). 32762260 10.1080/10410236.2020.1794553

[25] Porter, E. & Wood, T. J. The global effectiveness of fact-checking: Evidence from simultaneous experiments in Argentina, Nigeria, South Africa, and the United Kingdom. *Proc. Natl. Acad. Sci. USA* **118**, e2104235118 (2021). 34507996 10.1073/pnas.2104235118 PMC8449384

[26] Vivion, M. *et al.* Prebunking messaging to inoculate against COVID-19 vaccine misinformation: An effective strategy for public health. *J. Commun. Healthc.* **15**, 232–242 (2022). 10.1080/17538068.2022.2044606

[27] Tay, L. Q., Hurlstone, M. J., Kurz, T. & Ecker, U. K. H. A comparison of prebunking and debunking interventions for implied versus explicit misinformation. *Br. J. Psychol.* **113**, 591–607 (2022). 34967004 10.1111/bjop.12551

[28] Brashier, N. M., Pennycook, G., Berinsky, A. J. & Rand, D. G. Timing matters when correcting fake news. *Proc. Natl. Acad. Sci. USA* **118**, e2020043118 (2021). 33495336 10.1073/pnas.2020043118 PMC7865139

[29] Jolley, D. & Douglas, K. M. Prevention is better than cure: Addressing anti-vaccine conspiracy theories. *J. Appl. Soc. Psychol.* **47**, 459–469 (2017). 10.1111/jasp.12453

[30] Petty, R. E. & Cacioppo, J. T. The elaboration likelihood model of persuasion. *Adv. Exp. Soc. Psychol.* **19**, 123–205 (1986). 10.1016/S0065-2601(08)60214-2

[31] Pornpitakpan, C. The persuasiveness of source credibility: A critical review of five decades’ evidence. *J. Appl. Soc. Pyschol.* **34**, 243–281 (2004). 10.1111/j.1559-1816.2004.tb02547.x

[32] Pennycook, G. & Rand, D. G. The psychology of fake news. *Trends Cogn. Sci.* **25**, 388–402 (2021). 33736957 10.1016/j.tics.2021.02.007

[33] Walter, N. & Tukachinsky, R. A meta-analytic examination of the continued influence of misinformation in the face of correction: How powerful is it, why does it happen, and how to stop it?. *Commun. Res.* **47**, 155–177 (2020). 10.1177/0093650219854600

[34] Guillory, J. J. & Geraci, L. Correcting erroneous inferences in memory: The role of source credibility. *J. Appl. Res. Mem. Cogn.* **2**, 201–209 (2013). 10.1016/j.jarmac.2013.10.001

[35] Vraga, E. K. & Bode, L. I do not believe you: How providing a source corrects health misperceptions across social media platforms. *Inform. Commun. Soc.* **21**, 1337–1353 (2018). 10.1080/1369118X.2017.1313883

[36] Ecker, U. K. H. & Antonio, L. M. Can you believe it? An investigation into the impact of retraction source credibility on the continued influence effect. *Mem. Cogn.* **49**, 631–644 (2021). 10.3758/s13421-020-01129-y PMC7810102 33452666

[37] Seo, H., Xiong, A., Lee, S. & Lee, D. If you have a reliable source, say something: Effects of correction comments on COVID-19 misinformation. *ICWSM* **16**, 896–907 (2022). 10.1609/icwsm.v16i1.19344

[38] European Commission. Tackling coronavirus disinformation. https://commission.europa.eu/strategy-and-policy/coronavirus-response/fighting-disinformation/tackling-coronavirus-disinformation_en (2021).

[39] Tappin, B. M., Wittenberg, C., Hewitt, L. B., Berinsky, A. J. & Rand, D. G. Quantifying the potential persuasive returns to political microtargeting. *Proc. Natl. Acad. Sci. USA* **120**, e2216261120 (2023). 37307486 10.1073/pnas.2216261120 PMC10288628

[40] Carey, J. M. *et al.* The ephemeral effects of fact-checks on COVID-19 misperceptions in the United States, great Britain and Canada. *Nat. Hum. Behav.* **6**, 236–243 (2022). 35115678 10.1038/s41562-021-01278-3

[41] Harjani, T., Basol, M.-S., Roozenbeek, J. & van der Linden, S. Gamified inoculation against misinformation in India: A randomized control trial. *JOTE*10.36850/e12 (2023). 10.36850/e12

[42] Spampatti, T., Hahnel, U. J. J., Trutnevyte, E. & Brosch, T. Psychological inoculation strategies to fight climate disinformation across 12 countries. *Nat. Hum. Behav.*10.1038/s41562-023-01736-0 (2023). 38036655 10.1038/s41562-023-01736-0 PMC10896732

[43] Offer-Westort, M., Rosenzweig, L. R. & Athey, S. Battling the coronavirus ‘infodemic’ among social media users in Kenya and Nigeria. *Nat. Hum. Behav.*10.1038/s41562-023-01810-7 (2024). 38499773 10.1038/s41562-023-01810-7

[44] Montgomery, J. M., Nyhan, B. & Torres, M. How conditioning on posttreatment variables can ruin your experiment and what to do about it. *Am. J. Polit. Sci.* **62**, 760–775 (2018). 10.1111/ajps.12357

[45] Esarey, J. & Sumner, J. L. Marginal effects in interaction models: Determining and controlling the false positive rate. *Comparative Polit. Stud.* **51**, 1144–1176 (2018). 10.1177/0010414017730080

[46] Teeny, J. D., Siev, J. J., Briñol, P. & Petty, R. E. A review and conceptual framework for understanding personalized matching effects in persuasion. *J Consum. Psychol.* **31**, 382–414 (2021). 10.1002/jcpy.1198

[47] Roozenbeek, J. *et al.* Susceptibility to misinformation about COVID-19 around the world. *R. Soc. Open Sci.* **7**, 201199 (2020). 33204475 10.1098/rsos.201199 PMC7657933

[48] Eberl, J. M., Huber, R. A. & Greussing, E. From populism to the ‘plandemic’: Why populists believe in COVID-19 conspiracies. *J. Elect. Pub. Opin. Parties* **31**, 272–284 (2021). 10.1080/17457289.2021.1924730

[49] Pickles, K. *et al.* COVID-19 misinformation trends in australia: prospective longitudinal national survey. *J. Med. Internet Res.* **23**, e23805 (2021). 33302250 10.2196/23805 PMC7800906

[50] Šrol, J., Ballová Mikušková, E. & Čavojová, V. When we are worried, what are we thinking? Anxiety, lack of control, and conspiracy beliefs amidst the COVID-19 pandemic. *Appl. Cogn. Psychol.* **35**, 720–729 (2021). 33821088 10.1002/acp.3798 PMC8013184

[51] Vraga, E. K. & Bode, L. Using expert sources to correct health misinformation in social media. *Sci. Commun.* **39**, 621–645 (2017). 10.1177/1075547017731776

[52] van der Meer, T. G. L. A. & Jin, Y. Seeking formula for misinformation treatment in public health crises: The effects of corrective information type and source. *Health Commun.* **35**, 560–575 (2020). 30761917 10.1080/10410236.2019.1573295

[53] Durantini, M. R., Albarracín, D., Mitchell, A. L., Earl, A. N. & Gillette, J. C. Conceptualizing the influence of social agents of behavior change: A meta-analysis of the effectiveness of HIV-prevention interventionists for different groups. *Psychol. Bull.* **132**, 212–248 (2006). 16536642 10.1037/0033-2909.132.2.212 PMC4803282

[54] Baggio, M., Krawczyk, M., Nohlen, H., Pantazi, M. & Proestakis, A. Applying lessons from behavioural sciences to vaccination acceptance and demand. Publications Office of the European Union, Luxembourg, JRC131583. 10.2760/420194 (2022).

[55] Luong, K. T., Garrett, R. K. & Slater, M. D. Promoting persuasion with ideologically tailored science messages: A novel approach to research on emphasis framing. *Sci. Commun.* **41**, 488–515 (2019). 10.1177/1075547019862559

[56] Joyal-Desmarais, K., Rothman, A. J. & Snyder, M. How do we optimize message matching interventions? Identifying matching thresholds, and simultaneously matching to multiple characteristics. *Eur. J. Soc. Psychol.* **50**, 701–720 (2020). 10.1002/ejsp.2645

[57] Noar, S. M., Benac, C. N. & Harris, M. S. Does tailoring matter? Meta-analytic review of tailored print health behavior change interventions. *Psychol. Bull.* **133**, 673–693 (2007). 17592961 10.1037/0033-2909.133.4.673

[58] Schmid, K. L., Rivers, S. E., Latimer, A. E. & Salovey, P. Targeting or tailoring?. *Mark. Health Serv.* **28**, 32–37 (2008). 18389854 PMC2728473

[59] Pink, S. L., Chu, J., Druckman, J. N., Rand, D. G. & Willer, R. Elite party cues increase vaccination intentions among republicans. *Proc. Natl. Acad. Sci.* **118**, e2106559118 (2021). 34312254 10.1073/pnas.2106559118 PMC8364165

[60] Mäki, K. O. *et al.* Tailoring interventions to suit self-reported format preference does not decrease vaccine hesitancy. *PLoS ONE* **18**, e0283030 (2023). 36943860 10.1371/journal.pone.0283030 PMC10030039

[61] Habib, G. L. *et al.* The importance of cultural tailoring of communicators and media outlets in an influenza vaccination awareness campaign: A digital randomized trial. *Sci. Rep.* **13**, 1744 (2023). 36797274 10.1038/s41598-023-27910-y PMC9935604

[62] Dixon, G., Hmielowski, J. & Ma, Y. Improving climate change acceptance among U.S. conservatives through value-based message targeting. *Sci. Commun.* **39**, 520–534 (2017). 10.1177/1075547017715473

[63] Mills, S. Personalized nudging. *Behav. Pub. Policy*10.1017/bpp.2020.7 (2020). 10.1017/bpp.2020.7

[64] Peer, E. *et al.* Nudge me right: Personalizing online nudges to people’s decision-making styles. *Comput. Hum. Behav.* **109**, 106347 (2020). 10.1016/j.chb.2020.106347

[65] Lunz Trujillo, K., Motta, M., Callaghan, T. & Sylvester, S. Correcting misperceptions about the MMR vaccine: Using psychological risk factors to inform targeted communication strategies. *Polit. Res. Quart.* **74**, 464–478 (2021). 10.1177/1065912920907695

[66] Kozyreva, A., Lorenz-Spreen, P., Hertwig, R., Lewandowsky, S. & Herzog, S. M. Public attitudes towards algorithmic personalization and use of personal data online: Evidence from Germany, great Britain, and the United States. *Humanit. Soc. Sci. Commun.* **8**, 117 (2021). 10.1057/s41599-021-00787-w

[67] Cadwalladr, C. & Graham-Harrison, E. Revealed: 50 million Facebook profiles harvested for Cambridge analytica in major data breach. *Guardian* **17**, 22 (2018).

[68] Matz, S. C., Kosinski, M., Nave, G. & Stillwell, D. J. Psychological targeting as an effective approach to digital mass persuasion. *Proc. Natl. Acad. Sci.* **114**, 12714–12719 (2017). 29133409 10.1073/pnas.1710966114 PMC5715760

[69] Kosinski, M., Stillwell, D. & Graepel, T. Private traits and attributes are predictable from digital records of human behavior. *Proc. Natl. Acad. Sci. USA* **110**, 5802–5805 (2013). 23479631 10.1073/pnas.1218772110 PMC3625324

[70] Walker, C., O’Neill, S. & De-Wit, L. Evidence of psychological targeting but not psychological tailoring in political persuasion around brexit. *Exp. Results* **1**, e38 (2020). 10.1017/exp.2020.43

[71] Pennycook, G., Binnendyk, J., Newton, C. & Rand, D. G. A practical guide to doing behavioral research on fake news and misinformation. *Collabra Psychol.* **7**, 25293 (2021). 10.1525/collabra.25293

[72] Blackwell, M. et al. Priming bias versus post-treatment bias in experimental designs. Preprint at http://arxiv.org/abs/2306.01211 (2024).

[73] Sheagley, G. & Clifford, S. No evidence that measuring moderators alters treatment effects. *Am. J. Polit. Sci.*10.1111/ajps.12814 (2023). 10.1111/ajps.12814

[74] Morris, M. W., Carranza, E. & Fox, C. R. Mistaken identity: Activating conservative political identities induces “conservative” financial decisions. *Psychol. Sci.* **19**, 1154–1160 (2008). 19076488 10.1111/j.1467-9280.2008.02217.x

[75] Guay, B., Berinsky, A. J., Pennycook, G. & Rand, D. How to think about whether misinformation interventions work. *Nat. Hum. Behav.* **7**, 1231–1233 (2023). 37563304 10.1038/s41562-023-01667-w

[76] Modirrousta-Galian, A. & Higham, P. A. Gamified inoculation interventions do not improve discrimination between true and fake news: Reanalyzing existing research with receiver operating characteristic analysis. *J. Exp. Psychol. Gen.* **152**, 2411–2437 (2023). 36996156 10.1037/xge0001395

[77] Pennycook, G. et al. Misinformation inoculations must be boosted by accuracy prompts to improve judgments of truth. (PsyArXiv Preprints, 2023) 10.31234/osf.io/5a9xq.

[78] European Commission. *Flash Eurobarometer 464* (Publications Office of the European Union, 2018).

[79] Gaziano, C. & McGrath, K. Measuring the concept of credibility. *Journal. Quart.* **63**, 451–462 (1986). 10.1177/107769908606300301

