# Supplementary material for: Investigating the role of source and source trust in prebunks and debunks of misinformation in online experiments across four EU countries
Source: Sci Rep. 2024 Sep 5;14:20723. doi: 10.1038/s41598-024-71599-6 (PMC11377563; doi:10.1038/s41598-024-71599-6)
Supplement: Supplementary file 1 — Supplementary Information. [file 41598_2024_71599_MOESM1_ESM.docx]

# Supplementary material

## Descriptive analyses

This section describes the main dependent variables. Figure S-6 shows that most participants disagreed with the false claim they encountered, but a notable fraction (25.54 %) agreed or strongly agreed with it. A quarter of the respondents (25.82 %) considered the misinformation completely non-credible across all dimensions (i.e. they rated them as inaccurate, unbelievable, opinionated and untrustworthy). A significant portion (31.83 %) chose not to share or discuss the misleading article, while 26.84 % wanted to express (strong) disagreement and 18.36 % wanted to signal (strong) agreement. In the main analyses, we do not differentiate sharing intentions according to the intended target group (‘people close to you’ or ‘publicly’) or the type of sharing (‘face to face’ or ‘online’). The distributions of these intentions are shown in Figure S-7. There were significant associations between all main dependent variables (see Table S-2 for Spearman rank correlations). Notably, agreement with the main claim, credibility assessment and intention to agree were substantially positively correlated. Intention to disagree as a reason to share the misleading article were weakly negatively correlated with the other variables.

Table S-3 presents mean values of the main dependent variables, both by treatment and in aggregate, along with the *p*-values of non-parametric Kruskal–Wallis tests. The distributions of these variables differed by treatment. Each intervention led to a decrease in agreement with the main claim, in the credibility assessment of the misleading article and in the cumulative credibility ratings. Likewise, the proportions of participants intending to share or discuss the misleading article to (strongly) agree with its main claim were lower in the treatment groups. Conversely, the proportions of individuals planning to share or talk about the misleading article to (strongly) disagree with it were slightly higher among the groups receiving an intervention.

Figure S-8 displays the distribution of the level of trust in the EU, which is the main moderator of interest for the main analyses. On average, the trust level is 5.46 (SD = 2.5). The variable is not normally distributed (Kolmogorov–Smirnov test: *d* = 0.87, *p* < 0.001), with many observations (around 10 %) corresponding to a minimal level of trust in the EU. Trust levels differed significantly among the four countries (*p* < 0.000 in a Kruskal–Wallis test), with Greece having the lowest values and Ireland having the highest (see Figure S-9).


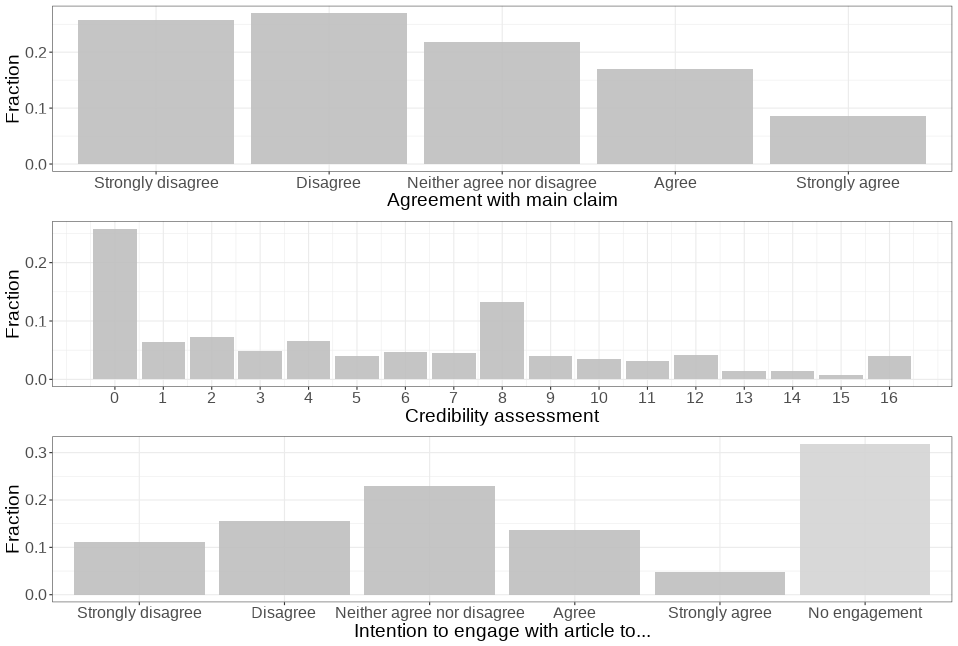


**Figure S-6. Distributions of dependent variables.** The first panel shows the distribution of ratings of agreement with the main claim of the misleading article that participants read. The middle panel depicts the distribution of the added credibility assessments on four dimensions, with response ‘4’ to individual questions meaning the misleading article is considered accurate, believable, factual and trustworthy, respectively, and ‘0’ meaning the opposite. A value of 0 here means that the misleading article was rated lowest, while 16 indicates that the misleading article was rated highest on all four dimensions. The third panel shows the distribution of reasons for intending to share the misleading article for those who indicated that they wanted to share it. It ranges from sharing with the intention to strongly disagree to sharing with the intention to strongly agree. The fraction of participants who did not intend to share the misleading article at all, and consequently did not have to indicate a reason, is also shown (no engagement).

**Table S-2. Spearman rank correlations between dependent variables**

|  | Agreement with the main claim | Credibility assessment | Intention to agree | Intention to disagree |
| --- | --- | --- | --- | --- |
| Agreement with the main claim | 1 |  |  |  |
| Credibility assessment | 0.6652 | 1 |  |  |
| Intention to agree | 0.4508 | 0.5308 | 1 |  |
| Intention to disagree | – 0.2759 | – 0.2486 | – 0.2872 | 1 |

NB: All correlations were significant at *p* < 0.001, also after Bonferroni correction for multiple tests.


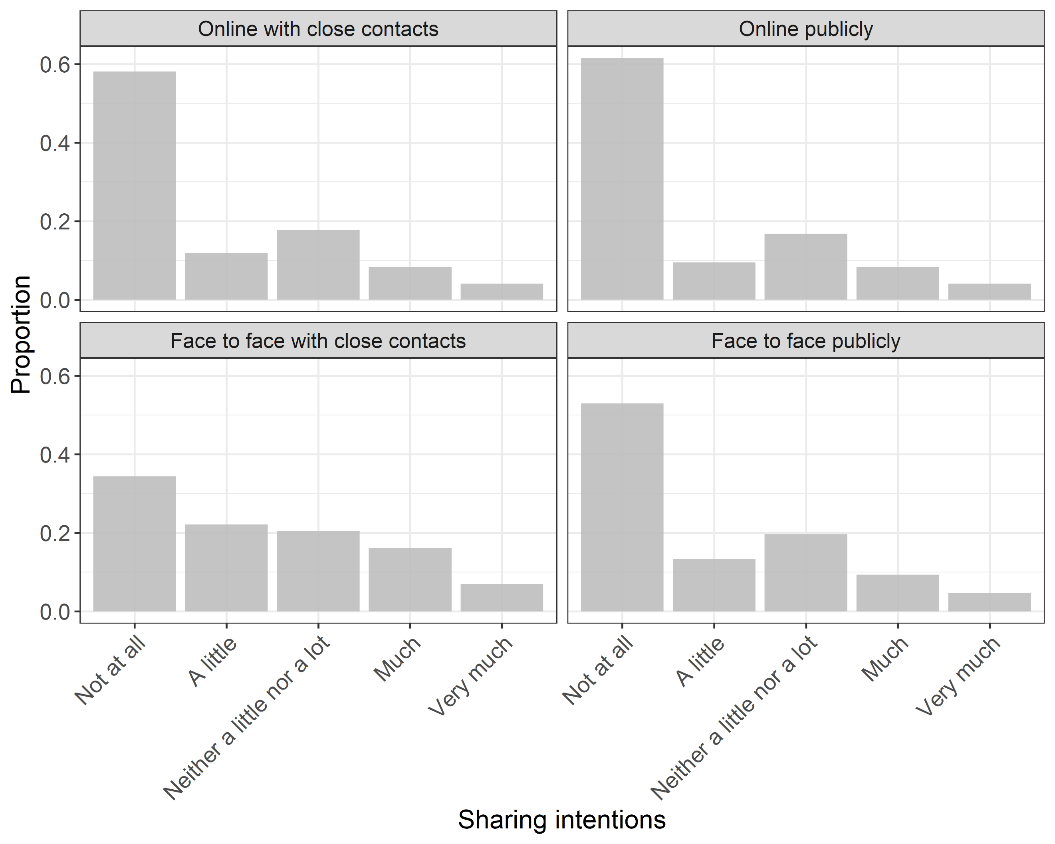


**Figure S-7. Distributions of specific sharing intentions.** Shows the proportions of people who indicated they would share the misleading article in the indicated manner (online with close contacts, online publicly, face to face with close contacts, face to face publicly).

**Table S-3. Mean values of dependent variables, by treatment.** Shows the means for the respective dependent variables by treatments and for the overall sample. Standard deviations are shown in brackets. The last row contains *p*-values of a non-parametric Kruskal–Wallis test with null hypothesis being that mean ranks are the same in all the treatments.

| Treatment | Agreement with the main claim | Credibility assessment | Intention to agree | Intention to disagree |
| --- | --- | --- | --- | --- |
| Control | 2.9 (1.31) | 6.15 (4.85) | 0.24 (0.43) | 0.24 (0.43) |
| EC debunk | 2.38 (1.21) | 4.9 (4.67) | 0.16 (0.37) | 0.29 (0.45) |
| EC prebunk | 2.54 (1.29) | 5.01 (4.73) | 0.19 (0.4) | 0.25 (0.43) |
| Neutral debunk | 2.39 (1.2) | 4.84 (4.51) | 0.14 (0.35) | 0.28 (0.45) |
| Neutral prebunk | 2.55 (1.27) | 4.95 (4.63) | 0.18 (0.38) | 0.29 (0.45) |
| Total | 2.56 (1.27) | 5.18 (4.71) | 0.18 (0.39) | 0.27 (0.44) |
| Range | (s. dis) 1–5 (s. agree) | (incred.) 0–16 (cred.) | (no) 0–1 (yes) | (no) 0–1 (yes) |
| Kwallis all treats: *p* | < 0.001 | < 0.001 | < 0.001 | 0.026 |


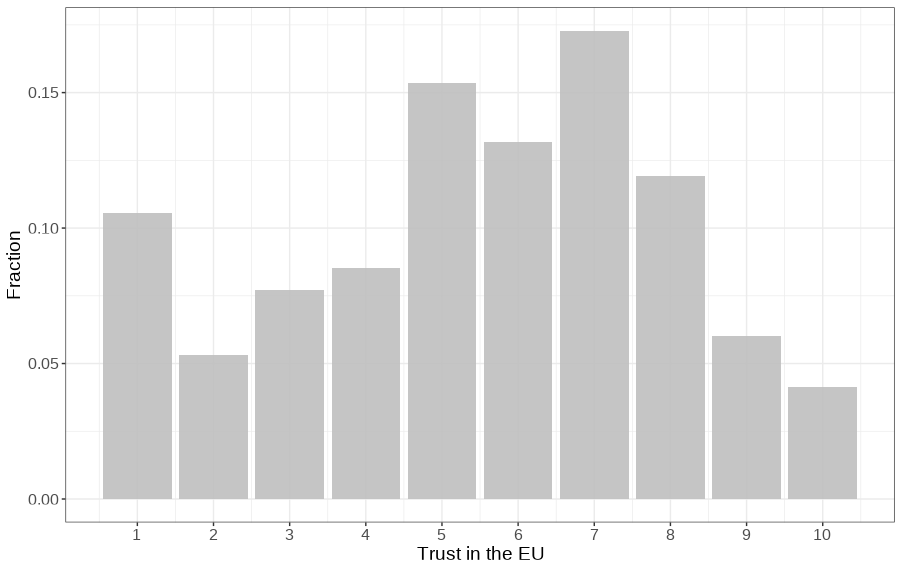


**Figure S-8. Distribution of levels of trust in the EU.** Shows the fractions of respondents with the indicated levels of trust in the EU, ranging from 1 (lowest level of trust) to 10 (highest level of trust). Data on trust in the EU is missing for 1.4 % of the sample.


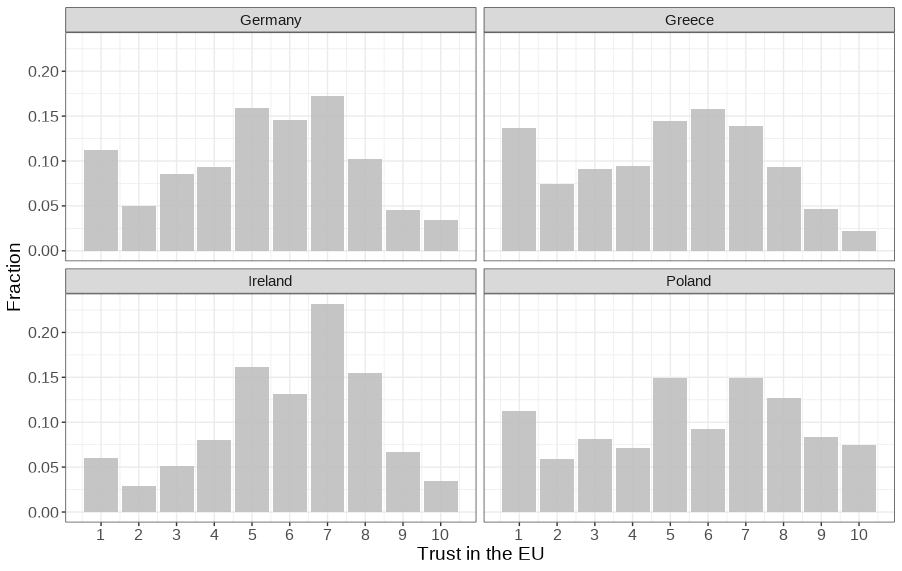


**Figure S-9. Distributions of levels of trust in the EU by country.** Shows the fractions of respondents with the indicated levels of trust in the EU, ranging from 1 (lowest level of trust) to 10 (highest level of trust), for the four different countries. Data on trust in the EU is missing for 1.4 % of the sample.


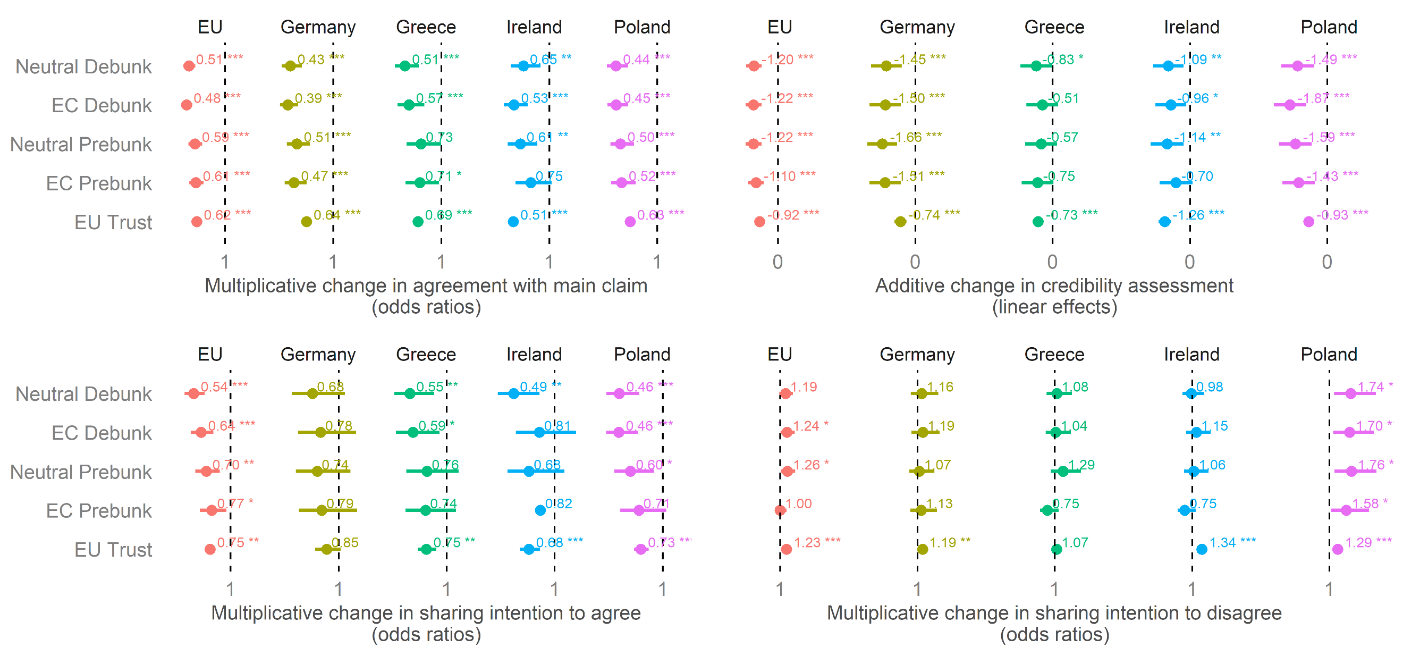


**Figure S-10.** **Country-specific effects of debunks and prebunks revealing (i.e. EC  – European Commission) or not revealing (i.e. neutral) the source of the intervention on the main outcome variables.** The *y*-axis shows the four experimental treatments (with the control as the reference condition) and standardised trust in the EU (which is not a treatment variable). The *x*-axis shows the changes in the four main outcome variables. (a) Shows the effects on agreement with the main claim shown in the misleading article from an ordered logistic regression as odds ratios; (b) shows the effects on the credibility assessment of the misleading article from a linear OLS regression as linear estimates; (c) shows the effects on intention to share the misleading article to express agreement with it (i.e. ‘sharing intention to agree’) from a binary logistic regression as odds ratios; (d) shows the effects on intention to share the misleading article to express disagreement with it (i.e. ‘sharing intention to disagree’) from a binary logistic regression as odds ratios. Effects of debunks are shown in blue and those of prebunks in red. Bars represent heteroscedasticity-robust 95 % confidence intervals. Significance levels: *** *p* < 0.001, ** *p* < 0.01, * *p* < 0.05.


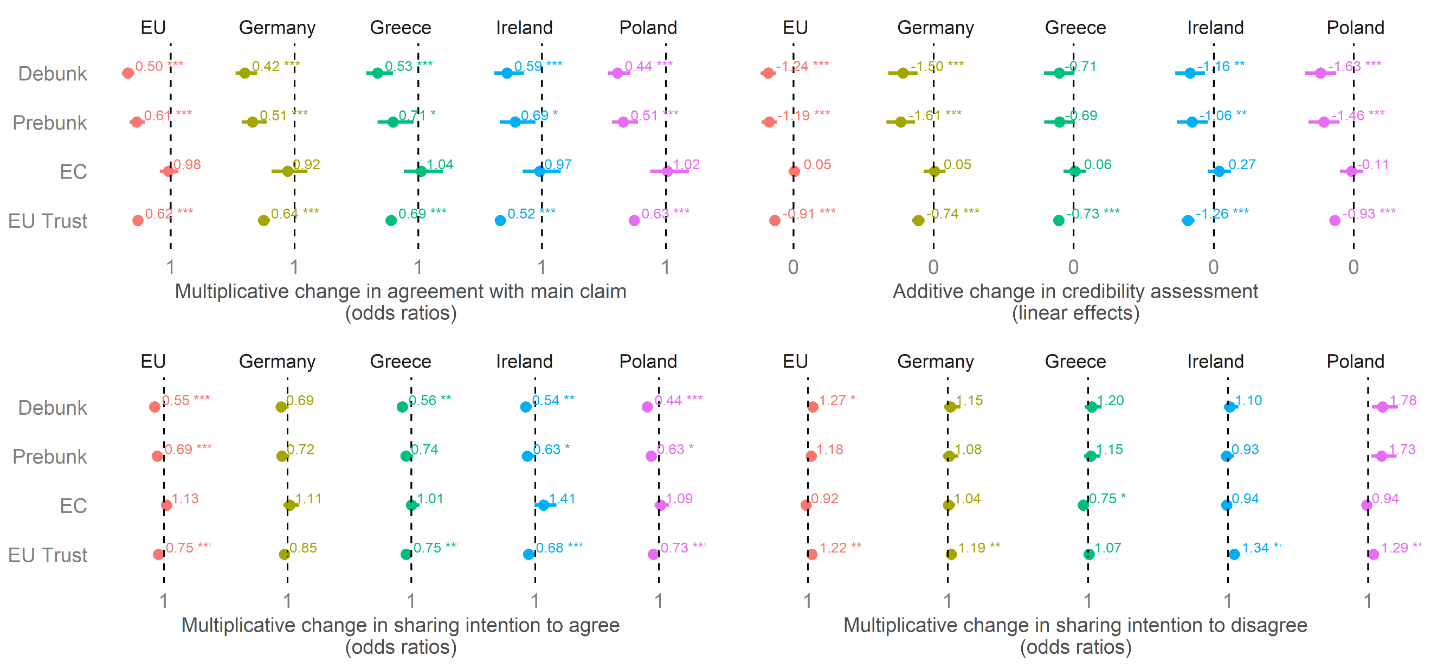


**Figure S-11. Country-specific effects of debunks, prebunks and revealing the European Commission (i.e. EC) as the intervention source on the main outcome variables.** The *y*-axis shows the interventions (with the control as the reference condition), the Commission as the source of the intervention (v neutral, i.e. no source) and standardised trust in the EU (which is not a treatment variable). The *x*-axis shows the changes in the four main outcome variables. (a) Shows the effects on agreement with the main claim shown in the misleading article from an ordered logistic regression as odds ratios; (b) shows the effects on the credibility assessment of the misleading article from a linear OLS regression as linear estimates; (c) shows the effects on intention to share the misleading article to express agreement with it (i.e. ‘sharing intention to agree’) from a binary logistic regression as odds ratios; (d) shows the effects on intention to share the misleading article to express disagreement with it (i.e. ‘sharing intention to disagree’) from a binary logistic regression as odds ratios. Bars represent heteroscedasticity-robust 95 % confidence intervals. Significance levels: *** *p* < 0.001, ** *p* < 0.01, * *p* < 0.05.

**Table S-4. Random-effect models corresponding to model specifications in Figure 2 with random intercepts for country.** Shows the estimates for the effects of the interventions versus the control, for providing the European Commission as the source with the neutral-source condition as the baseline and for standardised trust in the EU (which is not a treatment variable). Model (1) is an ordered logistic regression reporting odds ratios. Model (2) reports linear estimates from an OLS model. Models (3) and (4) report odds ratios from a binary logistic regression. For all models, a random intercept for country is included and *t*-statistics are shown in parentheses. Estimates for the EU trust level represent the change in the respective dependent variable (DV) associated with a one-standard-deviation change in the EU trust level. Significance levels: *** <. *p* < 0.001, ** < *p* < 0.01, * <. *p* < 0.05.

|  | (1)  Agreement with the main claim | (2)  Credibility assessment | (3)  Intention to agree | (4)  Intention to disagree |
| --- | --- | --- | --- | --- |
| Neutral debunk | 0.506^***^ | – 1.201^***^ | 0.541^***^ | 1.190 |
|  | (– 8.69) | (– 6.00) | (– 5.34) | (1.75) |
|  |  |  |  |  |
| EC debunk | 0.475^***^ | – 1.222^***^ | 0.634^***^ | 1.243^*^ |
|  | (– 9.50) | (– 6.09) | (– 4.10) | (2.19) |
|  |  |  |  |  |
| Neutral prebunk | 0.581^***^ | – 1.222^***^ | 0.695^***^ | 1.260^*^ |
|  | (– 6.88) | (– 6.06) | (– 3.32) | (2.31) |
|  |  |  |  |  |
| EC prebunk | 0.596^***^ | – 1.101^***^ | 0.761^*^ | 1.005 |
|  | (– 6.54) | (– 5.46) | (– 2.52) | (0.05) |
|  |  |  |  |  |
| EU trust | 0.828^***^ | – 0.366^***^ | 0.892^***^ | 1.085^***^ |
|  | (– 17.59) | (– 14.26) | (– 7.85) | (6.33) |
| lnsig2u |  |  | 0.0289^***^ | 2.11e-11 |
|  |  |  | (– 4.25) | (– 0.00) |
| Observations | 5 155 | 5 155 | 5 155 | 5 155 |

**Table S-5. Random-effect models corresponding to model specifications in Figure 3 with random intercept for country.** Shows the estimates for the effects of the prebunk and debunk versus the control, for providing the European Commission as the source with the neutral-source condition as the baseline and for standardised trust in the EU (which is not a treatment variable). Model (1) is an ordered logistic regression reporting odds ratios. Model (2) reports linear estimates from an OLS model. Models (3) and (4) report odds ratios from a binary logistic regression. For all models, a random intercept for country is included and *t*-statistics are shown in parentheses. Estimates for the EU trust level represent the change in the respective DV associated with a one-standard-deviation change in the EU trust level. Significance levels: *** <. *p* < 0.001, ** < *p* < 0.01, * <. *p* < 0.05.

|  | (1)  Agreement with the main claim | (2)  Credibility assessment | (3)  Intention to agree | (4)  Intention to disagree |
| --- | --- | --- | --- | --- |
| Debunk | 0.495^***^ | – 1.236^***^ | 0.551^***^ | 1.269^*^ |
|  | (– 9.57) | (– 6.60) | (– 5.70) | (2.56) |
|  |  |  |  |  |
| Prebunk | 0.594^***^ | – 1.186^***^ | 0.684^***^ | 1.177 |
|  | (– 7.07) | (– 6.31) | (– 3.72) | (1.74) |
|  |  |  |  |  |
| EC | 0.980 | 0.0493 | 1.131 | 0.917 |
|  | (– 0.37) | (0.34) | (1.46) | (– 1.23) |
|  |  |  |  |  |
| EU trust | 0.829^***^ | – 0.366^***^ | 0.892^***^ | 1.084^***^ |
|  | (– 17.57) | (– 14.26) | (– 7.87) | (6.27) |
| lnsig2u |  |  | 0.0289^***^ | 0.000000820 |
|  |  |  | (– 4.25) | (– 0.53) |
| Observations | 5 155 | 5 155 | 5 155 | 5 155 |

**Table S-6. Trust in the EU by country.** Shows the means of the levels of trust in the EU by country and for the overall sample. Standard deviations are shown in brackets. The last row contains the *p*-value from a non-parametric Kruskal–Wallis test with null hypothesis being that mean ranks are the same in all the treatments. There are 73 cases where respondents did not indicate trust levels. A chi-squared test indicates that missing variables are random with respect to countries (χ²(4) = 7.35, *p* = 0.119).

| Country | Trust in the EU |
| --- | --- |
| Germany | 5.29 (2.43) |
| Greece | 4.96 (2.49) |
| Ireland | 5.99 (2.21) |
| Poland | 5.62 (2.72) |
| Total | 5.46 (2.5) |
| Range | 1–10 |
| Kwallis all treats: *p* | < 0.001 |

**Table S-7.** **Average marginal effects corresponding to Figure 2.** Shows the AMEs of the interventions with the control condition as the baseline on three outcome variables. The EU trust level (which is not a treatment variable) is standardised such that the AME follows from a one-standard-deviation increase in the EU trust level. Shows standard errors, *z*-statistics and *p*-values.

| Outcome variable | Intervention | AME | SE | *z* | *p* |
| --- | --- | --- | --- | --- | --- |
| Agreement with main claim | Neutral debunk | – 0.1133 | 0.0155 | – 7.3256 | < 0.001 |
| Agreement with main claim | EC debunk | – 0.1270 | 0.0159 | – 8.0011 | < 0.001 |
| Agreement with main claim | Neutral prebunk | – 0.0860 | 0.0147 | – 5.8394 | < 0.001 |
| Agreement with main claim | EC prebunk | – 0.0820 | 0.0146 | – 5.6168 | < 0.001 |
| Agreement with main claim | EU trust | – 0.0874 | 0.0050 | – 17.3767 | < 0.001 |
| Intention to agree | Neutral debunk | – 0.0915 | 0.0169 | – 5.4057 | < 0.001 |
| Intention to agree | EC debunk | – 0.0714 | 0.0172 | – 4.1393 | < 0.001 |
| Intention to agree | Neutral prebunk | – 0.0576 | 0.0176 | – 3.2789 | 0.001 |
| Intention to agree | EC prebunk | – 0.0437 | 0.0178 | – 2.4515 | 0.0142 |
| Intention to agree | EU trust | – 0.0429 | 0.0053 | – 8.1392 | < 0.001 |
| Intention to disagree | Neutral debunk | 0.0334 | 0.0191 | 1.7487 | 0.0803 |
| Intention to disagree | EC debunk | 0.0422 | 0.0192 | 2.1955 | 0.0281 |
| Intention to disagree | Neutral prebunk | 0.0450 | 0.0194 | 2.3149 | 0.0206 |
| Intention to disagree | EC prebunk | 0.0009 | 0.0188 | 0.0474 | 0.9622 |
| Intention to disagree | EU trust | 0.0398 | 0.0062 | 6.4068 | < 0.001 |

**Table S-8. Average marginal effects corresponding to** **Figure 3.** Shows the AMEs of the interventions with either the debunk or the neutral source treatment as the baseline on three outcome variables. The EU trust level (which is not a treatment variable) is standardised such that the AME follows from a one-standard-deviation increase in the EU trust level. Shows standard errors, *z*-statistics and *p*-values.

| Outcome variable | Intervention | AME | SE | *z* | *p* |
| --- | --- | --- | --- | --- | --- |
| Agreement with main claim | Prebunk (v debunk) | 0.0364 | 0.0104 | 3.4889 | < 0.001 |
| Agreement with main claim | EC (v neutral) | – 0.0048 | 0.0109 | – 0.4380 | 0.6614 |
| Agreement with main claim | EU trust | – 0.0914 | 0.0058 | – 15.8647 | < 0.001 |
| Intention to agree | Prebunk (v debunk) | 0.0309 | 0.0117 | 2.6323 | 0.0085 |
| Intention to agree | EC (v neutral) | 0.0169 | 0.0117 | 1.4472 | 0.1479 |
| Intention to agree | EU trust | – 0.0382 | 0.0058 | – 6.6298 | < 0.001 |
| Intention to disagree | Prebunk (v debunk) | – 0.0150 | 0.0140 | – 1.0747 | 0.2825 |
| Intention to disagree | EC (v neutral) | – 0.0172 | 0.0140 | – 1.2332 | 0.2175 |
| Intention to disagree | EU trust | 0.0383 | 0.0070 | 5.4522 | < 0.001 |

**Table S-9. Average marginal effects corresponding to** **Figure 5.** Shows the AMEs of the interventions with either the debunk or the neutral source treatment as the baseline on the five outcome variables depicting intervention perceptions. The EU trust level (which is not a treatment variable) is standardised such that the AME follows from a one-standard-deviation increase in the EU trust level. Shows standard errors, *z*-statistics and *p*-values.

| Outcome variable | Intervention | AME | SE | *z* | *p* |
| --- | --- | --- | --- | --- | --- |
| Relevant | Prebunk (v debunk) | – 0.0566 | 0.0152 | – 3.7109 | < 0.001 |
| Relevant | EC (v neutral) | 0.0299 | 0.0152 | 1.9662 | 0.0493 |
| Relevant | EU trust | 0.1019 | 0.0071 | 14.3331 | < 0.001 |
| Decision enhancing | Prebunk (v debunk) | – 0.0110 | 0.0152 | – 0.7209 | 0.4710 |
| Decision enhancing | EC (v neutral) | 0.0133 | 0.0152 | 0.8761 | 0.3810 |
| Decision enhancing | EU trust | 0.1204 | 0.0070 | 17.3016 | < 0.001 |
| Authentic | Prebunk (v debunk) | – 0.0413 | 0.0151 | – 2.7253 | 0.0064 |
| Authentic | EC (v neutral) | 0.0383 | 0.0151 | 2.5299 | 0.0114 |
| Authentic | EU trust | 0.1258 | 0.0069 | 18.3589 | < 0.001 |
| Attention-grabbing | Prebunk (v debunk) | – 0.0106 | 0.0154 | – 0.6880 | 0.4914 |
| Attention-grabbing | EC (v neutral) | 0.0113 | 0.0153 | 0.7399 | 0.4594 |
| Attention-grabbing | EU trust | 0.0935 | 0.0072 | 12.8985 | < 0.001 |
| Manipulative | Prebunk (v debunk) | 0.0554 | 0.0142 | 3.9117 | < 0.001 |
| Manipulative | EC (v neutral) | – 0.0113 | 0.0141 | – 0.8022 | 0.4225 |
| Manipulative | EU trust | – 0.0357 | 0.0070 | – 5.1278 | < 0.001 |


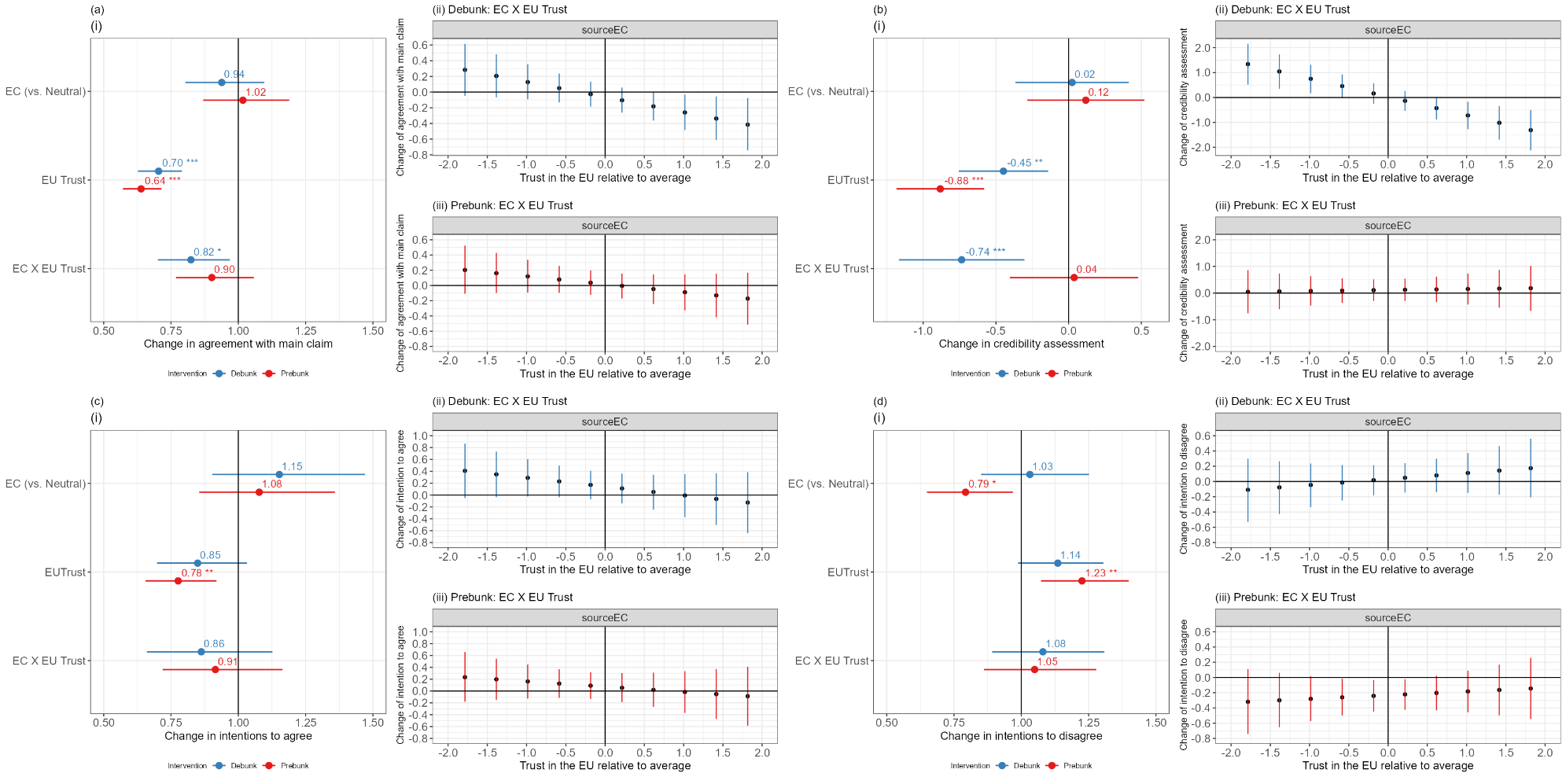


**Figure S-12.** **Interactions between trust in the EU and revealing the source of the intervention (i.e. EC  – European Commission v neutral – no source) on the main outcome variables for debunks and prebunks, and the marginal effects of the source conditional on the level of trust in the EU.** (i) The *y*-axis shows the source (EC v neutral), standardised trust in the EU (which is not a treatment variable) and their interaction. The *x*-axis shows the changes in the four main outcome variables. (ii–iii) The *y*-axis shows the changes in the four outcome variables conditional on the level of trust in the EU ((ii) for debunks (blue) and (iii) for prebunks (red)). The *x*-axis shows the level of trust in the EU relative to average trust in standard deviations; (a) shows the effects on agreement with the main claim shown in the misleading article from an ordered logistic regression in (i) as odds ratios and in (ii–iii) as marginal effects; (b) shows the effects on the credibility assessment of the misleading article from a linear OLS regression as linear estimates in (i) and in (ii–iii) as marginal effects; (c) (i) shows the effects on intention to share the misleading article to express agreement with it from a binary logistic regression in (i) as odds ratios and in (ii–iii) as marginal effects; (d) (i) shows the effects on intention to share the misleading article to express disagreement with it from a binary logistic regression in (i) as odds ratios and in (ii–iii) as marginal effects. Bars represent heteroscedasticity-robust 95 % confidence intervals. Significance levels: *** *p* < 0.001, ** *p* < 0.01, * *p* < 0.05.


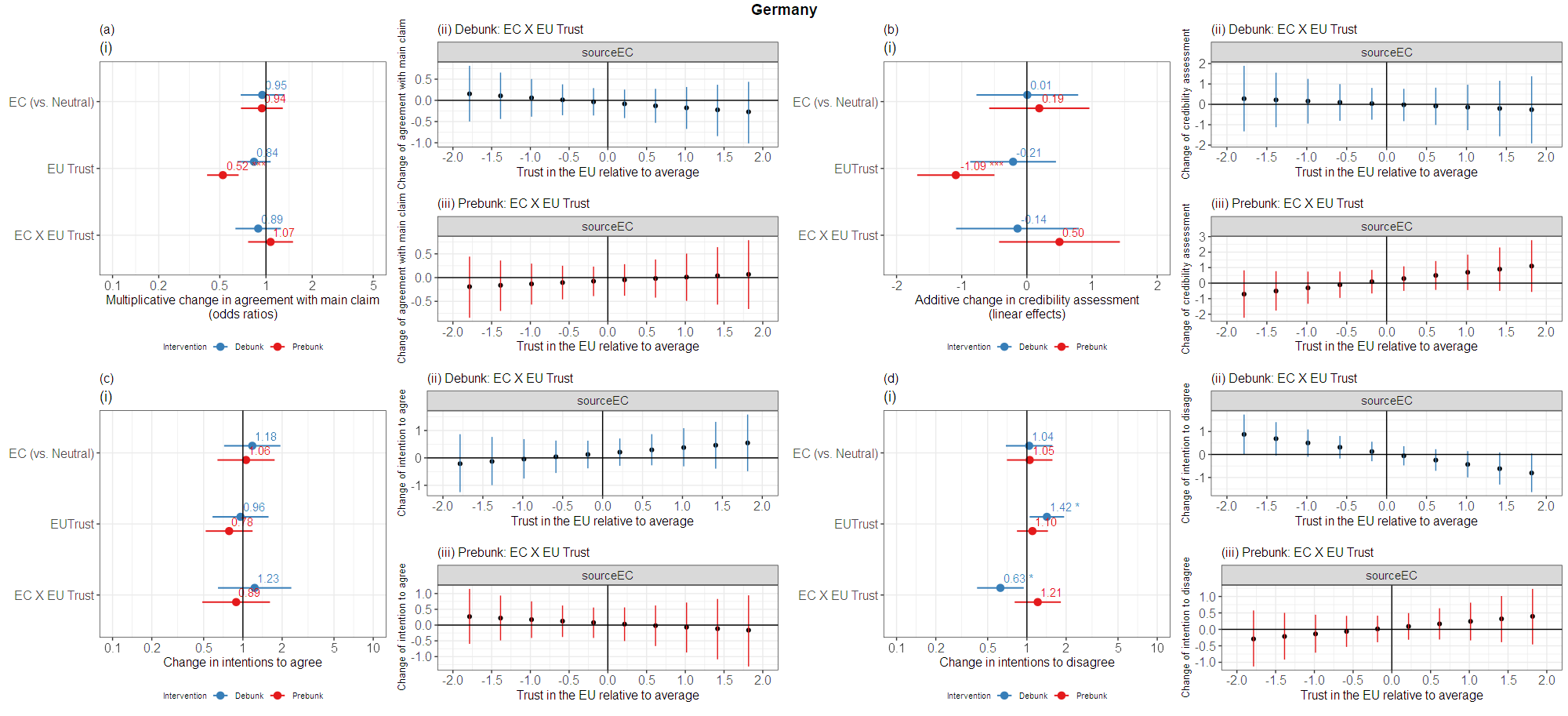


**Figure S-13.** **Interactions between the level of trust in the EU and revealing the source of the intervention (i.e. EC  – European Commission v neutral – no source) on the main outcome variables for debunks and prebunks, and the marginal effects of the source conditional on the level of trust in the EU, for the German sample.** (i) The *y*-axis shows the source (EC v neutral), standardised trust in the EU and their interaction. The *x*-axis shows the changes in the four main outcome variables. (ii–iii) The *y*-axis shows the changes in the four outcome variables conditional on the level of trust in the EU ((ii) for debunks (blue) and (iii) for prebunks (red)). The *x*-axis shows the level of trust in the EU relative to average trust in standard deviations; (a) shows the effects on agreement with the main claim shown in the misleading article from an ordered logistic regression in (i) as odds ratios and in (ii–iii) as marginal effects; (b) shows the effects on the credibility assessment of the misleading article from a linear OLS regression as linear estimates in (i) and in (ii–iii) as marginal effects; (c) (i) shows the effects on intention to share the misleading article to express agreement with it from a binary logistic regression in (i) as odds ratios and in (ii–iii) as marginal effects; (d) (i) shows the effects on intention to share the misleading article to express disagreement with it from a binary logistic regression in (i) as odds ratios and in (ii–iii) as marginal effects. Bars represent heteroscedasticity-robust 95 % confidence intervals. Significance levels: *** *p* < 0.001, ** *p* < 0.01, * *p* < 0.05.


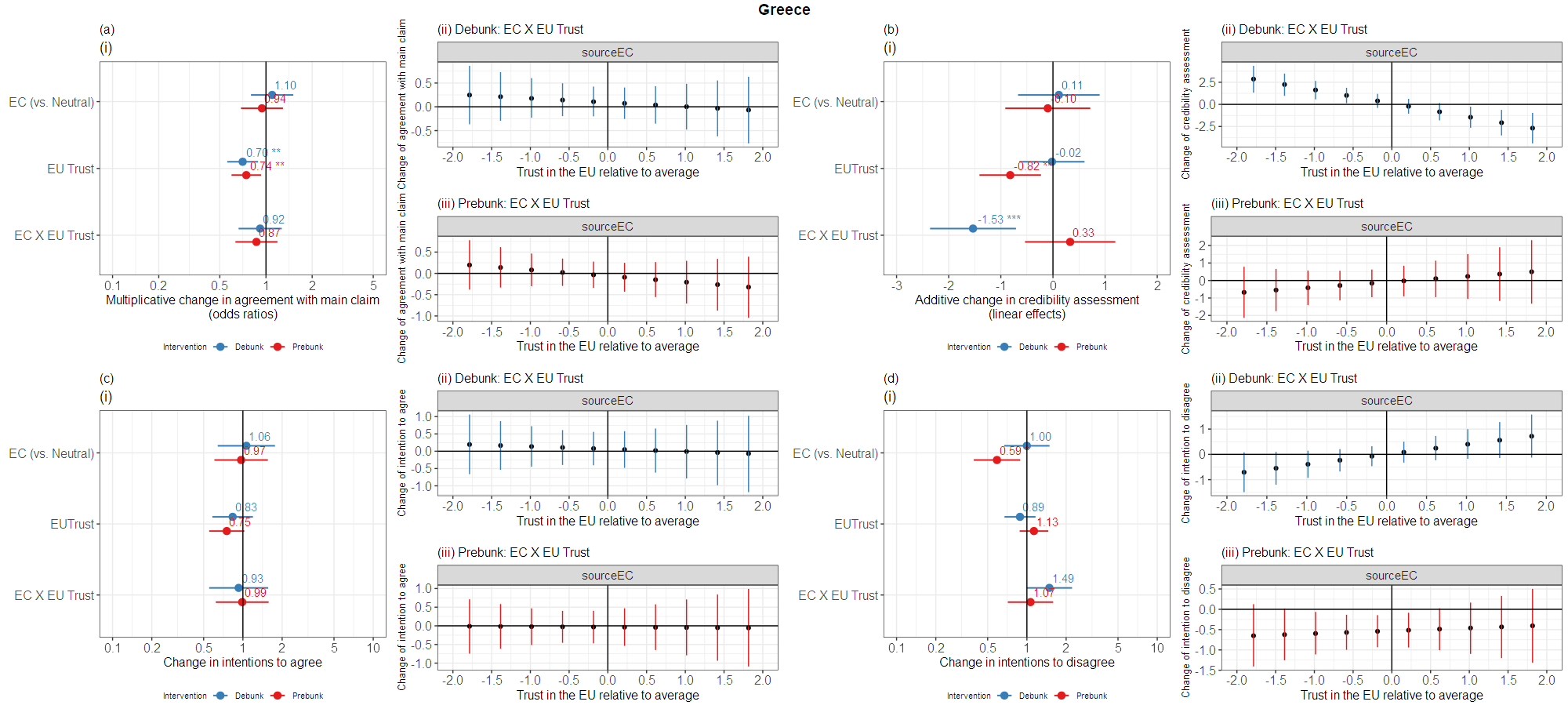


**Figure S-14. Interactions between the level of trust in the EU and revealing the source of the intervention (i.e. EC  – European Commission v neutral – no source) on the main outcome variables for debunks and prebunks, and the marginal effects of the source conditional on the level of trust in the EU, for the Greek sample.** (i) The *y*-axis shows the source (EC v neutral), standardised trust in the EU and their interaction. The *x*-axis shows the changes in the four main outcome variables. (ii–iii) The *y*-axis shows the changes in the four outcome variables conditional on the level of trust in the EU ((ii) for debunks (blue) and (iii) for prebunks (red)). The *x*-axis shows the level of trust in the EU relative to average trust in standard deviations; (a) shows the effects on agreement with the main claim shown in the misleading article from an ordered logistic regression in (i) as odds ratios and in (ii–iii) as marginal effects; (b) shows the effects on the credibility assessment of the misleading article from a linear OLS regression as linear estimates in (i) and in (ii–iii) as marginal effects; (c) (i) shows the effects on intention to share the misleading article to express agreement with it from a binary logistic regression in (i) as odds ratios and in (ii–iii) as marginal effects; (d) (i) shows the effects on intention to share the misleading article to express disagreement with it from a binary logistic regression in (i) as odds ratios and in (ii–iii) as marginal effects. Bars represent heteroscedasticity-robust 95 % confidence intervals. Significance levels: *** *p* < 0.001, ** *p* < 0.01, * *p* < 0.05.


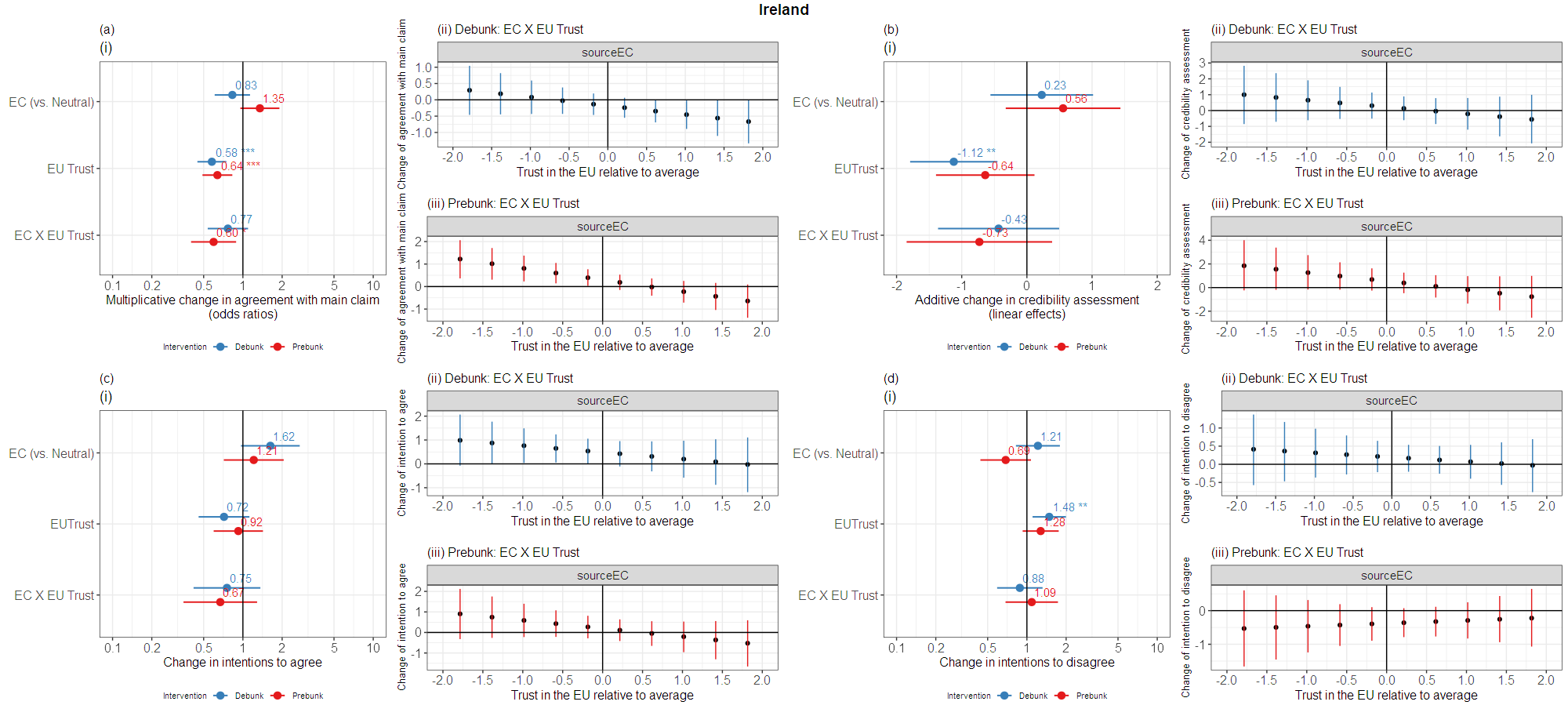


**Figure S-15.** **Interactions between the level of trust in the EU and revealing the source of the intervention (i.e. EC  – European Commission v neutral – no source) on the main outcome variables for debunks and prebunks, and the marginal effects of the source conditional on the level of trust in the EU, for the Irish sample.** (i) The *y*-axis shows the source (EC v neutral), standardised trust in the EU and their interaction. The *x*-axis shows the changes in the four main outcome variables. (ii–iii) The *y*-axis shows the changes in the four outcome variables conditional on the level of trust in the EU ((ii) for debunks (blue) and (iii) for prebunks (red)). The *x*-axis shows the level of trust in the EU relative to average trust in standard deviations; (a) shows the effects on agreement with the main claim shown in the misleading article from an ordered logistic regression in (i) as odds ratios and in (ii–iii) as marginal effects; (b) shows the effects on the credibility assessment of the misleading article from a linear OLS regression as linear estimates in (i) and in (ii–iii) as marginal effects; (c) (i) shows the effects on intention to share the misleading article to express agreement with it from a binary logistic regression in (i) as odds ratios and in (ii–iii) as marginal effects; (d) (i) shows the effects on intention to share the misleading article to express disagreement with it from a binary logistic regression in (i) as odds ratios and in (ii–iii) as marginal effects. Bars represent heteroscedasticity-robust 95 % confidence intervals. Significance levels: *** *p* < 0.001, ** *p* < 0.01, * *p* < 0.05.


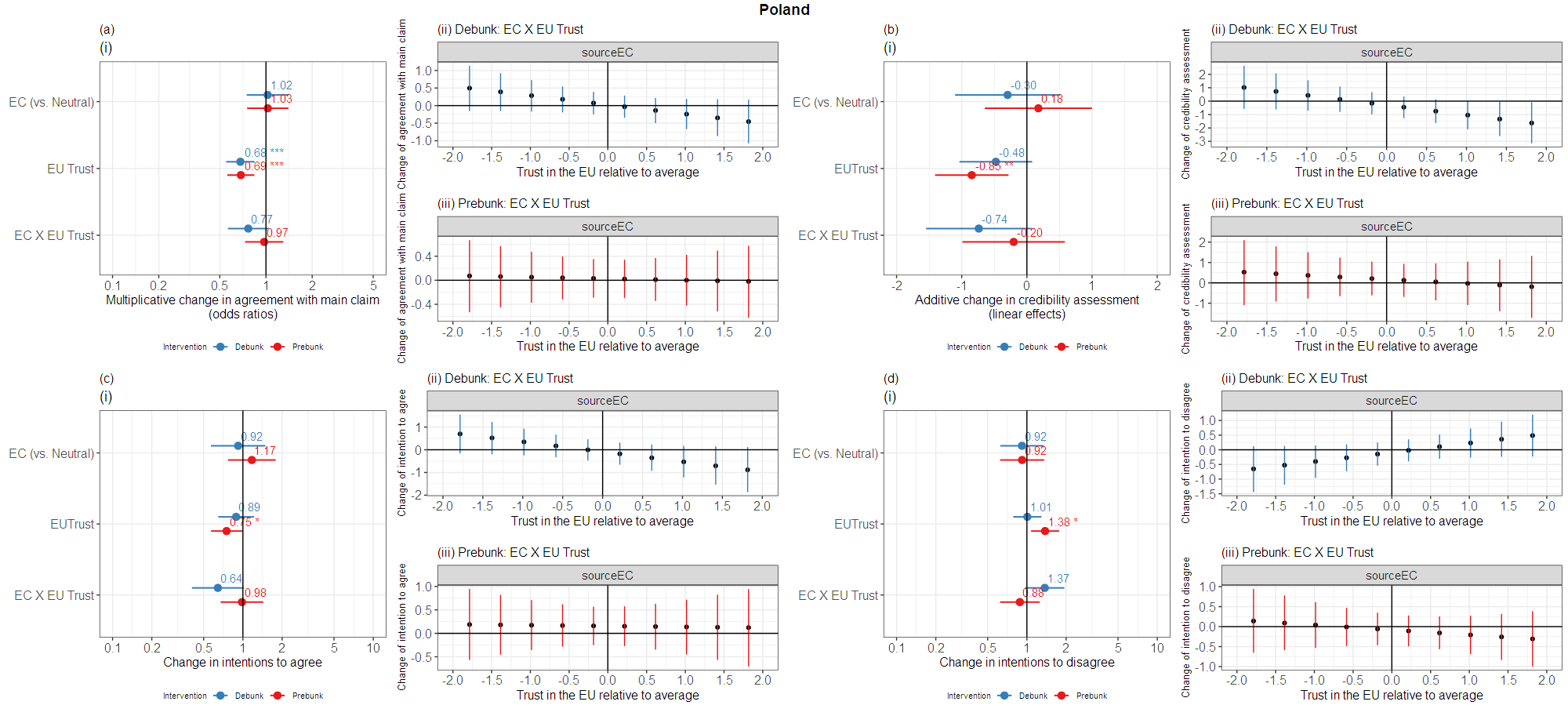


**Figure S-16.** **Interactions between the level of trust in the EU and revealing the source of the intervention (i.e. EC  – European Commission v neutral – no source) on the main outcome variables for debunks and prebunks, and the marginal effects of the source conditional on the level of trust in the EU, for the Polish sample.** (i) The *y*-axis shows the source (EC v neutral), standardised trust in the EU and their interaction. The *x*-axis shows the changes in the four main outcome variables. (ii–iii) The *y*-axis shows the changes in the four outcome variables conditional on the level of trust in the EU ((ii) for debunks (blue) and (iii) for prebunks (red)). The *x*-axis shows the level of trust in the EU relative to average trust in standard deviations; (a) shows the effects on agreement with the main claim shown in the misleading article from an ordered logistic regression in (i) as odds ratios and in (ii–iii) as marginal effects; (b) shows the effects on the credibility assessment of the misleading article from a linear OLS regression as linear estimates in (i) and in (ii–iii) as marginal effects; (c) (i) shows the effects on intention to share the misleading article to express agreement with it from a binary logistic regression in (i) as odds ratios and in (ii–iii) as marginal effects; (d) (i) shows the effects on intention to share the misleading article to express disagreement with it from a binary logistic regression in (i) as odds ratios and in (ii–iii) as marginal effects. Bars represent heteroscedasticity-robust 95 % confidence intervals. Significance levels: *** *p* < 0.001, ** *p* < 0.01, * *p* < 0.05.


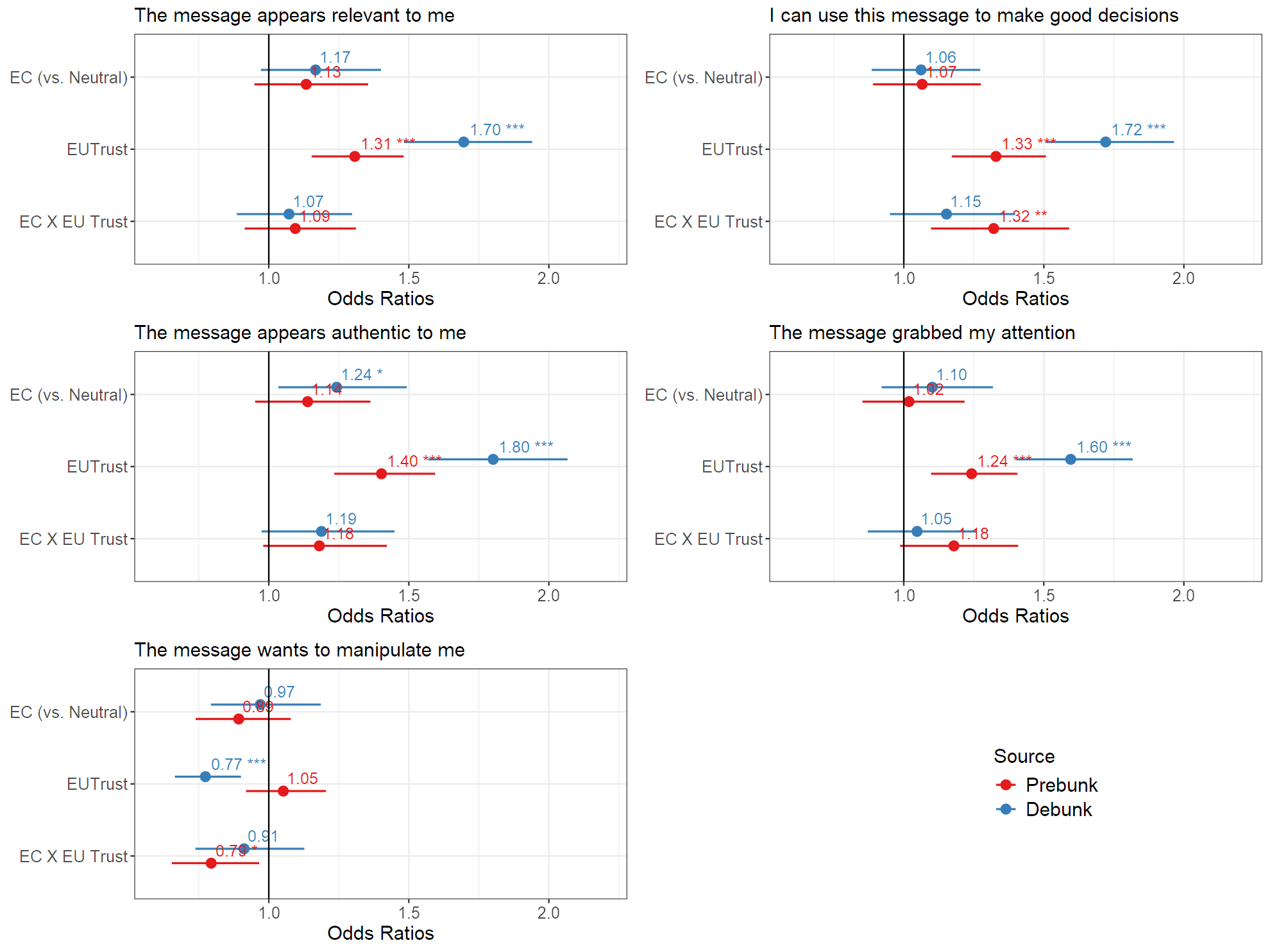


**Figure S-17.** **Interactions between the level of trust in the EU and the source on the perceptions of debunks and prebunks.** Shows the estimates for the effects of the European Commission source for people with an average level of trust in the EU, the correlation between standardised trust in the EU and the outcome for people in the neutral source treatment, and the interaction of the source information and the level of trust in the EU, both for debunks (blue) and prebunks (red), on the perceptions of the interventions with regard to being relevant, decision-enhancing, authentic, attention-grabbing and manipulative. Outcome variables are 1 if participants (strongly) agreed and 0 otherwise. Participants from the control condition are not included, as they saw no intervention that they could have rated. Estimates for the EU trust level represent the change in the respective DV associated with a one-standard-deviation change in the EU trust level. The models are binary logistic regressions reporting the odds ratios and heteroscedasticity-robust 95 % confidence intervals. Significance levels: *** *p* < 0.001, ** *p* < 0.01, * *p* < 0.05.

## Comparing misinformation on COVID-19 and climate change

The participants were randomly divided into two groups, with half reading articles containing misleading claims about COVID-19 and the other half reading articles containing misleading claims about climate change. To examine whether the effectiveness of our interventions varies depending on the topic, separate regressions were conducted for COVID-19 and climate change. Since no specific hypotheses were preregistered regarding the moderating effect of the topic, these analyses are exploratory. The relevant tables and plots are provided: Table S-14 presents the main effects with interaction between the intervention and the topic, while Table S-15 and Table S-16 show the focal effects interacted with the topic. Figure S-18 displays the main effects by topic and Figure S-19–Figure S-22 depict the interaction effects by topic. In addition, Figure S-23–Figure S-26 illustrate the effects of the source conditional on the levels of trust in the EU for each topic.

The main effects of the interventions on agreement with the main claim were nearly identical for COVID-19 and climate change. However, the effects for the remaining three dependent variables (credibility assessment, intention to agree, intention to disagree) were generally weaker for climate change compared with COVID-19. Both debunks and prebunks reduced the credibility assessment for both topics, but the effects were less pronounced for climate change (though not statistically significantly so). The same pattern occurs for behavioural intentions. Interestingly, all interventions reduced the likelihood of intending to agree with the main claim for COVID-19, while only the neutral debunk was effective for climate change. Regarding intention to disagree, only the debunks reduced the likelihood of this for COVID-19, but none of the interventions had a similar effect for climate change. Although prebunks and debunks appeared to be more effective in addressing COVID-19 misinformation overall, none of the interaction effects reached significance (see Table S-14 and Figure S-18).

To examine whether the main interactions of interest between revealing the source and the level of trust in the EU are sensitive to the topic of misinformation, we visually inspected forest plots showing the effects of providing source information, EU trust levels and their interaction separately for the interventions and the topic (Figure S-19- Figure S-22). We also inspected conditional effect plots (Figure S-23–Figure S-26) and estimated models with a three-way-interaction between the source, the EU trust level and the topic (see Table S-15 and Table S-16). Despite some visual differences, none of the interactions was statistically significant. Notably, the significant interaction of revealing the source for debunks mentioned in the findings section is insignificant for both climate change and COVID-19 (albeit slightly stronger for COVID-19). By pooling the data for both topics, the narrower confidence intervals allow for more precise estimates, therefore leading to a significant interaction. Judging by the conditional effect plots, the more pronounced effect for European Commission debunks for people with high levels of trust in the EU appears to occur only for the COVID-19 topic. The opposite is the case for the interaction between revealing the source of the debunk and EU trust levels on credibility assessment. The ‘backfire effect’ of revealing the Commission as the source of the intervention for people with low levels of trust in the EU and the higher effectiveness for high-trust individuals appear to occur mainly for climate change.

Regarding intention to share the misleading article or talk about it, there are no major differences with respect to the topic. Notably, except for the effects of debunks on intention to disagree (Figure S-22), the interactions go in opposite directions both for COVID-19 and climate change (Figure S-19–Figure S-21). The interaction is qualitatively counterintuitive in some cases, as can be seen from the slightly positive slopes of the conditional effect plots (see Figure S-23 and Figure S-24 for climate change and Figure S-25 and Figure S-26 for COVID-19).

Interestingly, there are instances where the interaction between the topic and the EU trust level is significant (see Table S-15 and Table S-16). However, there is no consistent pattern regarding the significance and direction of these interactions. Two interactions are positive, indicating that the association between the level of trust in the EU and the outcome variable is stronger for climate change compared with COVID-19. These cases include prebunk effects on agreement with the main claim (OR = 1.36, CI_95_ = (1.08–1.71), *p* = 0.009) and debunk effects on intention to agree (OR = 1.58, CI_95_ = (1.11–2.25), *p* = 0.023). In two cases, the interaction is negative, suggesting that the correlation between the level of trust in the EU and the outcome variable is more pronounced for misleading COVID-19 than for misleading climate change articles. These cases include prebunk effects on agreement with the main claim (OR = 0.74, CI_95_ = (0.12–1.35), *p* = 0.019) and prebunk effects on intention to disagree (OR = 0.7, CI_95_ = (0.53–0.93), *p* = 0.01).

**Table S-10. Effects of debunks and prebunks revealing (i.e. European Commission) or not revealing (i.e. neutral) the source on the main outcome variables.** Shows the estimates for the intervention effects with the control condition as the baseline and for standardised trust in the EU. Model (1) is an ordered logistic regression reporting the odds ratios. Model (2) reports linear estimates from an OLS model. Models (3) and (4) report odds ratios from a binary logistic regression. For all models, heteroscedasticity-robust 95 % confidence intervals and *p*-values are provided. Estimates for the EU trust level represent the change in the respective DV associated with a one-standard-deviation change in the EU trust level.

|  | (1) | | | (2) | | | (3) | | | (4) | | |
| --- | --- | --- | --- | --- | --- | --- | --- | --- | --- | --- | --- | --- |
|  | Agreement with the main claim | | | Credibility assessment | | | Intention to agree | | | Intention to disagree | | |
| *Predictors* | *Odds ratios* | *CI* | p | *Estimates* | *CI* | p | *Odds ratios* | *CI* | p | *Odds ratios* | *CI* | p |
| Intercept |  |  |  | 6.10 | 5.82–6.39 | < 0.001 | 0.30 | 0.26–0.35 | < 0.001 | 0.32 | 0.28–0.37 | < 0.001 |
| Neutral debunk | 0.51 | 0.44–0.60 | < 0.001 | – 1.20 | – 1.60 - – 0.81 | < 0.001 | 0.54 | 0.43–0.68 | < 0.001 | 1.19 | 0.98–1.45 | 0.081 |
| EC debunk | 0.48 | 0.41–0.56 | < 0.001 | – 1.22 | – 1.62 - – 0.83 | < 0.001 | 0.64 | 0.51–0.79 | < 0.001 | 1.24 | 1.02–1.51 | 0.028 |
| Neutral prebunk | 0.59 | 0.51–0.69 | < 0.001 | – 1.22 | – 1.62 - – 0.82 | < 0.001 | 0.70 | 0.57–0.87 | 0.001 | 1.26 | 1.04–1.53 | 0.021 |
| EC prebunk | 0.61 | 0.52–0.71 | < 0.001 | – 1.10 | – 1.50 - – 0.70 | < 0.001 | 0.77 | 0.62–0.95 | 0.014 | 1.00 | 0.82–1.23 | 0.962 |
| EU trust | 0.62 | 0.58–0.66 | < 0.001 | – 0.92 | – 1.05 - – 0.78 | < 0.001 | 0.75 | 0.70–0.80 | < 0.001 | 1.23 | 1.15–1.31 | < 0.001 |
| Observations | 5 155 | | | 5 155 | | | 5 155 | | | 5 155 | | |
| R^2^ Nagelkerke | 0.129 | | | 0.049/0.048 | | | 0.022 | | | 0.01 | | |

NB: Intercepts for the ordered logit model are strongly disagree–disagree: 0.2, (0.18–0.23), *p* < 0.001; disagree–neither agree nor disagree: 0.7, (0.63–0.79), *p* < 0.001; neither agree nor disagree–agree: 1.93, (1.72–2.17), *p* < 0.001; agree–strongly agree: 7.52, (6.57–8.61), *p* < 0.001.
**Table S-11. Effects of debunks, prebunks and revealing the source on the main outcome variables.** Shows the estimates for the effects of the debunk and prebunk versus the control, for providing the European Commission as the source with the neutral-source condition as the baseline and for standardised trust in the EU. Model (1) is an ordered logistic regression reporting the odds ratios. Model (2) reports linear estimates from an OLS model. Models (3) and (4) report odds ratios from a binary logistic regression. For all models, *heteroscedasticity-robust* 95 % confidence intervals and *p*-values are provided. Estimates for the EU trust level represent the change in the respective DV associated with a one-standard-deviation change in the EU trust level.

|  | (1) | | | | | (2) | | | | (3) | | | | (4) | | |
| --- | --- | --- | --- | --- | --- | --- | --- | --- | --- | --- | --- | --- | --- | --- | --- | --- |
|  | Agreement with the main claim | | | | | Credibility assessment | | | | Intention to agree | | | | Intention to disagree | | |
| *Predictors* | | *Odds ratios* | *CI* | p | *Estimates* | | *CI* | p | *Odds ratios* | | *CI* | p | *Odds ratios* | | *CI* | p |
| Intercept | |  |  |  | 6.10 | | 5.82–6.39 | < 0.001 | 0.30 | | 0.26–0.35 | < 0.001 | 0.32 | | 0.28–0.37 | < 0.001 |
| Debunk | | 0.50 | 0.44–0.58 | < 0.001 | – 1.24 | | – 1.61 - – 0.87 | < 0.001 | 0.55 | | 0.45–0.68 | < 0.001 | 1.27 | | 1.06–1.52 | 0.010 |
| Prebunk | | 0.61 | 0.53–0.70 | < 0.001 | – 1.19 | | – 1.56 - – 0.81 | < 0.001 | 0.69 | | 0.57–0.84 | < 0.001 | 1.18 | | 0.98–1.42 | 0.083 |
| EC (v neutral) | | 0.98 | 0.87–1.09 | 0.659 | 0.05 | | – 0.23–0.33 | 0.730 | 1.13 | | 0.96–1.33 | 0.148 | 0.92 | | 0.80–1.05 | 0.219 |
| EU trust | | 0.62 | 0.59–0.65 | < 0.001 | – 0.91 | | – 1.05 - – 0.77 | < 0.001 | 0.75 | | 0.70–0.80 | < 0.001 | 1.22 | | 1.15–1.30 | < 0.001 |
| Observations | | 5 155 | | | 5 155 | | | | 5 155 | | | | 5 155 | | | |
| R^2^ Nagelkerke | | 0.128 | | | 0.049/0.049 | | | | 0.022 | | | | 0.009 | | | |

NB: Intercepts for the ordered logit model for agreement as DV are strongly disagree–disagree: 0.20, (0.18–0.23), *p* < 0.001; disagree–neither agree nor disagree: 0.70, (0.63–0.79), *p* < 0.001; neither agree nor disagree–agree: 1.93, (1.73–2.16), *p* < 0.001; agree–strongly agree: 7.52, (6.59–8.59), *p* < 0.001.

**Table S-12. Effects of revealing the source of the interventions and interaction with the EU trust level on beliefs and credibility ratings.** Shows the estimates for the effects of providing the Commission as the source with the neutral-source condition as the baseline and for standardised trust in the EU. Models (1) and (2) are ordered logistic regression reporting the odds ratios. Models (3) and (4) report linear estimates from OLS models. For all models, heteroscedasticity-robust 95 % confidence intervals and *p*-values are provided. The EU trust level is de-meaned and standardised such that 0 corresponds to the average level of trust in the EU and estimates for the EU trust level represent the change in the respective DV associated with a one-standard-deviation change in the EU trust level.

|  | Agreement with the main claim | | | | | | Credibility assessment | | | | | |
| --- | --- | --- | --- | --- | --- | --- | --- | --- | --- | --- | --- | --- |
|  | (1) | | | (2) | | | (3) | | | (4) | | |
| Intervention | Debunk | | | Prebunk | | | Debunk | | | Prebunk | | |
| *Predictors* | *Odds ratios* | *CI* | p | *Odds ratios* | *CI* | p | *Estimates* | *CI* | p | *Ratios* | *CI* | p |
| Intercept |  |  |  |  |  |  | 4.86 | 4.58–5.13 | < 0.001 | 4.88 | 4.60–5.17 | < 0.001 |
| EC v neutral intervention | 0.94 | 0.80–1.10 | 0.422 | 1.02 | 0.87–1.19 | 0.833 | 0.02 | – 0.37–0.41 | 0.904 | 0.12 | – 0.28–0.52 | 0.565 |
| EU trust | 0.70 | 0.62–0.80 | < 0.001 | 0.64 | 0.57–0.72 | < 0.001 | – 0.45 | – 0.75 - – 0.14 | 0.004 | – 0.88 | – 1.18 - – 0.58 | < 0.001 |
| EC intervention × EU trust | 0.82 | 0.69–0.98 | 0.033 | 0.90 | 0.76–1.07 | 0.238 | – 0.74 | – 1.17 – 0.30 | 0.001 | 0.04 | – 0.40–0.48 | 0.866 |
| Observations | 2 066 | | | 2 012 | | | 2 066 | | | 2 012 | | |
| R^2^ Nagelkerke | 0.115 | | | 0.105 | | | 0.0.38/0.036 | | | 0.035/0.033 | | |

NB: Intercepts for the ordered logit model of debunks are strongly disagree–disagree: 0.37, (0.32–0.41), *p* < 0.001; disagree–neither agree nor disagree: 1.42, (1.27–1.60), *p* < 0.001; neither agree nor disagree–agree: 4.13, (3.60–4.72), *p* < 0.001; agree–strongly agree: 15.56, (12.80–18.92), *p* < 0.001. Intercepts for ordered logit models of prebunks are strongly disagree–disagree: 0.36, (0.32–0.41), *p* < 0.001; disagree–neither agree nor disagree: 1.15, (1.02–1.29), *p* = 0.023; neither agree nor disagree–agree: 3.11, (2.72–3.55), *p* < 0.001; agree–strongly agree: 12.54, (10.44–15.06), *p* < 0.001.

**Table S-13. Effects of revealing the source of the interventions and interaction with the EU trust level on intentions to endorse and criticise.** Shows the estimates for the effects of providing the Commission as the source with the neutral-source condition as the baseline. All models report odds ratios from binary logistic regressions. For all models, heteroscedasticity-robust 95 % confidence intervals and *p*-values are provided. The EU trust level is de-meaned and standardised such that 0 corresponds to the average level of trust in the EU and estimates for the EU trust level represent the change in the respective DV associated with a one-standard-deviation change in the EU trust level.

|  | Intention to agree | | | | | | Intention to disagree | | | | | |
| --- | --- | --- | --- | --- | --- | --- | --- | --- | --- | --- | --- | --- |
|  | (1) | | | (2) | | | (3) | | | (4) | | |
| Intervention | Debunk | | | Prebunk | | | Debunk | | | Prebunk | | |
| *Predictors* | *Odds ratios* | *CI* | p | *Odds ratios* | *CI* | p | *Odds ratios* | *CI* | p | *Ratios* | *CI* | p |
| Intercept | 0.17 | 0.14–0.20 | < 0.001 | 0.22 | 0.18–0.25 | < 0.001 | 0.39 | 0.34–0.45 | < 0.001 | 0.41 | 0.35–0.47 | < 0.001 |
| EC v neutral intervention | 1.15 | 0.90–1.47 | 0.254 | 1.08 | 0.86–1.36 | 0.527 | 1.03 | 0.85–1.25 | 0.750 | 0.79 | 0.65–0.97 | 0.023 |
| EU trust | 0.85 | 0.71–1.01 | 0.100 | 0.78 | 0.66–0.91 | 0.003 | 1.14 | 0.99–1.31 | 0.072 | 1.23 | 1.07–1.41 | 0.003 |
| EC intervention × EU trust | 0.86 | 0.68–1.09 | 0.276 | 0.91 | 0.73–1.14 | 0.468 | 1.08 | 0.89–1.31 | 0.429 | 1.05 | 0.86–1.28 | 0.631 |
| Observations | 2 066 | | | 2 012 | | | 2 066 | | | 2 012 | | |
| R^2^ Nagelkerke | 0.011 | | | 0.016 | | | 0.006 | | | 0.012 | | |


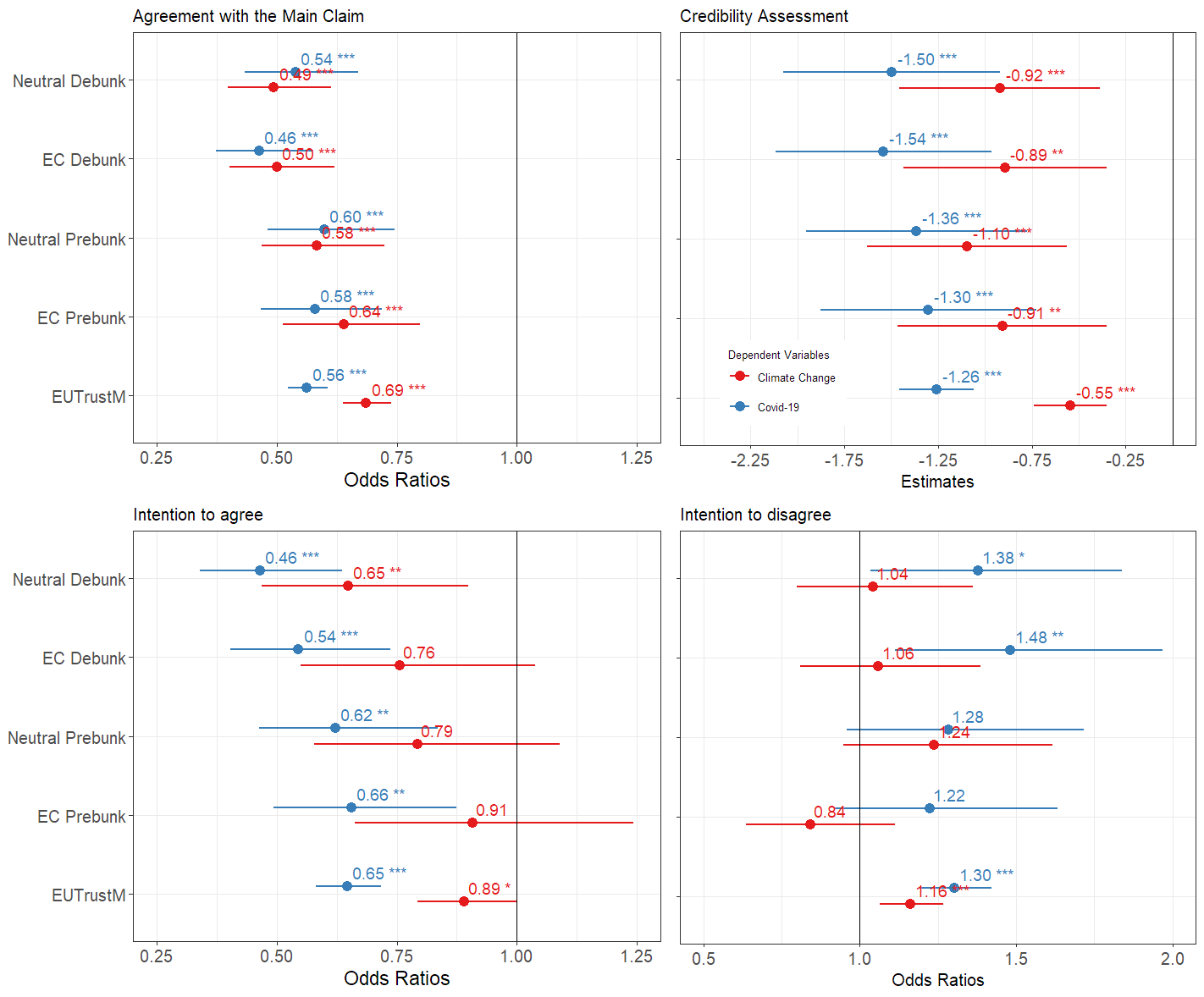


**Figure S-18. Effects of the interventions on the main outcome variables by misleading** **article topic (climate change in red and COVID-19 in blue).** The *y*-axis shows the interventions (with the control as the reference condition) and standardised trust in the EU (which is not a treatment variable). The *x*-axis shows the changes in the four main outcome variables. (a) Shows the effects on agreement with the main claim shown in the misleading article from an ordered logistic regression as odds ratios; (b) shows the effects on the credibility assessment of the misleading article from a linear OLS regression as linear estimates; (c) shows the effects on intention to agree with the misleading article from a binary logistic regression as odds ratios; (d) shows the effects on intention to disagree with the misleading article from a binary logistic regression as odds ratios. Bars represent heteroscedasticity-robust 95 % confidence intervals. Significance levels: *** *p* < 0.001, ** *p* < 0.01, * *p* < 0.05.

**Table S-14.** **Results from models for the four main outcome variables showing the interactions of the misleading** **article topic and the intervention.** Shows the estimates for the intervention effects with the control condition as the baseline, interacted with the topic of the misinformation (climate change v COVID-19, the latter being the baseline). Model (1) is an ordered logistic regression reporting the odds ratios. Model (2) reports linear estimates from an OLS model. Models (3) and (4) report odds ratios from a binary logistic regression. For all models, heteroscedasticity-robust 95 % confidence intervals and *p*-values are provided. Estimates for the EU trust level represent the change in the respective DV associated with a one-standard-deviation change in the EU trust level.

|  | Agreement with main claim | | | Credibility assessment | | | Intention to agree | | | | | Intention to disagree | | | | |
| --- | --- | --- | --- | --- | --- | --- | --- | --- | --- | --- | --- | --- | --- | --- | --- | --- |
| *Predictors* | *Odds ratios* | *CI* | p | *Estimates* | *CI* | p | *Odds ratios* | | *CI* | p | | *Odds ratios* | | | *CI* | p |
| Neutral debunk | 0.53 | 0.42–0.66 | < 0.001 | – 1.55 | – 2.12 - – 0.97 | < 0.001 | | 0.46 | 0.34–0.63 | | < 0.001 | | 1.39 | 1.04–1.85 | | 0.025 |
| EC debunk | 0.46 | 0.37–0.57 | < 0.001 | – 1.56 | – 2.14 - – 0.99 | < 0.001 | | 0.55 | 0.40–0.73 | | < 0.001 | | 1.48 | 1.12–1.97 | | 0.006 |
| Neutral prebunk | 0.60 | 0.48–0.75 | < 0.001 | – 1.36 | – 1.95 - – 0.76 | < 0.001 | | 0.63 | 0.47–0.84 | | 0.002 | | 1.28 | 0.96–1.71 | | 0.095 |
| EC prebunk | 0.57 | 0.46–0.72 | < 0.001 | – 1.32 | – 1.90 - – 0.74 | < 0.001 | | 0.66 | 0.49–0.87 | | 0.003 | | 1.23 | 0.92–1.64 | | 0.163 |
| Climate change (v COVID-19) | 0.99 | 0.80–1.23 | 0.936 | – 0.40 | – 0.96–0.16 | 0.166 | | 0.63 | 0.47–0.84 | | 0.002 | | 1.48 | 1.12–1.96 | | 0.006 |
| EU trust | 0.62 | 0.59–0.66 | < 0.001 | – 0.91 | – 1.05 - – 0.77 | < 0.001 | | 0.75 | 0.70–0.80 | | < 0.001 | | 1.23 | 1.15–1.31 | | < 0.001 |
| Neutral debunk × climate change | 0.95 | 0.70–1.29 | 0.753 | 0.69 | – 0.10–1.47 | 0.088 | | 1.44 | 0.92–2.26 | | 0.114 | | 0.74 | 0.50–1.10 | | 0.140 |
| EC debunk × climate change | 1.10 | 0.81–1.48 | 0.547 | 0.69 | – 0.10–1.48 | 0.089 | | 1.39 | 0.90–2.15 | | 0.139 | | 0.71 | 0.48–1.05 | | 0.088 |
| Neutral prebunk × climate change | 0.98 | 0.71–1.34 | 0.885 | 0.27 | – 0.53–1.07 | 0.504 | | 1.26 | 0.82–1.94 | | 0.296 | | 0.97 | 0.65–1.43 | | 0.862 |
| EC prebunk × climate change | 1.12 | 0.82–1.54 | 0.472 | 0.44 | – 0.36–1.25 | 0.279 | | 1.40 | 0.92–2.14 | | 0.120 | | 0.68 | 0.46–1.02 | | 0.061 |
| (Intercept) |  |  |  | 6.30 | 5.89–6.72 | < 0.001 | | 0.38 | 0.31–0.46 | | < 0.001 | | 0.26 | 0.21–0.32 | | < 0.001 |
| Observations | 5 155 | | | 5 155 | | | 5 155 | | | | | 5 155 | | | | |
| R^2^ Nagelkerke | 0.129 | | | 0.050/0.048 | | | 0.025 | | | | | 0.012 | | | | |

NB: Intercepts for ordered logit model of agreement with the main claim are strongly disagree–disagree: 0.20, (0.17–0.24), *p* < 0.001; disagree–neither agree nor disagree: 0.7, (0.60–0.82), *p* < 0.001; neither agree nor disagree–agree: 1.92, (1.64–2.26), *p* < 0.001; agree–strongly agree: 7.49, (6.26–8.97), *p* < 0.001.


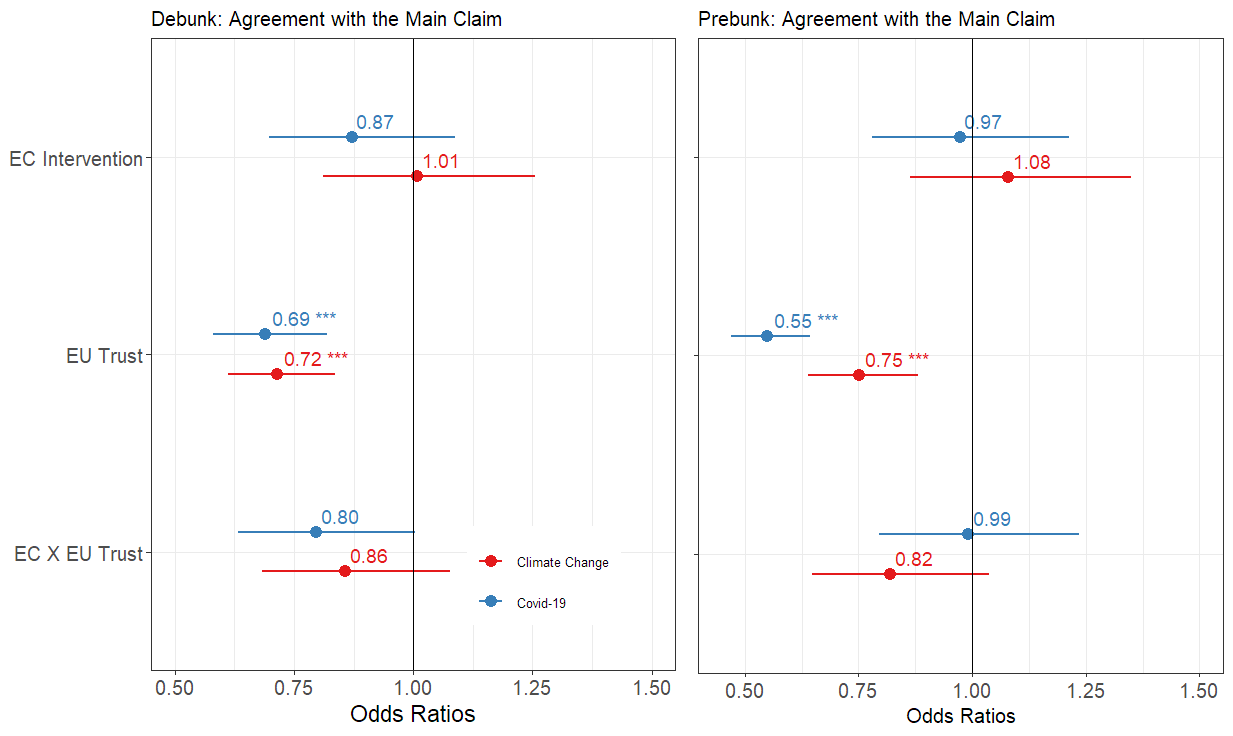


**Figure S-19.** **Effects of the interventions for the average level of trust in the EU and the interaction between source treatment and the level of trust in the EU on agreement with the main claim, by misleading article topic.** Shows the estimates for the interaction of the source information and the level of trust in the EU, both for debunks (left) and prebunks (right), for climate change in red and COVID-19 in blue. The EU trust level (which is not a treatment variable) is de-meaned and standardised such that 0 corresponds to the average level of trust in the EU and estimates for the EU trust level represent the change in the respective DV associated with a one-standard-deviation change in the EU trust level. The model is an ordered logistic regression reporting the odds ratios with heteroscedasticity-robust 95 % confidence intervals. Significance levels: *** *p* < 0.001, ** *p* < 0.01, * *p* < 0.05.

*
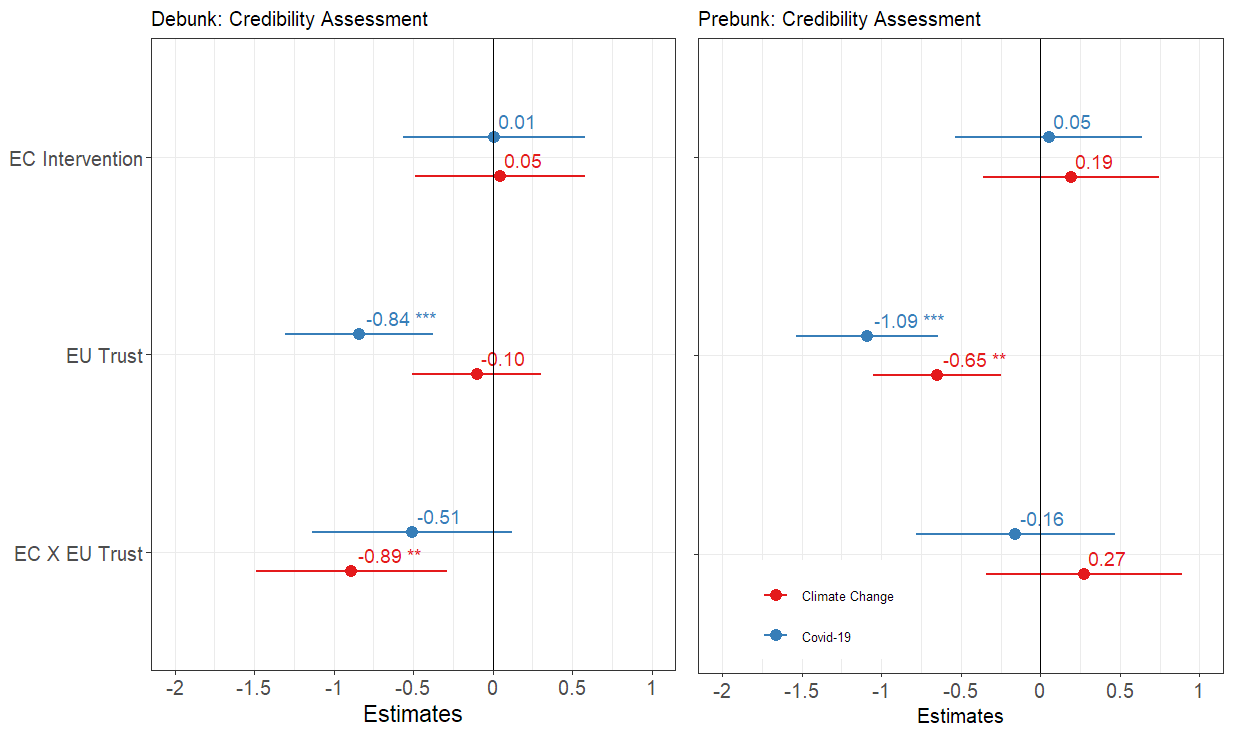
*

**Figure S-20.** **EEffects of the interventions for the average level of trust in the EU and the interaction between source treatment and the level of trust in the EU on credibility assessment, by misleading article topic.** Shows the estimates for the interaction of the source information and the level of trust in the EU, both for debunks (left) and prebunks (right), for climate change in red and COVID-19 in blue. The EU trust level (which is not a treatment variable) is de-meaned and standardised such that 0 corresponds to the average level of trust in the EU and estimates for the EU trust level represent the change in the respective DV associated with a one-standard-deviation change in EU trust level. The model is a linear OLS regression reporting linear estimates with heteroscedasticity-robust 95 % confidence intervals. Significance levels: *** *p* < 0.001, ** *p* < 0.01, * *p* < 0.05.

*
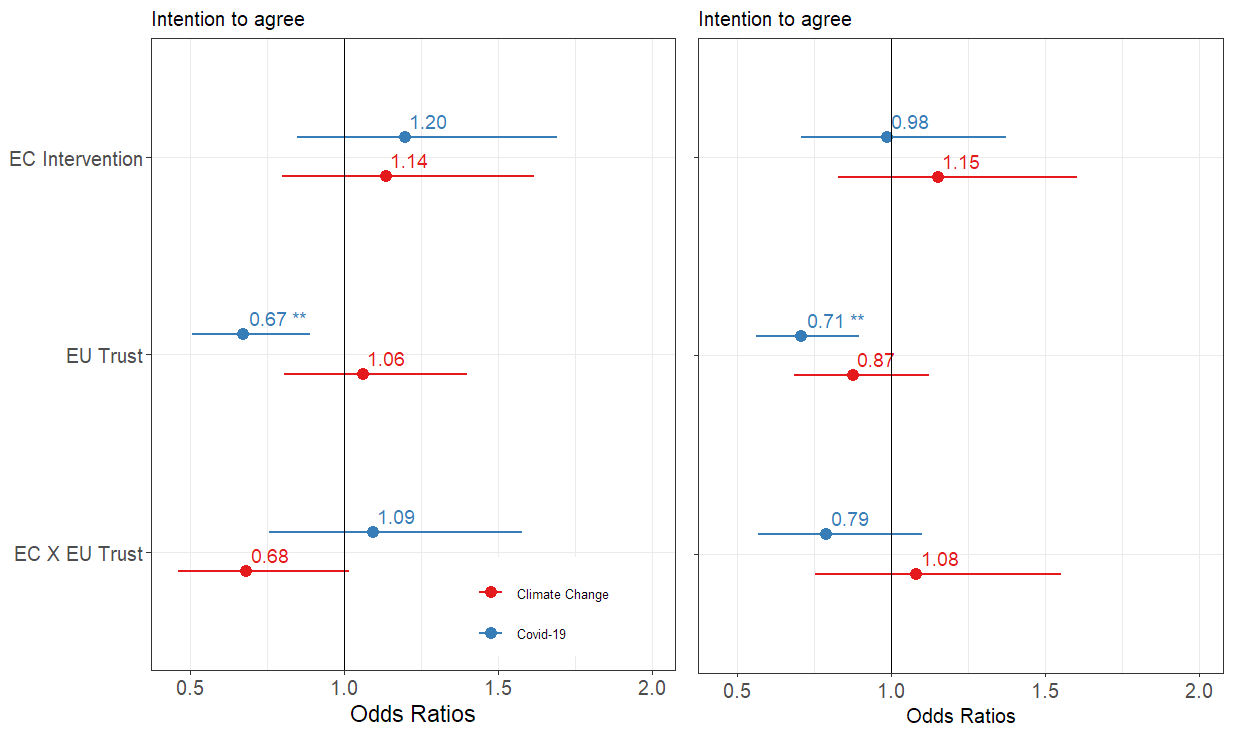
*

**Figure S-21.** **EEffects of the interventions for the average level of trust in the EU and the interaction between source treatment and the level of trust in the EU on the likelihood to express agreement with the misleading article, by article topic.** Shows the estimates for the interaction of the source information and the level of trust in the EU, both for debunks (left) and prebunks (right), for climate change in red and COVID-19 in blue. The EU trust level (which is not a treatment variable) is de-meaned and standardised such that 0 corresponds to the average level of trust in the EU and estimates for the EU trust level represent the change in the respective DV associated with a one-standard-deviation change in the EU trust level. The model is a binary logistic regression reporting the odds ratios with heteroscedasticity-robust 95 % confidence intervals. Significance levels: *** *p* < 0.001, ** *p* < 0.01, * *p* < 0.05.

*
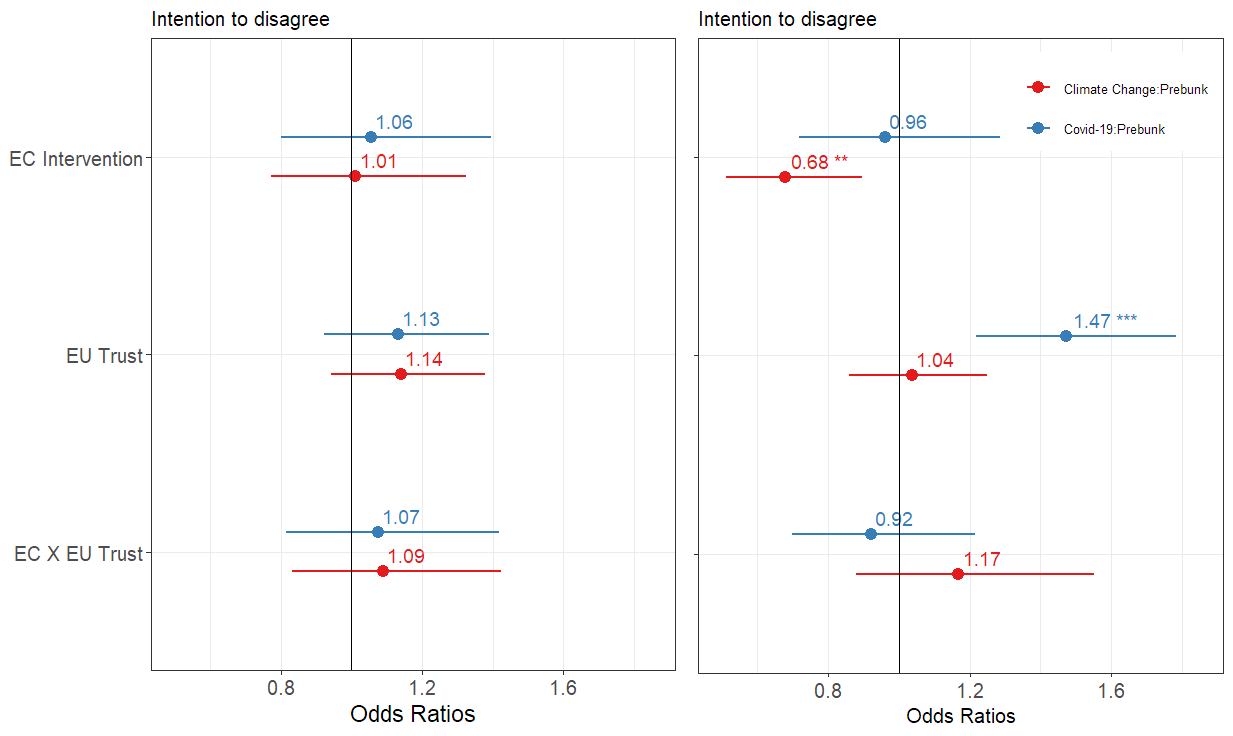
*

**Figure S-22.** **Effects of the interventions for the average level of trust in the EU and the interaction between source treatment and the level of trust in the EU on the likelihood to express disagreement with the misleading** **article, by article topic.** Shows the estimates for the interaction of the source information and the level of trust in the EU, both for debunks (left) and prebunks (right), for climate change in red and COVID-19 in blue. The EU trust level (which is not a treatment variable) is de-meaned and standardised such that 0 corresponds to the average level of trust in the EU and estimates for the EU trust level represent the change in the respective DV associated with a one-standard-deviation change in the EU trust level. The model is a binary logistic regression reporting the odds ratios with heteroscedasticity-robust 95 % confidence intervals. Significance levels: *** *p* < 0.001, ** *p* < 0.01, * *p* < 0.05.

**Table S-15.** **Effects of mentioning the source of the interventions, interaction with the EU trust level and interaction with the topic of the misinformation on beliefs and credibility ratings.** Shows the estimates for the effects of providing the European Commission as the source with the neutral-source condition as the baseline. Models (1) and (2) are ordered logistic regression reporting odds ratios. Models (3) and (4) report linear estimates from OLS models. The EU trust level (which is not a treatment variable) is de-meaned and standardised such that 0 corresponds to the average level of trust in the EU and estimates for the EU trust level represent the change in the respective DV associated with a one-standard-deviation change in the EU trust level. For all models, heteroscedasticity-robust 95 % confidence intervals and *p*-values are provided.

|  | Agreement with main claim | | | | | | Credibility assessment | | | | | |
| --- | --- | --- | --- | --- | --- | --- | --- | --- | --- | --- | --- | --- |
|  | (1) | | | (2) | | | (3) | | | (4) | | |
|  | Debunk | | | Prebunk | | | Debunk | | | Prebunk | | |
| *Predictors* | *Odds ratios* | *CI* | p | *Odds ratios* | *CI* | p | *Estimates* | *CI* | p | *Estimates* | *CI* | p |
| EC (v neutral) | 0.88 | 0.70–1.09 | 0.232 | 0.97 | 0.78–1.21 | 0.802 | 0.01 | – 0.56–0.58 | 0.982 | 0.05 | – 0.53–0.64 | 0.860 |
| EU trust | 0.69 | 0.57–0.85 | < 0.001 | 0.55 | 0.47–0.65 | < 0.001 | – 0.84 | – 1.30 - – 0.38 | < 0.001 | – 1.09 | – 1.53 – 0.64 | < 0.001 |
| Climate change (v COVID-19) | 0.95 | 0.76–1.18 | 0.648 | 1.00 | 0.80–1.24 | 0.966 | 0.23 | – 0.32–0.78 | 0.413 | – 0.10 | – 0.67–0.46 | 0.718 |
| EC × EU trust | 0.80 | 0.62–1.04 | 0.095 | 0.99 | 0.78–1.25 | 0.930 | – 0.51 | – 1.13–0.12 | 0.111 | – 0.16 | – 0.78–0.47 | 0.621 |
| EC × climate change | 1.15 | 0.84–1.57 | 0.373 | 1.11 | 0.81–1.52 | 0.513 | 0.04 | – 0.74–0.82 | 0.921 | 0.14 | – 0.67–0.95 | 0.733 |
| EU trust × climate change | 1.03 | 0.79–1.33 | 0.839 | 1.36 | 1.08–1.71 | 0.009 | 0.74 | 0.12–1.35 | 0.019 | 0.44 | – 0.16–1.03 | 0.151 |
| EC × EU trust × climate change | 1.07 | 0.75–1.53 | 0.717 | 0.83 | 0.59–1.17 | 0.286 | – 0.38 | – 1.24–0.48 | 0.387 | 0.43 | – 0.44–1.30 | 0.335 |
| (Intercept) |  |  |  |  |  |  | 4.75 | 4.34–5.15 | < 0.001 | 4.94 | 4.51–5.36 | < 0.001 |
| Observations | 2 066 | | | 2 012 | | | 2 066 | | | 2 012 | | |
| R^2^ Nagelkerke | 0.116 | | | 0.109 | | | 0.042/0.039 | | | 0.040/0.037 | | |

NB: Intercepts for ordered logit model of agreement with the main claim (debunk) are strongly disagree–disagree: 0.36, (0.30–0.42), *p* < 0.001; disagree–neither agree nor disagree: 1.39, (1.18–1.63), *p* < 0.001; neither agree nor disagree–agree: 4.02, (3.38–4.79), *p* < 0.001; agree–strongly agree: 15.16, (12.06–19.06), *p* < 0.001. Intercepts for ordered logit model of agreement with the main claim (prebunk) are strongly disagree–disagree: 0.36, (0.31–0.43), *p* < 0.001; disagree–neither agree nor disagree: 1.15, (0.97–1.35), *p* = 0.102; neither agree nor disagree–agree: 3.12, (2.61–3.72), *p* < 0.001; agree–strongly agree: 12.62, (10.09–15.78), *p* < 0.001.

**Table S-16.** **Effects of mentioning the source of the interventions, the interaction with the EU trust level and the interaction with the topic of the misinformation on intentions to endorse and criticise.** Shows the estimates for the effects of providing the European Commission as the source with the neutral-source condition as the baseline. All models report odds ratios from binary logistic regressions. The EU trust level (which is not a treatment variable) is de-meaned and standardised such that 0 corresponds to the average level of trust in the EU and estimates for the EU trust level represent the change in the respective DV associated with a one-standard-deviation change in the EU trust level. For all models, heteroscedasticity-robust 95 % confidence intervals and *p*-values are provided.

|  | Intention to agree | | | | | | Intention to disagree | | | | | |
| --- | --- | --- | --- | --- | --- | --- | --- | --- | --- | --- | --- | --- |
|  | (1) | | | (2) | | | (3) | | | (4) | | |
|  | Debunk | | | Prebunk | | | Debunk | | | Prebunk | | |
| *Predictors* | *Odds ratios* | *CI* | p | *Odds ratios* | *CI* | p | *Odds ratios* | *CI* | p | *Odds ratios* | *CI* | p |
| EC (v neutral) | 1.20 | 0.85–1.69 | 0.305 | 0.98 | 0.71–1.36 | 0.927 | 1.06 | 0.80–1.39 | 0.700 | 0.96 | 0.72–1.29 | 0.788 |
| EU trust | 0.67 | 0.52–0.86 | 0.005 | 0.71 | 0.57–0.87 | 0.003 | 1.13 | 0.92–1.39 | 0.235 | 1.47 | 1.21–1.81 | < 0.001 |
| Climate change (v COVID-19) | 0.92 | 0.65–1.31 | 0.657 | 0.83 | 0.60–1.16 | 0.276 | 1.10 | 0.83 1.44 | 0.510 | 1.45 | 1.10–1.92 | 0.009 |
| EC × EU trust | 1.09 | 0.78–1.53 | 0.628 | 0.79 | 0.58–1.07 | 0.159 | 1.07 | 0.81–1.42 | 0.608 | 0.92 | 0.69–1.23 | 0.560 |
| EC × climate change | 0.95 | 0.58–1.55 | 0.832 | 1.17 | 0.73–1.86 | 0.515 | 0.96 | 0.65–1.41 | 0.825 | 0.71 | 0.47–1.05 | 0.090 |
| EU trust × climate change | 1.58 | 1.11–2.25 | 0.023 | 1.24 | 0.90–1.70 | 0.222 | 1.01 | 0.76–1.33 | 0.961 | 0.70 | 0.53–0.93 | 0.010 |
| EC × EU trust  × climate change | 0.62 | 0.39–1.01 | 0.086 | 1.37 | 0.87–2.16 | 0.207 | 1.01 | 0.68–1.50 | 0.948 | 1.27 | 0.85–1.91 | 0.239 |
| (Intercept) | 0.17 | 0.13–0.22 | < 0.001 | 0.24 | 0.19–0.29 | < 0.001 | 0.37 | 0.30–0.45 | < 0.001 | 0.33 | 0.27–0.41 | < 0.001 |
| Observations | 2 066 | | | 2 012 | | | 2 066 | | | 2 012 | | |
| R^2^ Nagelkerke | 0.015 | | | 0.024 | | | 0.006 | | | 0.018 | | |


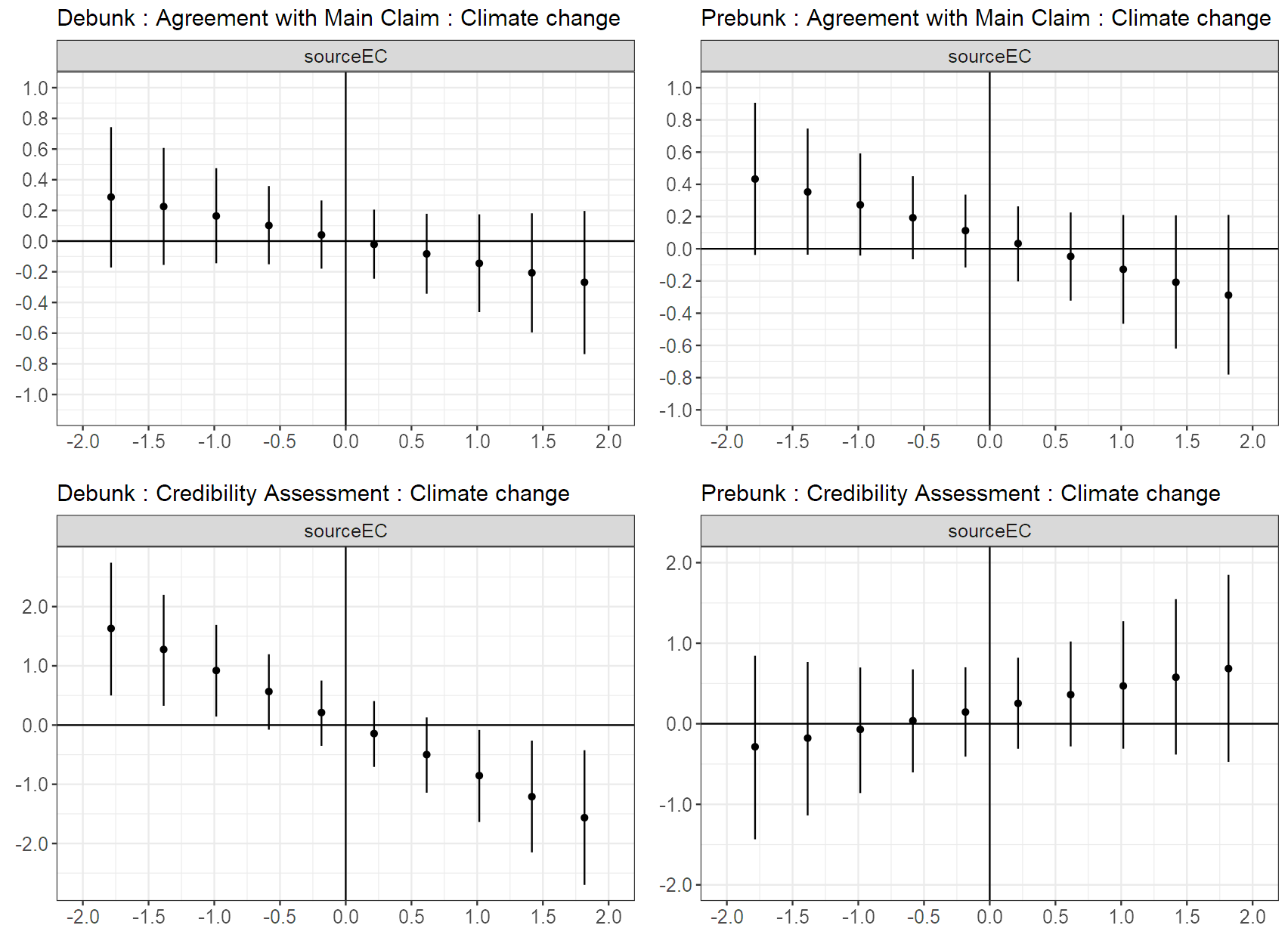


**Figure S-23.** **Effects of mentioning the European Commission as the source of the interventions on beliefs of climate change claims and credibility ratings for different values of EU trust.** The EU trust level is de-meaned and standardised such that 0 on the *x*-axis corresponds to the average level of trust in the EU and estimates for the EU trust level represent the change in the respective DV associated with a one-standard-deviation change in the EU trust level. Shows 95 % confidence intervals (not heteroscedasticity robust).


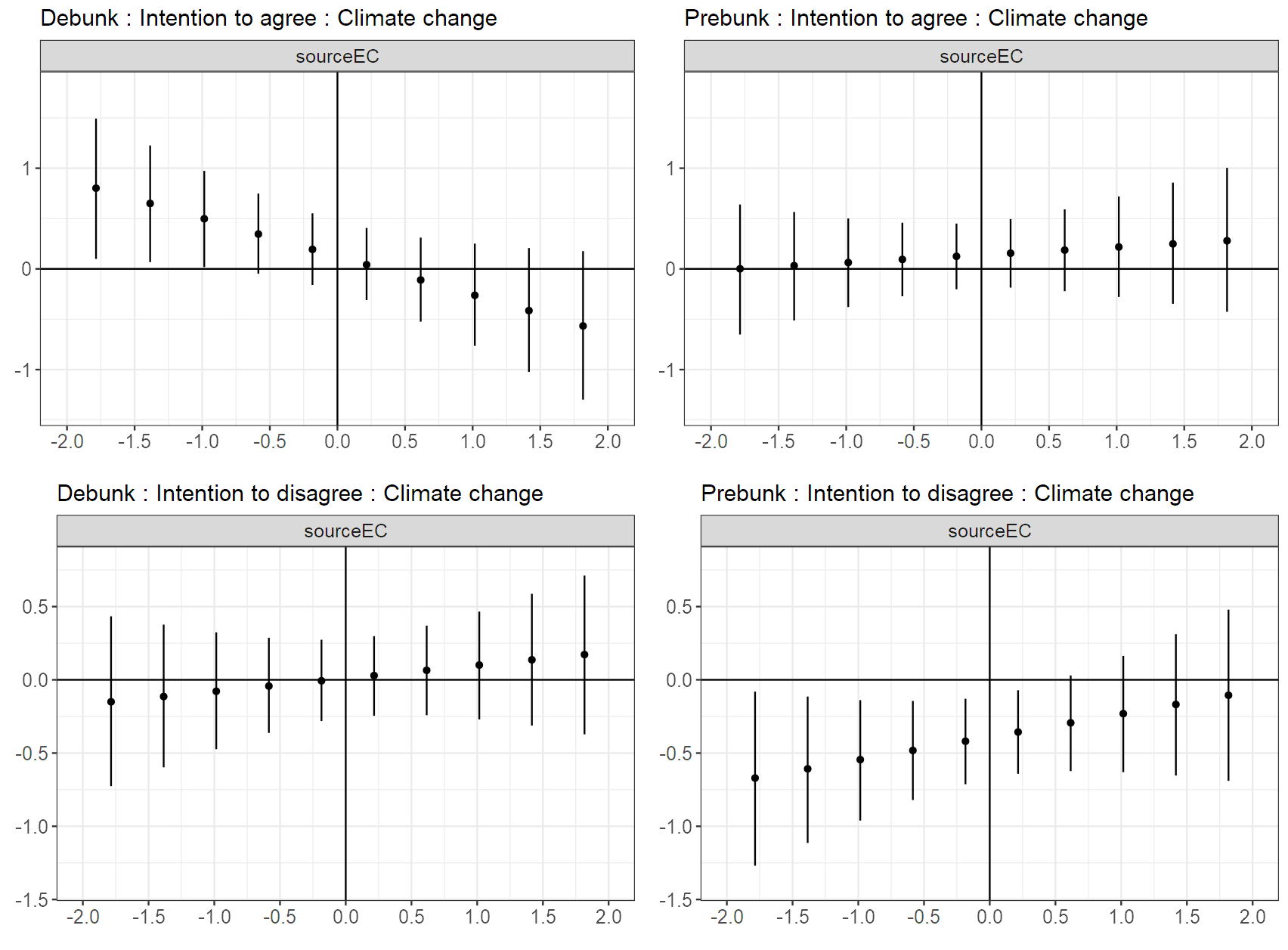


**Figure S-24.** **Effects of mentioning the European Commission as the source of the interventions on intentions to endorse and criticise the misleading** **climate change article for different values of EU trust.** The EU trust level is de-meaned and standardised such that 0 on the *x*-axis corresponds to the average level of trust in the EU and estimates for the EU trust level represent the change in the respective DV associated with a one-standard-deviation change in the EU Trust.trust level. Shows 95 % confidence intervals (not heteroscedasticity robust).


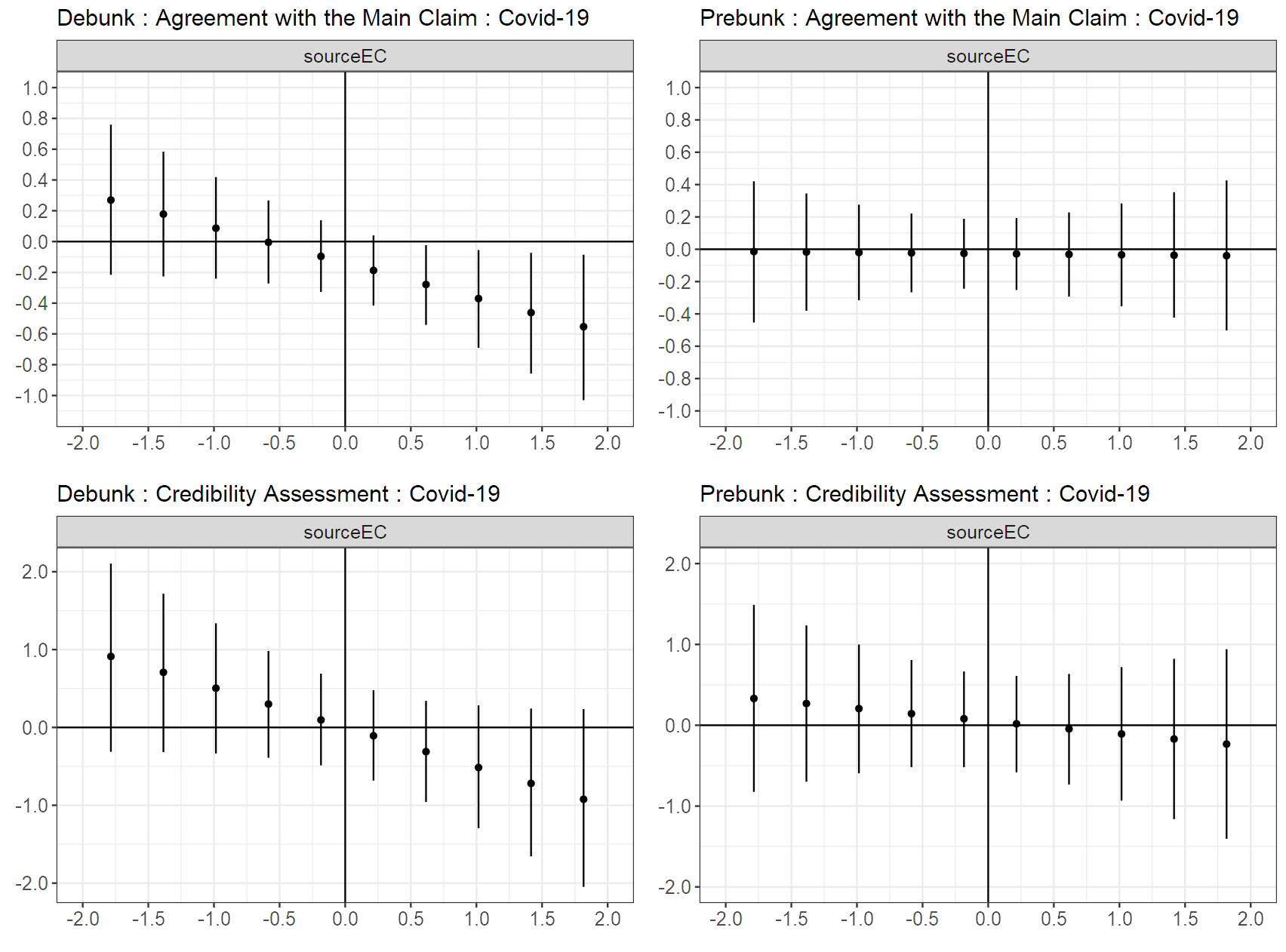


**Figure S-25.** **Effects of mentioning the European Commission as the source of the intervention on beliefs of COVID-19 claims and credibility ratings for different values of EU trust.** The EU trust level is de-meaned and standardised such that 0 on the *x*-axis corresponds to the average level of trust in the EU and estimates for the EU trust level represent the change in the respective DV associated with a one-standard-deviation change in the EU trust level. Shows 95 % confidence intervals (not heteroscedasticity robust).


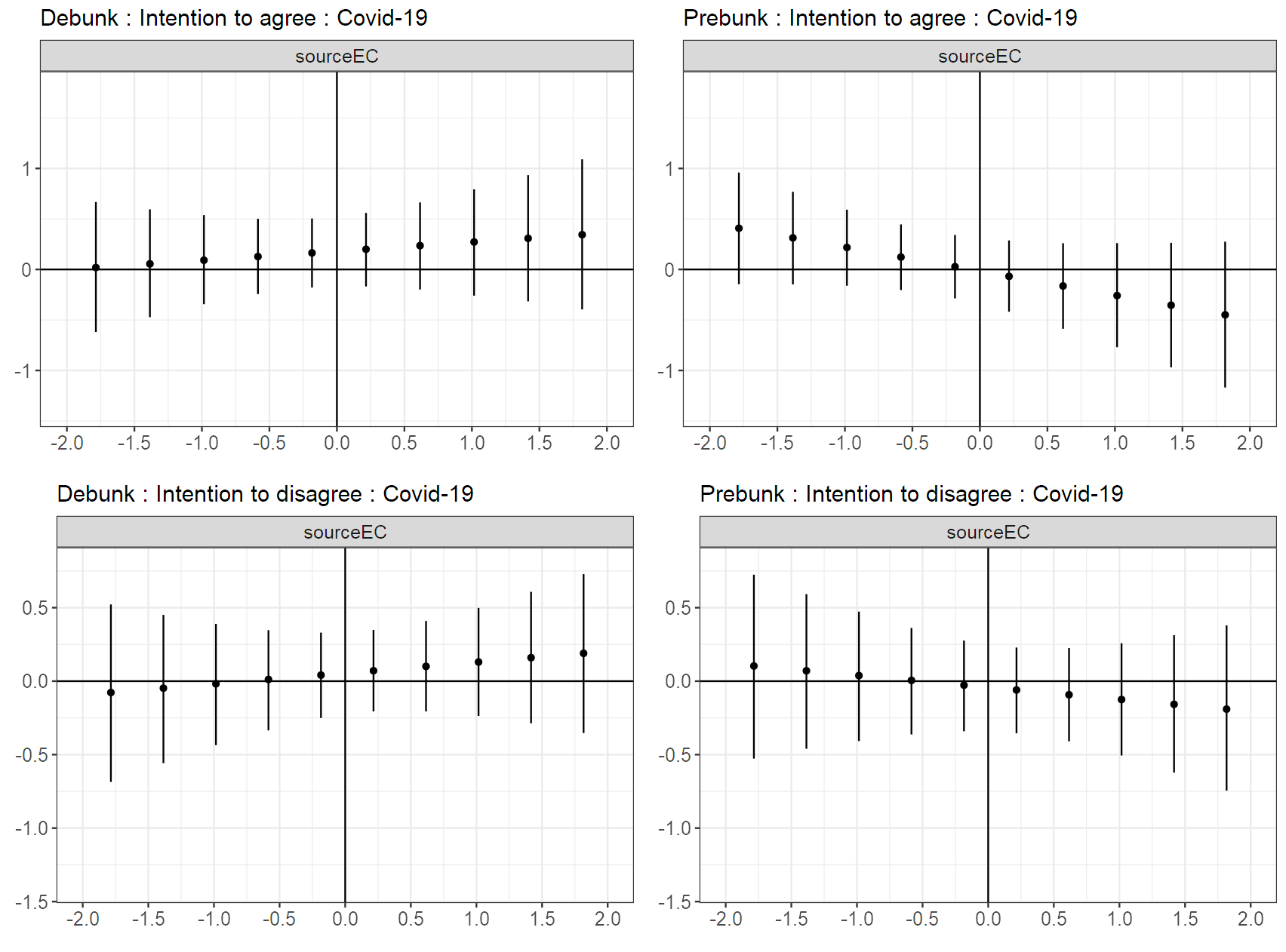


**Figure S-26.** **Effects of mentioning the European Commission as the source of the intervention on intentions to endorse and criticise the** **misleading COVID-19 article for different values of EU trust.** The EU trust level is de-meaned and standardised such that 0 on the *x*-axis corresponds to the average level of trust in the EU and estimates for the EU trust level represent the change in the respective DV associated with a one-standard-deviation change in the EU trust level. Shows 95 % confidence intervals (not heteroscedasticity robust).

## Sample characteristics

**Table S-17. Sample characteristics by country.** Shows the percentages of age, gender and education categories (columns) for the respective countries (rows).

|  | Age | | | | | |  | Gender | |  | Education | | | | | | | | |
| --- | --- | --- | --- | --- | --- | --- | --- | --- | --- | --- | --- | --- | --- | --- | --- | --- | --- | --- | --- |
| Country | 18–24 | 25–34 | 35–44 | 45–54 | 55–64 | > 65 |  | Female | Male |  | Less than primary | Primary | Less than primary | Upper secondary | Upper secondary | Short-cycle tertiary | BA^1^ | MA^1^ | PhD^1^ |
| Germany | 7.63 | 15.26 | 15.56 | 17.01 | 24.03 | 20.52 |  | 51.26 | 48.67 |  | 0.15 | 4.88 | 10.68 | 35.16 | 7.4 | 3.81 | 19.83 | 15.64 | 2.44 |
| Greece | 8.99 | 13.48 | 16.83 | 17.97 | 35.34 | 7.39 |  | 51.94 | 47.91 |  | 0.53 | 1.75 | 3.05 | 21.86 | 6.47 | 9.06 | 41.05 | 14.01 | 2.21 |
| Ireland | 11.96 | 16.05 | 20.68 | 17.13 | 18.13 | 16.05 |  | 51.31 | 46.06 |  | 0.15 | 1 | 4.32 | 22.38 | 13.27 | 8.8 | 31.71 | 16.82 | 1.54 |
| Poland | 8.18 | 17.05 | 20.57 | 15.9 | 27.91 | 10.4 |  | 52.52 | 47.32 |  | 0.38 | 2.06 | 16.36 | 20.57 | 9.86 | 1.83 | 10.86 | 37.08 | 0.99 |

^1^ Or equivalent.

**Table S-18. Regional distribution of the sample in Germany.** Shows the percentages of the sample coming from the respective regions.

| Region | Percentage |
| --- | --- |
| Baden-Württemberg | 12.28 |
| Bavaria | 15.71 |
| Berlin | 5.11 |
| Brandenburg | 3.05 |
| Bremen | 0.76 |
| Hamburg | 2.44 |
| Hessen | 7.78 |
| Mecklenburg-Western Pomerania | 1.98 |
| Lower Saxony | 9.99 |
| North Rhine-Westphalia | 21.21 |
| Rhineland-Palatinate | 4.58 |
| Saarland | 1.22 |
| Saxony | 5.11 |
| Saxony-Anhalt | 2.82 |
| Schleswig-Holstein | 3.36 |
| Thuringia | 2.59 |

**Table S-19. Regional distribution of the sample in Greece.** Shows the percentages of the sample coming from the respective regions.

| Region | Percentage |
| --- | --- |
| Attica | 41.81 |
| Continental Greece | 2.67 |
| Central Macedonia | 20.56 |
| Crete | 4.04 |
| East Macedonia, Thrace | 5.41 |
| Epirus | 2.97 |
| Ionian Islands | 1.29 |
| North Aegean | 1.29 |
| Peloponnese | 6.78 |
| South Aegean | 2.21 |
| Thessaly | 5.71 |
| Western Greece | 2.67 |
| West Macedonia | 2.59 |

**Table S-20. Regional distribution of the sample in Ireland.** Shows the percentages of the sample coming from the respective regions.

| Region | Percentage |
| --- | --- |
| Eastern and Midland | 42.44 |
| Northern and Western | 20.45 |
| Southern | 37.11 |

**Table S-21. Regional distribution of the sample in Poland.** Shows the percentages of the sample coming from the respective regions.

| Region | Percentage |  |
| --- | --- | --- |
| Dolnośląskie | 6.27 |  |
| Kujawsko-pomorskie | 5.89 |  |
| łódzkie | 6.5 |  |
| lubelskie | 6.12 |  |
| lubuskie | 1.91 |  |
| małopolskie | 7.87 |  |
| mazowieckie-Regional | 6.73 |  |
| opolskie | 2.14 |  |
| podkarpackie | 4.82 |  |
| podlaskie | 3.52 |  |
| pomorskie | 5.73 |  |
| śląskie | 13.69 |  |
| świętokrzyskie | 2.91 |  |
| Warsaw-Capital | | 8.87 |
| warmińsko-mazurskie | 2.91 |  |
| wielkopolskie | 9.4 |  |
| zachodniopomorskie | 4.74 |  |

## Robustness checks

**Table S-22.** **Effects of interventions on the four main outcome variables including control variables.** Shows the estimates for the intervention effects with the control condition as the baseline. Model (1) is an ordered logistic regression reporting the odds ratios. Model (2) reports linear estimates from an OLS model. Models (3) and (4) report odds ratios from a binary logistic regression. Estimates for the EU trust level represent the change in the respective DV associated with a one-standard-deviation change in the EU trust level. For all models, heteroscedasticity-robust 95 % confidence intervals and *p*-values are provided. Control variables: age, gender, level of education, country of residence, political ideology, trust in the national government, general trust, a trust index in the EU, need for cognition, frequency of social media use, perceived frequency of encountering misinformation, perceived importance of sharing true information and confidence in identifying misinformation, as well as responses to the comprehension check questions and a manipulation check regarding the correct identification of the intervention source.

|  | (1) | | | (2) | | | (3) | | | (4) | | |
| --- | --- | --- | --- | --- | --- | --- | --- | --- | --- | --- | --- | --- |
|  | Agreement with the main claim | | | Credibility assessment | | | Intention to agree | | | Intention to disagree | | |
| *Predictors* | *Odds ratios* | *CI* | p | *Estimates* | *CI* | p | *Odds ratios* | *CI* | p | *Odds ratios* | *CI* | p |
| Neutral debunk | 0.44 | 0.36–0.53 | < 0.001 | – 1.53 | – 1.99 to – 1.07 | < 0.001 | 0.43 | 0.32–0.59 | < 0.001 | 1.39 | 1.08–1.79 | 0.010 |
| Commission debunk | 0.40 | 0.33–0.49 | < 0.001 | – 1.52 | – 1.96 to – 1.08 | < 0.001 | 0.53 | 0.40–0.70 | < 0.001 | 1.44 | 1.14–1.82 | 0.003 |
| Neutral prebunk | 0.56 | 0.45–0.68 | < 0.001 | – 1.13 | – 1.60 to – 0.67 | < 0.001 | 0.60 | 0.44–0.80 | 0.001 | 1.39 | 1.08–1.79 | 0.010 |
| Commission prebunk | 0.54 | 0.45–0.65 | < 0.001 | – 1.28 | – 1.70 to – 0.85 | < 0.001 | 0.67 | 0.51–0.87 | 0.003 | 1.04 | 0.83–1.32 | 0.728 |
| EU trust | 0.99 | 0.89–1.09 | 0.794 | 0.07 | – 0.17–0.30 | 0.581 | 1.11 | 0.96–1.27 | 0.165 | 1.10 | 0.98–1.24 | 0.130 |
| Intercept |  |  |  | 10.46 | 8.92–12.00 | < 0.001 | 1.09 | 0.42–2.81 | 0.861 | 0.15 | 0.07–0.35 | < 0.001 |
| Control variables | Yes | | | Yes | | | Yes | | | Yes | | |
| Observations | 4 562 | | | 4 562 | | | 4 562 | | | 4 562 | | |
| R^2^ Nagelkerke | 0.533 | | | 0.233/0.226 | | | 0.095 | | | 0.035 | | |

NB: Intercepts for ordered logit model of agreement with the main claim are strongly disagree–disagree: 0.02, (0.01–0.04), *p* < 0.001; disagree–neither agree nor disagree: 0.09, (0.05–0.18), *p* < 0.001; neither agree nor disagree–agree: 0.28, (0.14–0.55), *p* < 0.001; agree–strongly agree: 1.15, (0.59–2.26), *p* = 0.675.

**Table S-23.** **Effects of mentioning the European Commission as the source of the interventions and the interaction with the EU trust level on beliefs and credibility ratings including control variables.** Shows the estimates for the effects of providing the Commission as the source with the neutral-source condition as the baseline. Model (1) and (2) are sordered logistic regression reporting the odds ratios. Models (3) and (4) report linear estimates from OLS models. The EU trust level is de-meaned and standardised such that 0 corresponds to the average level of trust in the EU and estimates for the EU trust level represent the change in the respective DV associated with a one-standard-deviation change in the EU Trus trust ttrust level. For all models, heteroscedasticity-robust 95 % confidence intervals and *p*-values are provided. Control variables: age, gender, level of education, country of residence, political ideology, trust in the national government, general trust, a trust index in the EU, need for cognition, frequency of social media use, perceived frequency of encountering misinformation, perceived importance of sharing true information and confidence in identifying misinformation, as well as responses to the comprehension check questions and a manipulation check regarding the correct identification of the intervention source.

|  | Agreement with the main claim | | | | | | Credibility assessment | | | | | |
| --- | --- | --- | --- | --- | --- | --- | --- | --- | --- | --- | --- | --- |
|  | (1) | | | (2) | | | (3) | | | (4) | | |
| Intervention | Debunk | | | Prebunk | | | Debunk | | | Prebunk | | |
| *Predictors* | *Odds ratios* | *CI* | p | *Odds ratios* | *CI* | p | *Estimates* | *CI* | p | *Estimates* | *CI* | p |
| Intercept |  |  |  |  |  |  | 11.27 | 8.81–13.73 | < 0.001 | 7.85 | 5.43–10.27 | < 0.001 |
| Commission (v neutral) | 0.93 | 0.78–1.11 | 0.439 | 1.00 | 0.83–1.21 | 0.985 | 0.06 | – 0.32–0.45 | 0.752 | – 0.18 | – 0.58–0.23 | 0.386 |
| EU trust | 1.05 | 0.86–1.28 | 0.620 | 1.12 | 0.94–1.34 | 0.204 | 0.31 | – 0.11–0.72 | 0.148 | 0.06 | – 0.34–0.47 | 0.752 |
| Commission Intervention × EU trust | 0.87 | 0.71–1.07 | 0.192 | 0.88 | 0.73–1.07 | 0.200 | –0.51 | – 0.94 to – 0.08 | 0.019 | 0.18 | – 0.24–0.60 | 0.398 |
| Control variables | Yes | | | Yes | | | Yes | | | Yes | | |
| Observations | 1 818 | | | 1 801 | | | 1 818 | | | 1 801 | | |
| R^2^ Nagelkerke | 0.540 | | | 0.517 | | | 0.235/0.217 | | | 0.262/0.245 | | |

NB: Intercepts for ordered logit model of agreement with the main claim (debunk) are strongly disagree–disagree: 0.02, (0.01–0.06), *p* < 0.001; disagree–neither agree nor disagree: 0.10, (0.03–0.31), *p* < 0.001; neither agree nor disagree–agree: 0.32, (0.10–1.02), *p* = 0.054; agree–strongly agree: 1.27, (0.40–4.04), *p* = 0.688. Intercepts for ordered logit model of agreement with the main claim (prebunk) are strongly disagree–disagree: 0.04, (0.01–0.12), *p* < 0.001; disagree–neither agree nor disagree: 0.16, (0.05–0.46), *p* = 0.001; neither agree nor disagree–agree: 0.48, (0.17–1.40), *p* = 0.178; agree–strongly agree: 2.07, (0.71–6.05), *p* = 0.181.

**Table S-24.** **Effects of mentioning the European Commission as the source of the interventions and the interaction with the EU trust level on intentions to agree and disagree.** Shows the estimates for the effects of providing the Commission as the source with the neutral-source condition as the baseline. All models report odds ratios from binary logistic regressions. The EU trust level is de-meaned and standardised such that 0 corresponds to the average level of trust in the EU and estimates for the EU trust level represent the change in the respective DV associated with a one-standard-deviation change in the EU trust level. For all models, heteroscedasticity-robust 95 % confidence intervals and *p*-values are provided. Control variables: age, gender, level of education, country of residence, political ideology, trust in the national government, general trust, a trust index in the EU, need for cognition, frequency of social media use, perceived frequency of encountering misinformation, perceived importance of sharing true information and confidence in identifying misinformation, as well as responses to the comprehension check questions and a manipulation check regarding the correct identification of the intervention source.

|  | Intention to agree | | | | | | Intention to disagree | | | | | |
| --- | --- | --- | --- | --- | --- | --- | --- | --- | --- | --- | --- | --- |
|  | (1) | | | (2) | | | (3) | | | (4) | | |
| Intervention | Debunk | | | Prebunk | | | Debunk | | | Prebunk | | |
| *Predictors* | *Odds ratios* | *CI* | p | *Odds ratios* | *CI* | p | *Estimates* | *CI* | p | *Estimates* | *CI* | p |
| Intercept | 1.00 | 0.19–5.27 | 0.997 | 0.33 | 0.07–1.55 | 0.163 | 0.32 | 0.09–1.17 | 0.083 | 0.13 | 0.03–0.49 | 0.002 |
| Commission (v neutral) | 1.23 | 0.93–1.63 | 0.164 | 1.07 | 0.82–1.42 | 0.619 | 1.04 | 0.84–1.29 | 0.713 | 0.70 | 0.56–0.89 | 0.003 |
| EU trust | 1.14 | 0.86–1.50 | 0.388 | 1.16 | 0.90–1.50 | 0.261 | 1.06 | 0.86–1.32 | 0.586 | 1.08 | 0.87–1.34 | 0.498 |
| Commission × EU trust | 0.89 | 0.68–1.16 | 0.427 | 0.92 | 0.71–1.18 | 0.544 | 1.11 | 0.89–1.37 | 0.355 | 1.08 | 0.86–1.34 | 0.511 |
| Control variables | Yes | | | Yes | | | Yes | | | Yes | | |
| Observations | 2 066 | | | 2 012 | | | 2 066 | | | 2 012 | | |
| R^2^ Nagelkerke | 0.011 | | | 0.016 | | | 0.006 | | | 0.012 | | |

## Experimental materials

All experimental materials can be found at <https://osf.io/7kytz/> .


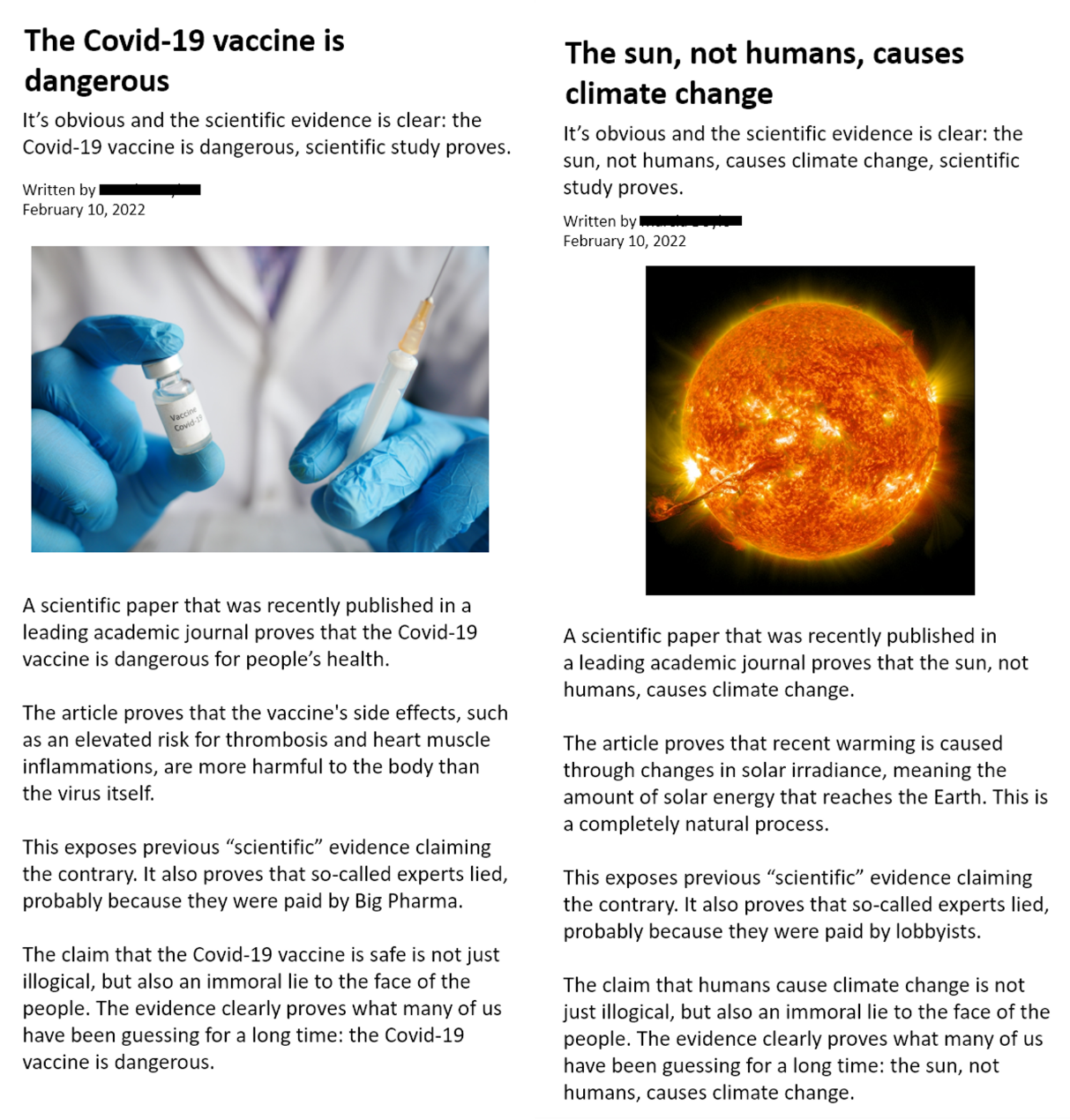


**Figure S-27. Examples of two misleading articles used in the experiment**. Left: COVID-19. Right: climate change.


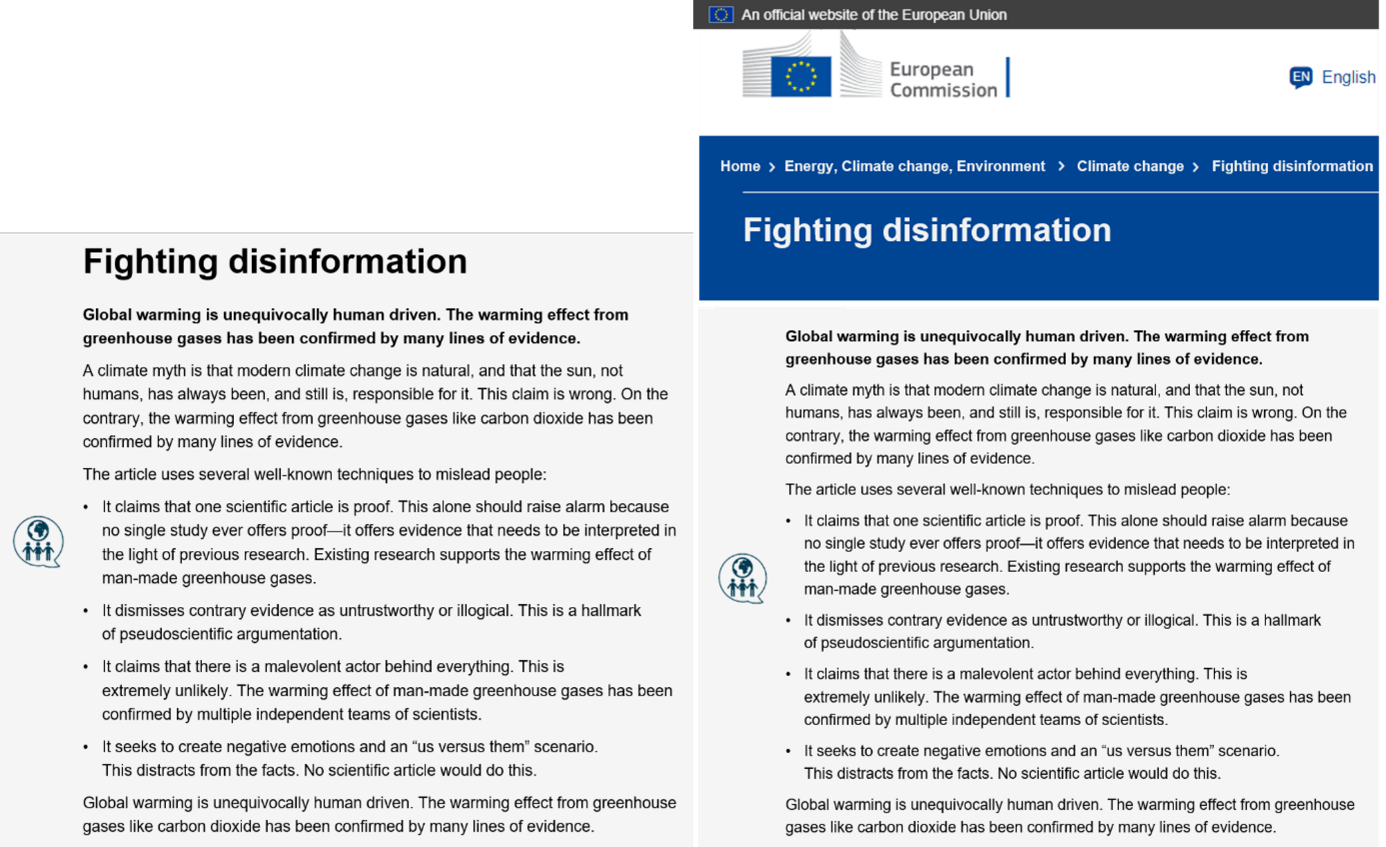


**Figure S-28. Examples of two debunks used in the experiment.** Left: no source. Right: European Commission source.


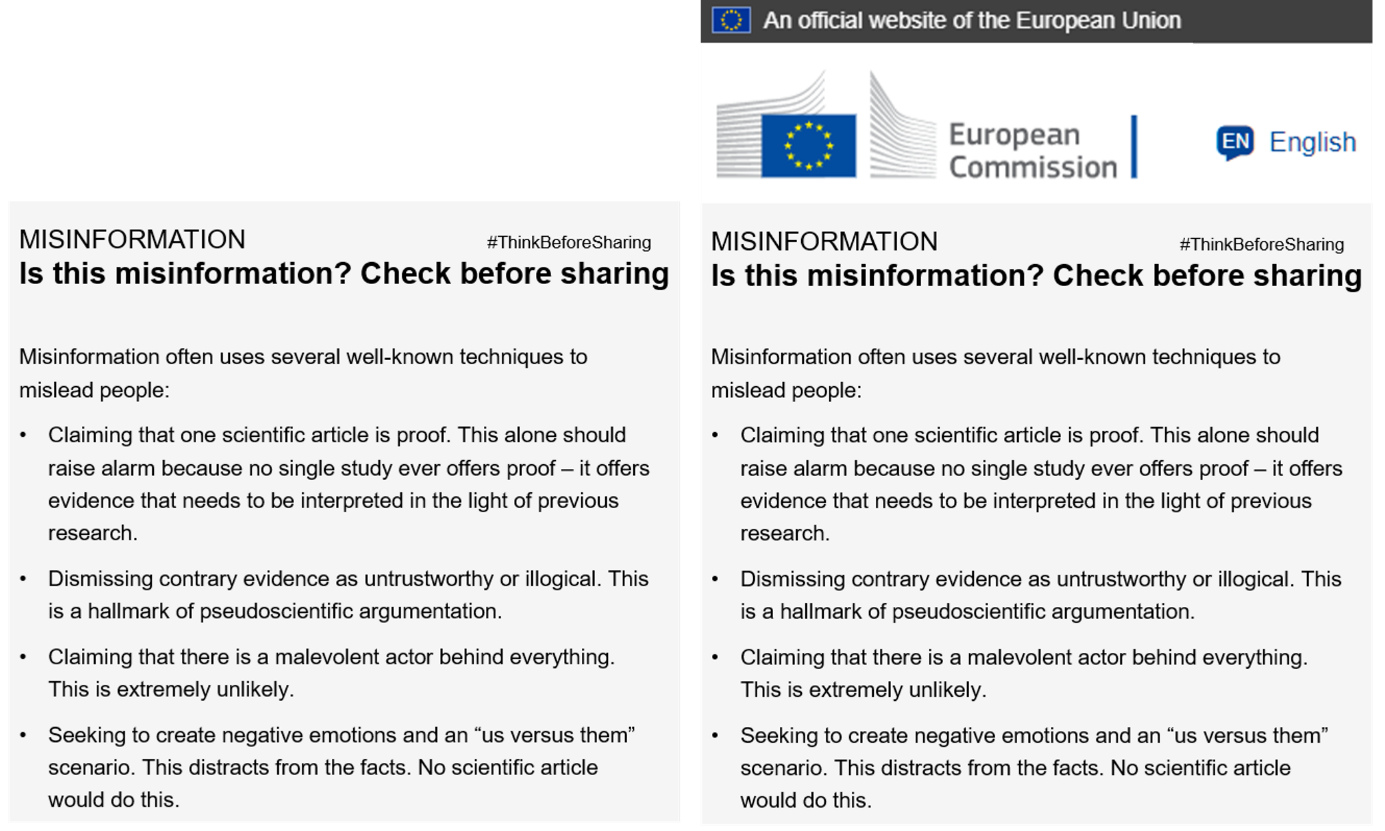


**Figure S-29. Examples of prebunks used in the experiment.** Left: no source. Right: European Commission source.

**Table S-25. Data from Eurobarometer**^78^ **underlying country selection for the experiment**

|  | | How often do you encounter fake news? | | How confident are you in identifying fake news? | |  |
| --- | --- | --- | --- | --- | --- | --- |
| Country | *n* | ‘Every day or almost every day’ | ‘At least once a week’ | ‘Confident’ | ‘Not confident’ | Region |
| Germany | 1000 | 24 % | 57 % | 71 % | 27 % | Central |
| Greece | 1001 | 55 % | 74 % | 64 % | 35 % | Southern |
| Ireland | 1002 | 33 % | 69 % | 84 % | 15 % | Western |
| Poland | 1000 | 48 % | 75 % | 71 % | 26 % | Central |
| EU | 26576 | 37 % | 68 % | 71 % | 26 % |  |

## Questionnaire

1. We will now ask you about your thoughts and feelings regarding the European Union. In this context, the ‘European Union’ refers to its main institutions, which are the European Commission, the European Parliament and the European Council. [Five-point Likert: 1, Strongly disagree, to 5, Strongly agree, Prefer not to say]
   1. Overall, the EU is competent and efficient
   2. The EU usually carries out its duties poorly (reverse coded)
   3. The EU usually acts in its own interests (reverse coded)
   4. The EU wants to do its best to serve Europe
   5. The EU is generally free of corruption
   6. The EU work is open and transparent
2. Please respond to the following questions: [10-point scale: 1, I do not trust it at all, to 10, I trust it completely, Prefer not to say]
   - How much trust do you have in the European Union?
   - How much trust do you have in your national government?
3. Generally speaking, would you say that most people can be trusted or that you need to be very careful in dealing with people? [Three options: Most people can be trusted, Need to be very careful, Prefer not to say]
4. Please indicate to what extent you agree or disagree with the following statement: [10-point scale: 1, Left, to 10, Right, Prefer not to say]
   - In political matters people talk of ‘the left’ and ‘the right’. How would you place your views on this scale?
5. Please indicate to what degree you agree or disagree with the following statements concerning the [control/prebunk/debunk] text that you have seen before (shown again below). [Five-point Likert: 1, Strongly disagree, to 5, Strongly agree, Prefer not to say]
   1. The message appears relevant to me
   2. I can use this message to make good decisions
   3. The message appears authentic to me
   4. The message grabbed my attention
   5. The message wants to manipulate me
6. Who do you think was its source? [Four options: No one, The European Commission, The University of Hamburg, I don’t know]
7. How often, on average, do you use online social media (e.g. Facebook, Twitter, Instagram, TikTok, etc.) [Five options: Seldom or never, Several times a month, At least once a week, Every day or almost every day, Prefer not to say]
8. How often do you come across news or information that you believe misrepresent reality or are even false? [Five options: Seldom or never, Several times a month, At least once a week, Every day or almost every day, Prefer not to say]
9. How important is it to you that you only share news articles on social media (e.g. Facebook, Twitter, Instagram, TikTok, etc.) if they are accurate? [Five-point Likert: 1, Very unimportant, to 5, Very important]
10. How confident are you that you are able to identify news or information that misrepresent reality or are even false? [Four-point Likert: 1, Not at all confident, to 4, Very confident, Prefer not to say]
11. Please indicate the degree to which the following statements are characteristic of you: [Five-point Likert: 1, Extremely uncharacteristic of me, to 5, Extremely characteristic of me, Prefer not to say]
    1. I like to have the responsibility of handling a situation that requires a lot of thinking
    2. I would prefer complex to simple problems
    3. Thinking is not my idea of fun
    4. I would rather do something that requires little thought than something that is sure to challenge my thinking abilities
    5. I really enjoy a task that involves coming up with new solutions to problems
    6. I would prefer a task that is intellectual, difficult and important to one that is somewhat important but does not require much thought

## Debriefing text

*For people in the control and prebunk conditions:*

Please be aware that the first article titled [article title] you saw previously was fabricated and contained incorrect information. Please carefully read the following correction. After reading, check the box indicating that you read the article before advancing.

[Show corresponding debunk]

*For everyone:*

Thank you for taking the time to respond to this survey. The goal of this study was to find out how effective different ways to expose and correct (debunk) false information (misinformation, fake news) are. To investigate this, we asked you to read four articles which contained false claims and could thus be considered misinformation or fake news. At some point, you were then shown four articles correcting these false claims and explaining the deceptive strategies used in them. The nature of the phenomenon we are investigating required minor deception on our part. Specifically, we presented the fake news articles without labelling them as such. In this way, we may have led you to believe them to be accurate. To investigate the effectiveness of correcting (debunking) information, there was no other way than to expose you to fake news and only correct them at a later point in time. This is sometimes necessary in this type of research. If we tell people about the articles being fake in advance, we could not investigate how debunks work for people who encounter fake news without realising it. Your participation is greatly appreciated by the researchers involved and will contribute to advancing the research in this field. If you have any questions about this study, please contact us. Finally, we urge you not to discuss this study with anyone else who is currently participating or might participate at a future point in time. As you can certainly appreciate, we will not be able to examine the effectiveness of correcting and debunking misinformation for participants who know about the true purpose of the project beforehand. Thank you!
